# Combined experimental and theoretical studies of regio- and stereoselectivity in reactions of β-isoxazolyl- and β-imidazolyl enamines with nitrile oxides

**DOI:** 10.3762/bjoc.12.233

**Published:** 2016-11-15

**Authors:** Ilya V Efimov, Marsel Z Shafikov, Nikolai A Beliaev, Natalia N Volkova, Tetyana V Beryozkina, Wim Dehaen, Zhijin Fan, Viktoria V Grishko, Gert Lubec, Pavel A Slepukhin, Vasiliy A Bakulev

**Affiliations:** 1 TOS Department, Ural Federal University named after the first President of Russia B.N. Yeltsin, 19 Mira str., 620002 Yekaterinburg, Russia; 2 Chemistry Department, University of York, Heslington, York, YO10 5DD, UK; 3 Molecular Design and Synthesis, Department of Chemistry, KU Leuven, Celestijnenlaan 200F, 3001 Leuven, Belgium; 4 State Key Laboratory of Elemento-Organic Chemistry Nankai University, Collaborative Innovation Center of Chemical Science and Engineering 300071 Tianjin, China; 5 Laboratory of Biologically Active Compounds, Institute of Technical Chemistry Ural Branch of Russian Academy of Sciences, 3 Academician Korolev str., Perm, Russia; 6 Medical University of Vienna, Department of Pediatrics, 14 Lazarettgasse, 1090 Vienna, Austria; 7 I. Ya. Postovsky Institute of Organic Synthesis, Ural Branch of Russian Academy of Sciences, 20 S. Kovalevskaya str., 620990 Yekaterinburg, Russia

**Keywords:** β-azolyl enamine, [3 + 2]-cycloaddition, isoxazole, isoxazoline, nitrile oxide

## Abstract

Reactions of β-azolyl enamines and nitrile oxides were studied by both experimental and theoretical methods. (*E*)-β-(4-Nitroimidazol-5-yl), (5-nitroimidazol-4-yl) and isoxazol-5-yl enamines smoothly react regioselectively at room temperature in dioxane solution with aryl, pyridyl, and cyclohexylhydroxamoyl chlorides without a catalyst or a base to form 4-azolylisoxazoles as the only products in good yields. The intermediate 4,5-dihydroisoxazolines were isolated as *trans* isomers during the reaction of (*E*)-β-imidazol-4-yl enamines with aryl and cyclohexylhydroxamoyl chlorides. Stepwise and concerted pathways for the reaction of β-azolyl enamines with hydroxamoyl chlorides were considered and studied at the B3LYP/Def2-TZVP level of theory combined with D3BJ dispersion correction. The reactions of benzonitrile oxide with both *E*- and *Z*-imidazolyl enamines have been shown to proceed stereoselectively to form *trans-* and *cis*-isoxazolines, respectively. The preference of *E*-isomers over *Z*-isomers, driven by the higher stability of the former, apparently controls the stereoselectivity of the investigated cycloaddition reaction with benzonitrilе oxide. Based on the reactivity of azolyl enamines towards hydroxamoyl chlorides, a novel, effective catalyst-free method was elaborated to prepare 4-azolyl-5-substituted isoxazoles that are otherwise difficult to obtain.

## Introduction

The biological activity and technically useful properties of isoxazoles have made them the focus of both medicinal and materials chemistry over the years [1]. Isoxazoles have been found in natural products [1], and they exhibit anticancer [2], antiviral [1], anti-inflammatory [3], antidiabetic [4], anti-Alzheimer [5] and many other types of biological activity [6]. Isoxazolines and isoxazoles have been applied as chemosensors, liquid crystalline compounds, ligands for asymmetric synthesis, and they are also convenient reagents in organic synthesis [1]. Although bicyclic assemblies of azoles exhibit interesting chemical properties and biological activities [1,7–11] isoxazoles conjugated to other azole rings are poorly presented in the literature in comparison with monocyclic and fused derivatives [1,6].

**Figure 1:**
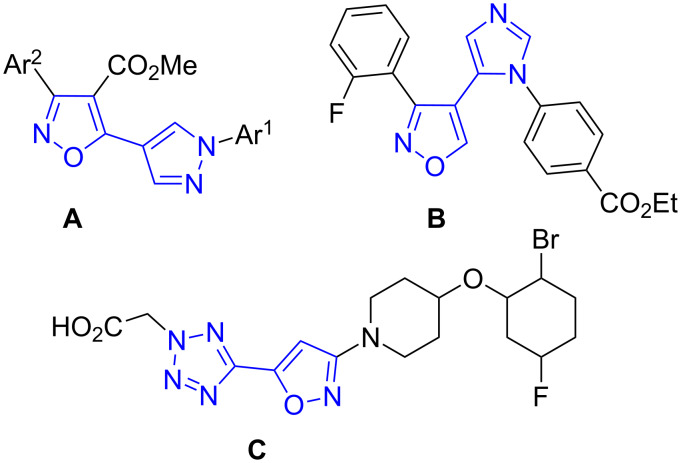
Biologically active isoxazoles conjugated to other azole rings.

Recently isoxazoles conjugated to pyrazole **A** [12], imidazole **B** [13] and tetrazole **C** [4] rings were found as promising candidates for anticancer and antidiabetic drugs and for the treatment of cognitive disorder (Figure 1). It makes prospective the synthesis of new derivatives of isoxazoles conjugated with other azole rings.

**Scheme 1:**
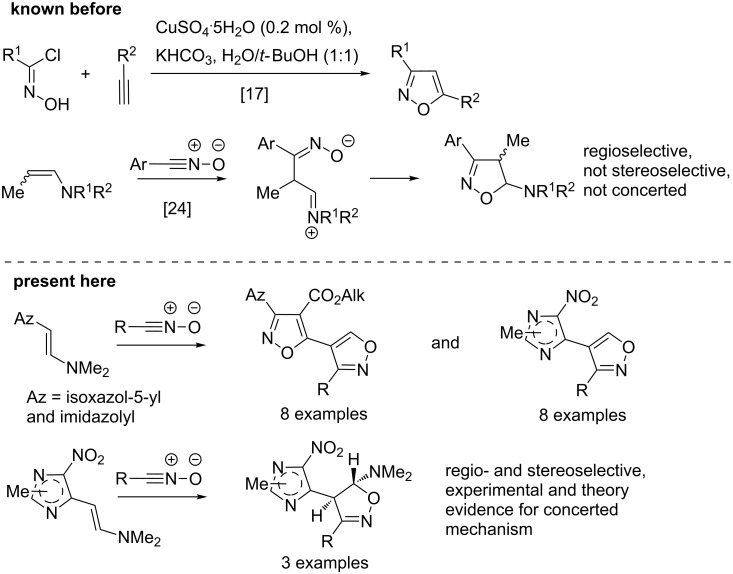
Reactions of azolyl enamines with nitrile oxides.

The few synthetic methods for 4-(azol-5-yl)isoxazoles published in the literature involve the formation of either azole or isoxazole rings in the final step [1,13–14]. Cyclization reactions of 2-azolyl-1,3-dicarbonyl compounds with hydroxylamine and cyanomethylazoles with hydroxamoyl chlorides are used for the synthesis of a few representatives of 4-(azol-5-yl)isoxazoles [1,4,12–16]. Cycloaddition reactions of azolylacetylenes with nitrile oxides are an alternative method for the synthesis of this type of compounds [14,17] (Scheme 1). Despite of good yields this method has serious limitations for the synthesis of azolylisoxazoles due to the poor availability of the starting materials. Therefore, the search of new regioselective routes to azolylisoxazoles remains a synthetic challenge. We turned our attention to the reaction of enamines with nitrile oxides (or their precursors, hydroxamoyl chlorides). The reaction has been shown to take place regioselectively to form only one of two possible regioisomers [18–26].

Therefore, this holds some promise for the development of an efficient method based on this reaction for the synthesis of monocyclic, fused and conjugated isoxazoles. At the same time, there are few reports on the systematic study of this reaction [23–24].

A general discussion about the reasons of regioselectivity and stereoselectivity for this reaction is lacking from the literature. To the best of our knowledge there are no examples for the synthesis of 4-isoxazolyl- and imidazolylisoxazoles and imidazolylisoxazolines by this reaction.

We report here the results of experimental and theoretical studies for the reaction of β-azolyl enamines bearing isoxazol-5-yl, imidazol-4-yl and imidazol-5-yl moieties with aryl, pyridyl and cyclohexylhydroxamoyl chlorides, pointing to a concerted mechanism of this reaction.

## Results and Discussion

The starting enamines **1a–e** bearing imidazole (**1a,b**) and isoxazole (**1c–e**) rings were prepared from the corresponding 5-methylazoles by reaction with dimethylformamide dimethyl acetal (DMF-DMA) adapting synthetic procedures published earlier [26–30]. Their structures can be unambiguously assigned as the *trans*-isomers by observing the coupling constant (*J* = 13.0–13.6 Hz) for the protons of the vinyl fragment in the ^1^H NMR spectra.

**Figure 2:**
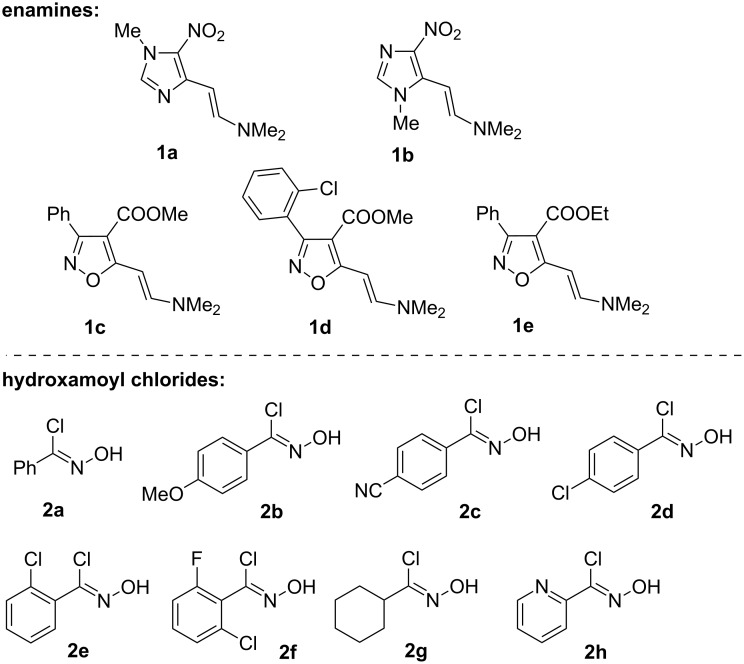
Structures of starting enamines **1** and hydroxamoyl chlorides **2**.

Hydroxamoyl chlorides are known as masked nitrile oxides and the latter could easily be generated by reaction of the former with a base [31–33]. Nitrile oxides, apart from the expected cycloaddition reaction, could undergo dimerization affording isomeric products with different structures [31–33]. Therefore we could expect, besides isoxazoles, the formation of various byproducts in the reaction of hydroxamoyl chlorides **2a–h** with β-azolyl enamines **1a–e** (Figure 2). This complicates the base-catalyzed preparation of 4-azolylisoxazoles from enamines and hydroxamoyl chlorides, lowering the yield.

**Scheme 2:**
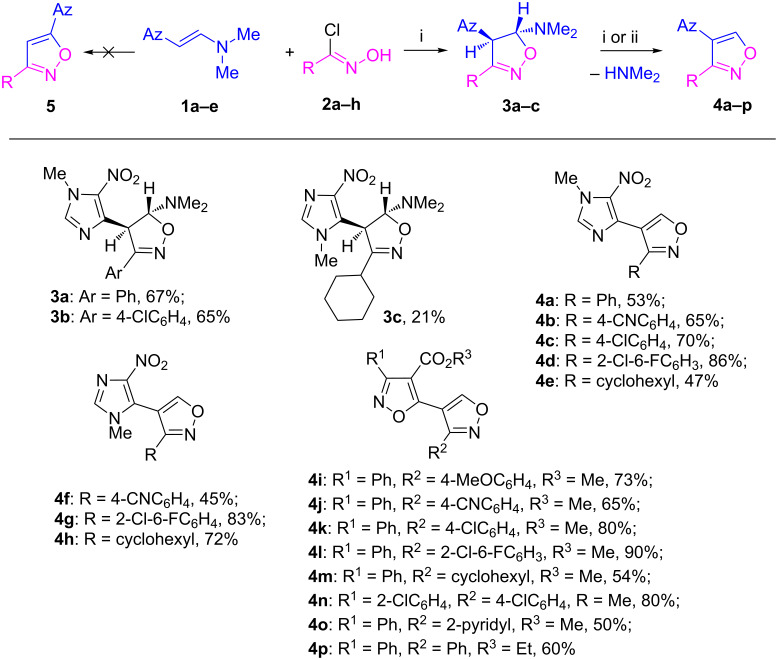
Synthesis of 4-azolylisoxazoles **4a–p** from enamines **1a–e** and hydroxamoyl chlorides **2a–h**. Reaction conditions: (i) **1** (1.1 mmol), **2** (1 mmol), 1,4-dioxane (5 mL), rt, 12–48 h, (ii) **3b,c** (1 mmol), H_2_O/HOAc (1:1 mixture) (2 mL), rt, 30 min (**3b**) or overnight (**3c**).

Fortunately, we have found that the reaction of enamines **1a–e** with hydroxamoyl chlorides **2a–h** can take place smoothly at room temperature in 1,4-dioxane solution without base to form 4-imidazolyl- and 4-isoxazolylisoxazoles **4a–p** as exclusive products in good yields (Scheme 2). Probably the formation of nitrile oxides from hydroxamoyl chlorides occurs catalytically due to formation of dimethylamine as a result of the aromatization reaction with the isoxazoles, in which dimethylamine is formed. One can also propose that enamines **1** or traces of dimethylamine can act as base and facilitate the initial formation of nitrile oxides. Thus, different conjugated heterocycles of type **4** could be prepared starting from different enamines **1a–e** and hydroxamoyl chlorides **2a–h**. The yields vary from 43 to 90% (Scheme 2, see Supporting Information File 1 for full experimental data), and are mainly in the 65−90% range.

**Figure 3:**
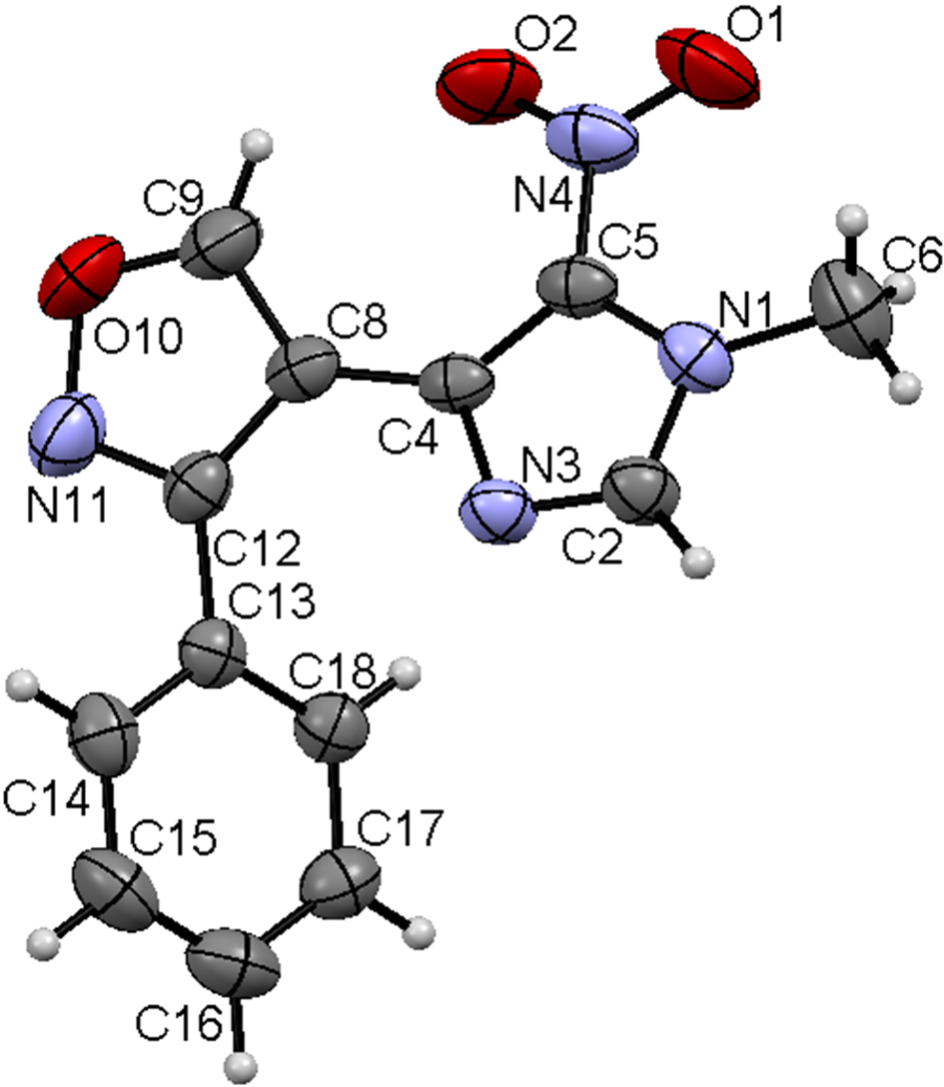
Imidazolylisoxazole **4a** according to XRD data in the thermal ellipsoids of the 50% probability level.

**Figure 4:**
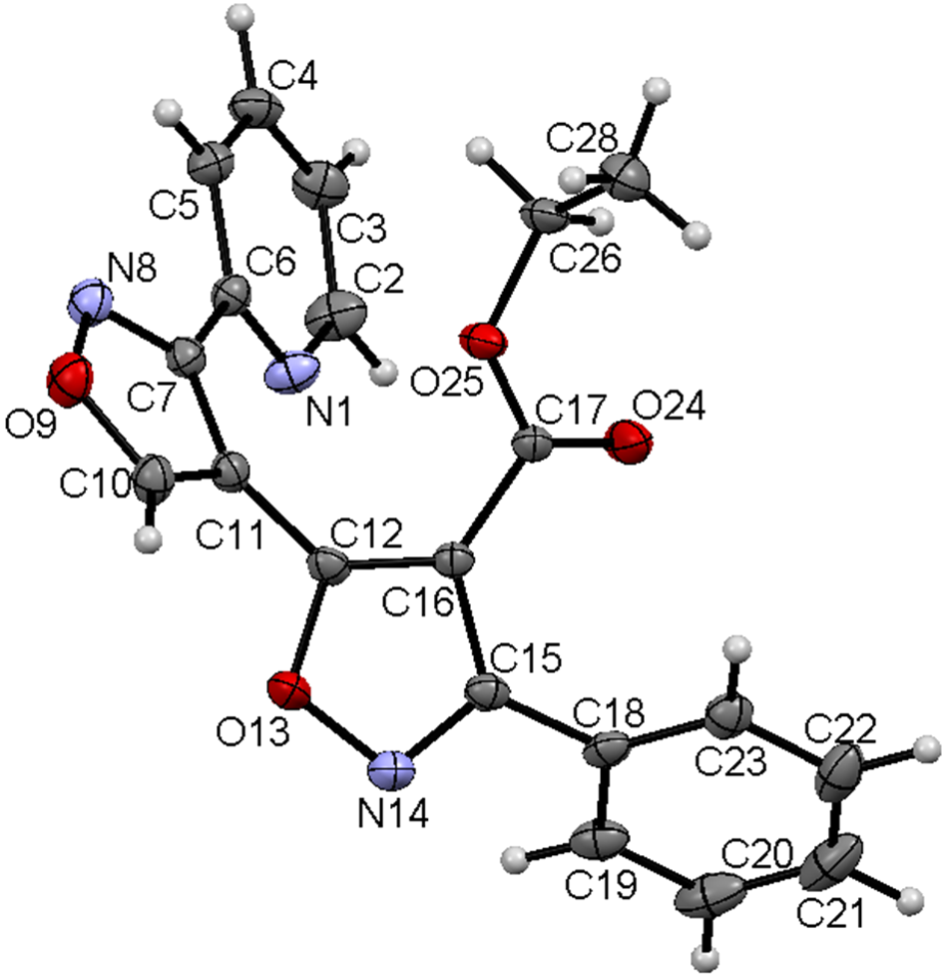
Isoxazolylisoxazole **4p** according to XRD data with thermal ellipsoids of 50% probability level.

Imidazolylisoxazoles **4a–h**, imidazolylisoxazolines **3a–c** and 3-aryl-4-carboxyisoxazol-5-ylisoxazoles **4i–p** are novel conjugated heterocycles. There are very few known examples of isoxazol-5-ylisoxazoles, that have been prepared by other methods [14–15]. In contrast to our approach, these methods are not applicable for the regioselective synthesis of compounds containing a 3-aryl-4-carboxyisoxazole fragment, a structural motif in the anticancer pyrazolylisoxazole, and rather the 3,5-disubstituted isoxazole (compound **5**, Scheme 2) is expected [12]. The presence of regioisomer **5** was not registered by TLC control and NMR spectra of the crude reaction mixture. A rise of temperature and use of other solvents decrease the product yields due to formation of tar-like products. The presence of a base increases the rate but also considerably decreases the yields of the products due to the formation of a large amount of tar-like products. Enamines **1a–e**, bearing imidazole and isoxazole rings and several hydroxamoyl chlorides **2a–h** bearing aryls with electron-withdrawing and releasing groups, pyridine and cyclohexane can be used for the synthesis of azolylisoxazoles **4a–p**. Reactions of enamines with hydroxamoyl chlorides mainly lead to aromatic isoxazoles via intermediate 4,5-dihydro-5-aminoisoxazoles, which in some cases were isolated [23–24,26]. The latter could be transformed to aromatic isoxazoles by treatment with bases or acids [32]. Reactions of β-azolyl enamines **1a–e** with hydroxamoyl chlorides **2a–h** mainly afford aromatic isoxazoles **4b,d,f–p** and intermediate isoxazolines were not detected by TLC analysis. Conversely, TLC allowed to observe the formation of imidazolylisoxazolines **3** as a result of the reaction of enamines **1a,b** with hydroxamoyl chlorides **2**. The isoxazolines transform to aromatic isoxazoles **4** during purification. Fortunately, we were able to isolate the products of the reaction of β-imidazolyl enamines **1a,b** with hydroxamoyl chlorides **2a,d,g**, and the novel imidazolylisoxazolines **3a–c** as pure stereoisomers in 67, 65 and 21% yields, respectively. To the best of our knowledge it is the first example of a stereoselective formation of 4-aryl(heteroaryl)isoxazolines in the reaction of acyclic enamines with nitrile oxides. The stereoselective formation of *trans*-4-alkylisoxazolines in the reaction of β-azolyl enamines with benzonitrile oxides was observed by Pokar et al. [34] in 1980 and diastereoselective formation of fused isoxazolines was recently reported by Jelizi et al. [35]. Their structures are assigned as *trans*-isomers which are deduced from coupling constants of 4.0 and 4.2 Hz for the C^4^–H and C^5^–H in the NMR spectra (see Supporting Information File 2 for NMR spectra descriptions and copies). In turn, isoxazolines **3b,c** were easily transformed to aromatic isoxazoles **4c,h** in aqueous acetic acid at room temperature. Interestingly, the analogous reaction of hydroxamoyl chlorides **2c,f,g** with enamines **1a,b** leads directly to aromatic 4-imidazolylisoxazoles **4b,d,e–g**. Probably isoxazolines **3a–c** are more stable than other compounds of type **3** under column purification conditions. The 3,4-disubstituted isoxazole structures of compounds **4a–p** were confirmed by the combination of mass spectrometry, NMR spectroscopy, and X-ray analysis (see Figure 3, Figure 4 and Supporting Information File 2 for details of X-ray study of compounds **4a,o,p**).

**Scheme 3:**
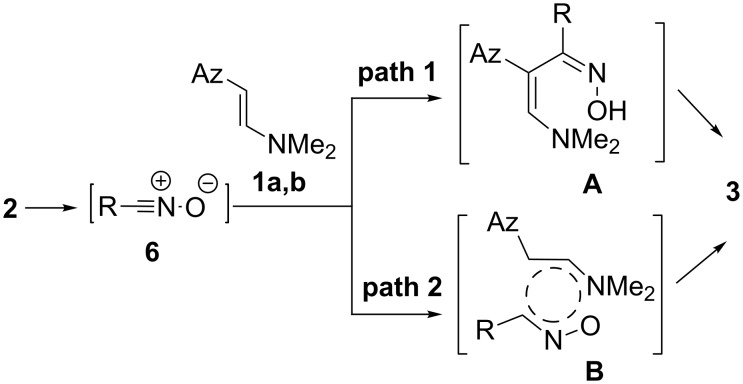
Plausible mechanisms for reaction of hydroxamoyl chlorides **2** with imidazolyl enamines **1a,b**.

According to the XRD data, the molecules of compound **4a** are non-planar with the Ph substituent turned toward the oxazole ring by a 51° angle and the imidazole ring turned toward the oxazole moiety by 23°. Crystals of **4a** possess a chiral packing, where not a shortened intermolecular contact is observable. The bond lengths and angles in molecules **4a,o,p** are standard. Apparently, hydroxamoyl chlorides transform to nitrile oxides under conditions of the isoxazole synthesis. The formation of isoxazolines **3b,c** in the reaction and their transformation to isoxazoles under mild conditions supports a mechanism where the isoxazolines are the intermediates. The exclusive formation of isoxazolines as *trans* isomers from *E*-enamines allow us to conclude that the reaction of β-azolyl enamines with hydroxamoyl chlorides proceeds in a regio- and stereospecific manner. In contrast to the reactions of these β-azolyl enamines, similar reactions of β-alkyl enamines as reported by Bujak et al. [24] are regioselective and not stereospecific and therefore not concerted. We could propose stepwise (path 1) and concerted (path 2) reaction mechanisms for the formation of isoxazolines **3a–c** as depicted in Scheme 3, in accordance with the proposed reaction mechanisms of heterocyclic enamines proposed earlier by Elliott and co-workers [36]. Path 1 includes the formation of intermediate **A** as a result of the electrophilic substitution of the β-H atom of the enamines **1a,b** after treatment with hydroxamoyl chlorides **2a,d,g**.

The intermediate **A** undergoes cyclization to oxazolines **3a–c** via addition of its hydroxy group onto the double bond of the enamine fragment. This kind of cyclization was ruled out by Elliott et al. [36] because of a disfavored 5-*endo*-trig cyclization for the corresponding intermediate oxime.

Path 2 involves the initial transformation of hydroxamoyl chloride to a nitrile oxide, followed by concerted cyclization to triazolines **3a–c**. We have found that introduction of electron-withdrawing substituents into the aryl moiety of the hydroxamoyl chloride increases the rate of reaction. Thus, the reaction times of enamine **1e** with hydroxamoyl chloride **2a** (R = Ph) as determined by TLC analysis is 30 h (product **4p**), while those for **2f** (R = 6-Cl-2-FC_6_H_3_) is 18 h (product **4l**) and for **2b** (R = 4-MeOC_6_H_4_) 32 h (products **4i**), respectively. The regio- and stereospecificity and the apparent reaction rate increase by the introduction of electron-withdrawing substituents into the benzonitrile oxide structure, allow to presume that the reaction of enamines with nitrile oxides can be described as 1,3-dipolar cycloadditions with inverse electron demand, similarly to the reaction of enamines with azides [37].

To gain deeper insights into the mechanism of the cycloaddition between nitrile oxides and enamines, quantum chemical calculations were carried out using the Gaussian 09 [38] programs package at B3LYP/def2-TZVP [39–42] theory level. Grimme’s D3BJ dispersion correction [43–44] was applied to improve the long range interactions related calculation accuracy [45]. To the best of our knowledge there is not a high level of theoretical investigations reported on the possible reaction pathways of nitrile oxides with acyclic enamines so far, but only a few works based on a semi-empirical approach and a study of Domingo on reactivity of exocyclic enamines [46–50].

**Figure 5:**
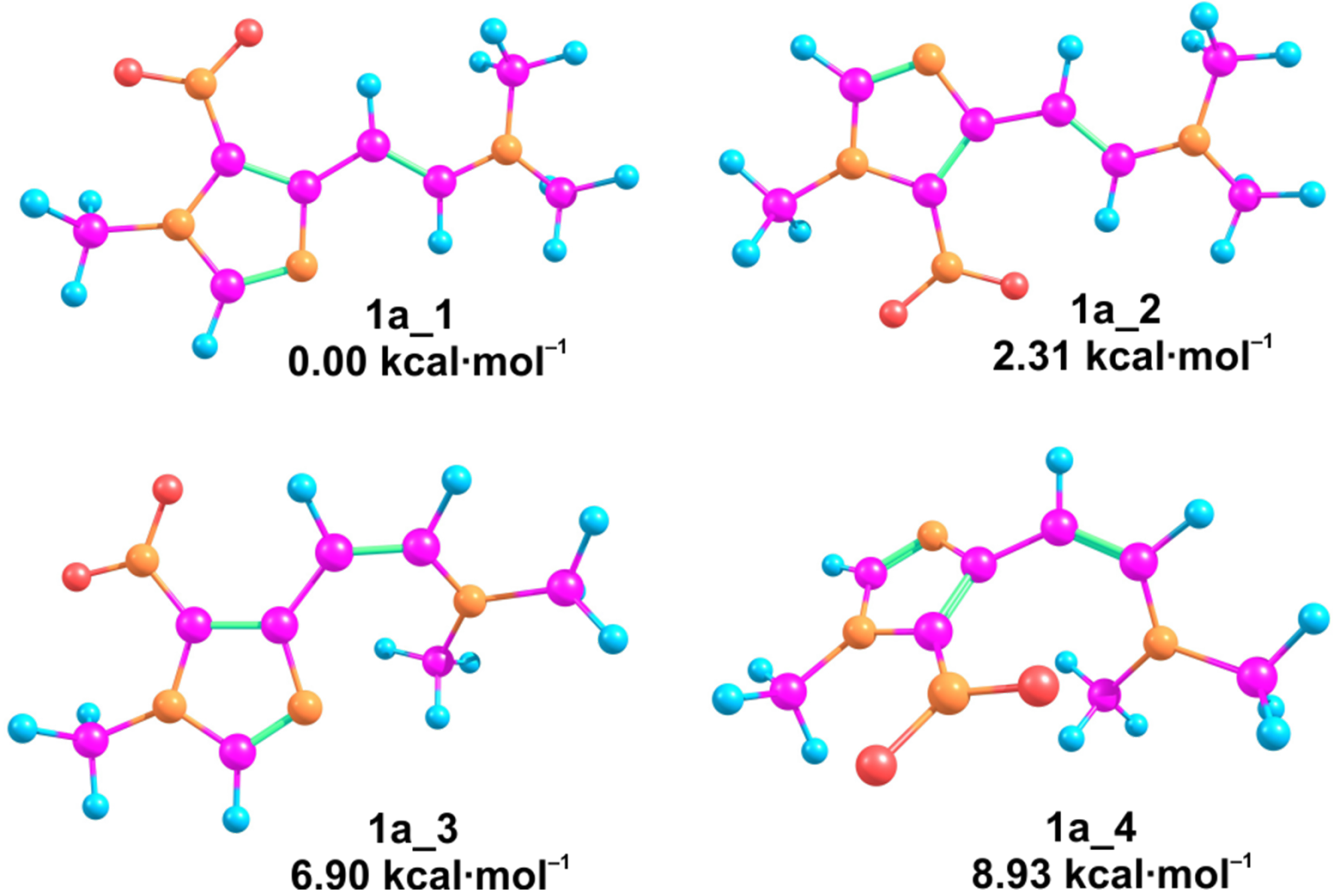
Geometries of enamine **1a** appropriate to the calculated minima on the PES, and their relative free energies at 298.15 K in gas phase conditions.

For the theoretical investigation, nitrile oxide **6a** and enamine **1a** were chosen as the model reactants. The geometries of starting molecules and products can be associated with a local minimum on the potential energy surface (PES) as proven by calculation of the vibrational frequencies among which not an imaginary value was found. Transition state geometries were proven by the presence of the only imaginary frequency appropriate to the reaction’s pathway. Geometry optimization performed on the starting enamine **1a** allowed localizing four minimums on the PES with geometries shown in Figure 5.

**Scheme 4:**
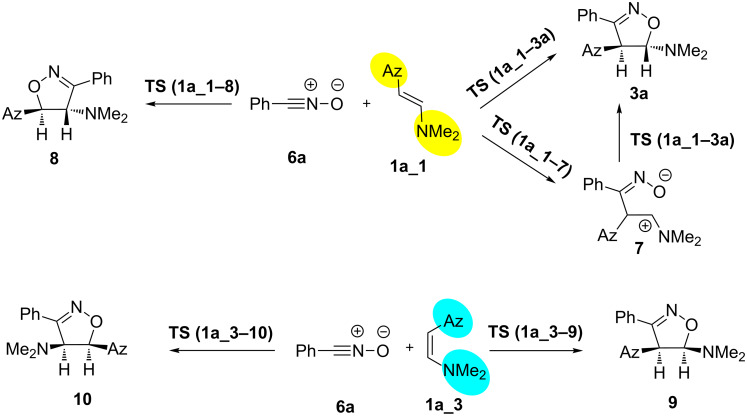
Calculated pathways for the formation of experimentally observed **3a**, regioisomer **7** and isoxazoline **8**.

The transition state between the lowest energy *E*- and *Z*-isomers of **1a**, **1a_1** and **1a_3**, respectively, was calculated by scanning the dihedral angle around the double bond, and found to be 38.09 kcal∙mol^−1^ above **1a_1** in free energy. Calculations of the suggested mechanisms (Scheme 3) performed for *E*-isomer **1a_1** and nitrile oxide **6a** allowed localizing a concerted transition state for both, observed and not observed regioisomers **3** and **8**, respectively. Investigation of the stepwise mechanism’s pathway allowed locating the only transition state (**1a_1−7**), which is appropriate to the addition of nitrile oxide **2a** onto the double bond of enamine **1a_1**, and the respective product – oxime **7**. However, the intermediate **7** was found to cyclize to isoxazoline **3a** through the transition state corresponding to the concerted mechanism, e.g., via TS **1a_1−3a** (Scheme 4). Interestingly, isoxazolines **9** and **10** with *cis* orientation of the Az and NMe_2_ groups, were found to be the products of transition states **1a_3−9** and **1a_3−10**, respectively, both based on **1a_3** geometry of enamine **1a**, whereas transition states based on **1a_1** geometry of the enamine lead to isoxazolines **3a** and **8** with *trans* orientation of the Az and NMe_2_ groups (Scheme 4). Thus, one could conclude, that the configuration around the double bond of the enamine controls the stereoconfiguration of the isoxazoline formed. Apparently, the lower stability of **1a_3** compared to **1a_1**, results in an equilibrium strongly shifted towards the latter and, consequently, in the stereoselective formation of isoxazoline **3a**.

**Figure 6:**
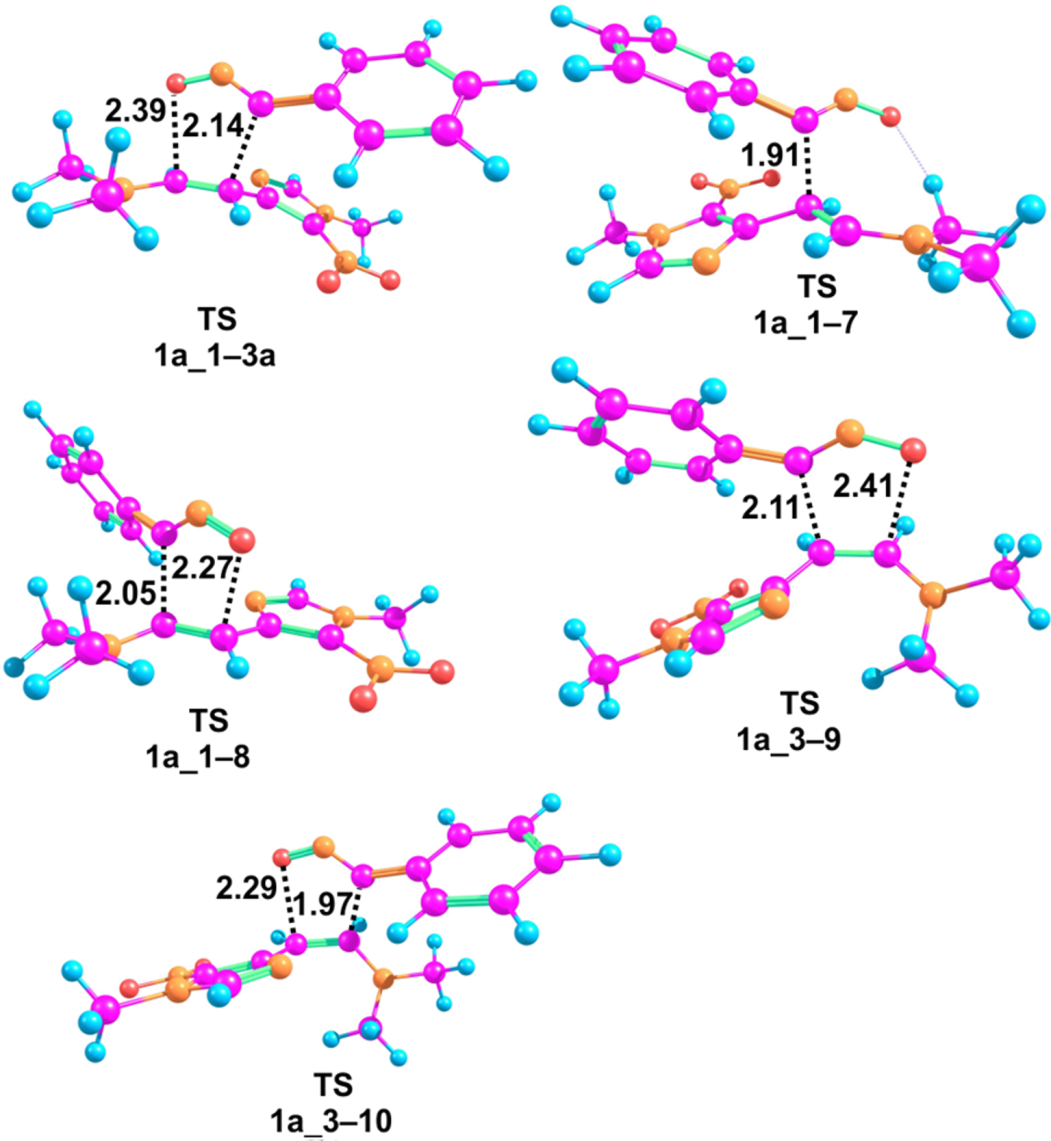
Structures of the localized transition states. Lengths of the forming bonds are given in Å.

According to the obtained transition state geometries of the cycloaddition, the concerted mechanism is quite asynchronous, which is indicated by the shorter length of the forming C–C σ-bond compared to C–O (Figure 6). A highly asynchronous transition state was also observed by the Domingo group some years ago for the reaction of exocyclic enamines with benzonitrile oxides leading to spirocyclic isoxazolines [46].

It is remarked, that transition states **1a_1−3a** and **1a_3−9** have longer lengths of the forming bonds compared to those of transition states **1a_1−8** and **1a_3–10**, which indicates an easier attainability of the formers. Calculations of the thermodynamic parameters suggest that transition state **1a_1−3a**, leading to observed product **3a**, is by 8.9 kcal∙mol^−1^ more stable than transition state **1a_1−8**, leading to the not observed regioisomer **8**, and by 5.3 kcal∙mol^−1^ more stable than state **1a_1−7** of the stepwise mechanism. Isoxazoline **3a** in its own is by 7.1 kcal∙mol^−1^ more stable than regioisomer **8**.

**Figure 7:**
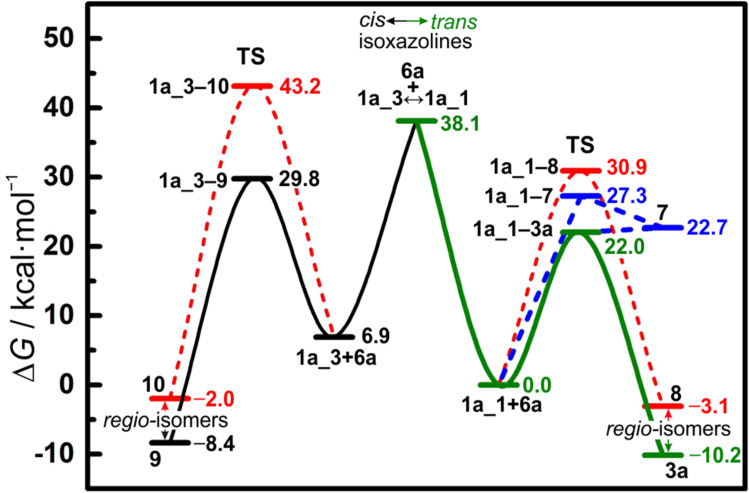
Summary of the calculated pathways of the cycloaddition reaction between enamine **1a** and benzonitrile oxide **6a**. The pathway leading to the observed product is highlighted green. Values of the free energies are given in respect to the sum of free energies of enamine **1a_1** and nitrile oxide **6a** at the optimized geometries.

These values clearly show a lower energy barrier for transition state **1a_1−3a** compared to the others considered here, which explains the observed regioselective formation of isoxazoline **3a** (Figure 7). An analysis, applying geometry strain model and molecular orbitals theory [51], reveals that the geometry distortion of nitrile oxide **6a** has a major contribution to the activation energy barrier, whereas the geometry distortion energy of the enamine is minor. Also, the analysis shows close values of the orbital interaction energy for all the found transition states (Table 1). It should be noted the close orbital interaction energy values are obtained at longer distances between the reactants in case of transition states **1a_3a** and **1a_9**, as compared to transition states **1a_8** and **1a_10**, respectively. This indicates a longer-range orbital interaction between the reactants in the case of formers, resulting in smaller geometry distortions.

Thus, the lowest electronic activation energy of transition state **1a_1−3a** among all calculated transition states is a result of both, an easier attainability of the state governed by longer range orbital interactions, and a smaller value of the total geometry distortion energy (Table 1).

**Table 1:** Calculated data of the electronic activation energy (∆*E*), orbital interaction energy (∆*E*_i_), and geometry distortion energies (∆*E*_d_).^a^

Entry	TS	∆*E*^b^	∆*E*_i_	∆*E*_d total_	∆*E*_d enamine_	∆*E*_d nitrile oxide_

1	**1a_1−3a**	8.0384	−22.8691	30.9075	7.0448	23.8627
2	**1a_1−7**	12.8883	−23.3032	36.1915	9.5132	26.6783
3	**1a_1−8**	16.9191	−22.0768	38.9959	10.4814	28.5145
4	**1a_3−9**	8.4848	−24.1853	32.6701	7.3005	25.3696
5	**1a_3−10**	22.2029	−23.0625	45.2654	13.7263	31.5391

^a^The values are given with respect to enamine **1a_1** (**1a_3** for TS **1a_3−9** and **1a_3−10**) and nitrile oxide **6a**; ^b^**∆***E* given in kcal·mol^−1^.

**Figure 8:**
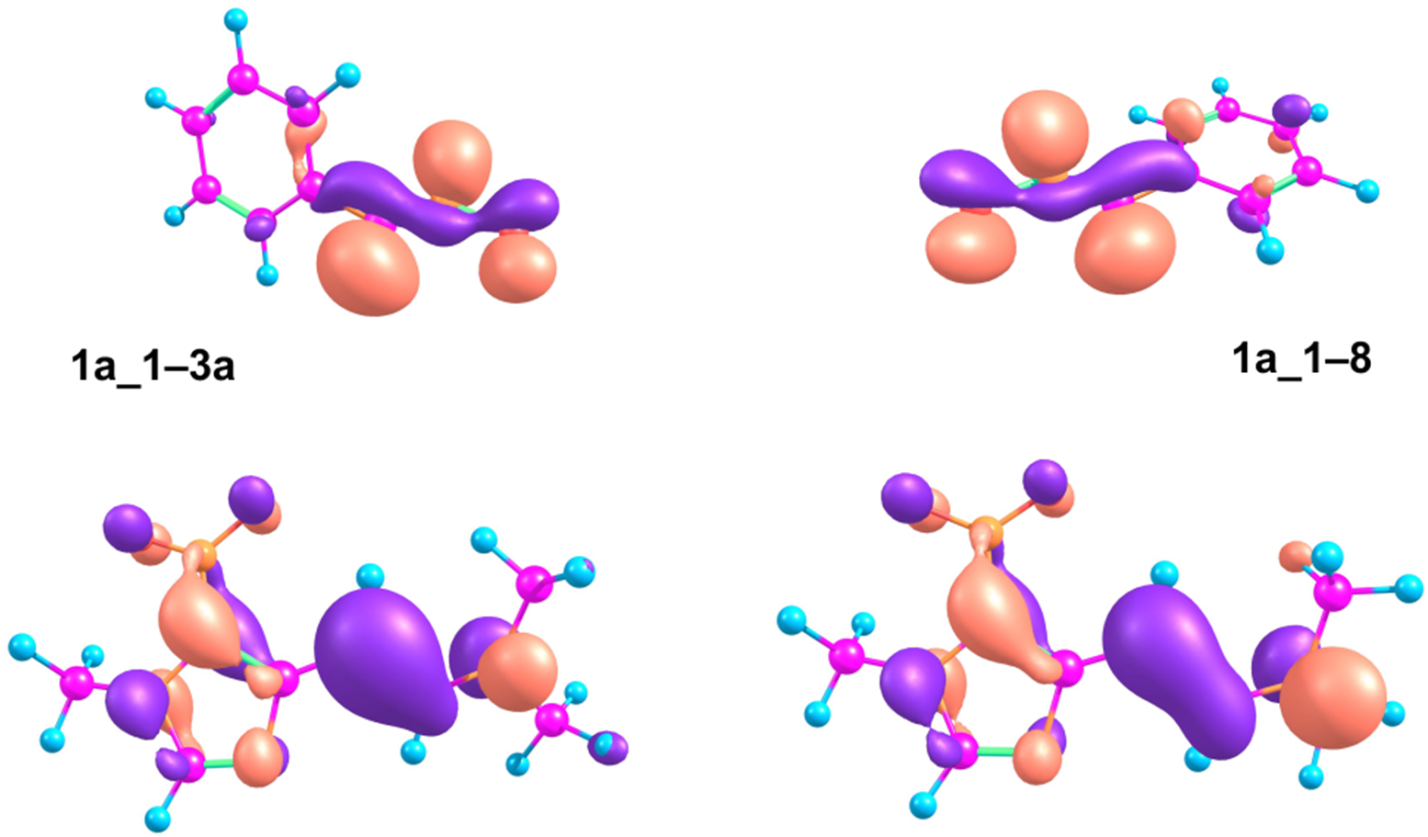
Isosurface plots of the HOMO of enamine **1a_1** (bottom) and the LUMO of nitrile oxide **6** (top) in the geometries of transition states **1a-1−3a** (left) and **1a_1−8** (right), visualized at isovalue 0.05 by the use of Chemcraft 1.8 program (http://www.chemcraftprog.com/).

Plots of respective orbitals at transition state geometries show a better orbital interaction in **1a_1−3a** transition state compared to **1a_1−8**. The HOMO of **1a_1** is predominantly localized at the β-carbon atom from the amino group whereas the LUMO of **6a** has major localization on the carbon atom of the nitrile group (Figure 8). Calculated energy gaps between HOMO of **1a_1** and LUMO of **6a** and vice versa at optimized geometries are 3.68 eV and 4.57 eV, respectively. This finding is in agreement with the inverse electron-demand concept for the investigated reaction which explains well the observed reaction rate increase when an electron-withdrawing group is introduced to the structure of nitrile oxide. Thus, according to calculations, reaction of enamines with nitrile oxides leading to isoxazolines, complies to cycloaddition with inverse electron-demand. The observed stereoselectivity of the cycloaddition is probably driven by the higher stability of the *E*-isomer of the starting enamine, whereas the regioselectivity is controlled by better orbital overlap in the transition state leading to the experimentally observed regioisomer.

## Conclusion

β-Azolyl enamines bearing imidazole and isoxazole rings were shown to react regioselectively with aryl, pyridyl and cyclohexylhydroxamoyl chlorides in the absence of a base or a catalyst to afford 4-azolylisoxazoles as the only isomers. The data of combined experimental and theoretical studies allow classifying the reaction of enamines with nitrile oxides as inverse electron-demand 1,3-dipolar cycloaddition. The found stereoselectivity of the cycloaddition is driven by the higher stability of the *E*-isomer of the starting enamine, whereas the regioselectivity is controlled by better orbital overlap in the transition state leading to the experimentally observed regioisomer. The reason for the lowest electronic activation energy leading to the experimentally observed product among all calculated transition states, is caused by both relatively better stabilizing orbital interactions and relatively smaller geometry distortion energies.

## Experimental

^1^H and ^13^C NMR spectra were recorded on Bruker Avance II spectrometer in DMSO-*d*_6_ or CDCl_3_ (400 and 100 MHz, respectively) using Me_4_Si as an internal standard. Mass experiments were performed on Shimadzu GCMS-QP2010 Ultra gas chromatograph operating at an ionization potential of 70 eV (EI). The IR data have been recorded on a Bruker Alpha (NPVO, ZnSe) FTIR spectrometer. Microanalyses were performed on PerkinElmer Series II CHNS/O 2400 elemental analyzer. The melting point was determined on a Stuart SMP 3 apparatus. The progress of the reactions and the purity of the compounds were monitored by TLC on TLC Silica gel 60 F245 Aluminum sheets (Merck KGaA) in EtOAc/hexane (1:2), EtOAc/hexane (1:1), CHCl_3_/EtOH (1:1), CHCl_3_/EtOH (20:1), CHCl_3_/EtOH (50:1) system.

### General procedure for the preparation of 4-azolylisoxazoles **4a**,**b**,**d**–**g**,**i**–**p**

To a solution of the corresponding enamine **1** (1.1 mmol) in 1,4-dioxane (5 mL) an appropriate hydroxamoyl chloride **2** (1 mmol) was added. The reaction mixture was stirred at room temperature for 12–32 h. The solvent was removed under reduced pressure and then 10 mL of C_2_H_5_OH/H_2_O (1:1) mixture was added to the oily precipitate. The formed precipitate was filtered off, washed with EtOH and purified by silica gel (60–120) column chromatography (EtOAc/hexane, CHCl_3_/EtOH, CH_2_Cl_2_) or crystallization from EtOH to afford the desired isoxazole **4**.

**4-(1-Methyl-5-nitro-1*H*-imidazol-4-yl)-3-phenylisoxazole (4a).** Colorless powder, yield 53%, mp 130–132 °С (column, CHCl_3_/EtOH 20:1); IR (ν/сm^−1^): 3097, 1611, 1472, 1329, 1134; ^1^H NMR (DMSO-*d*_6_) δ 3.95 (s, 3H, CH_3_), 7.12–7.62 (m, 5H, CH_Ar_), 8.13 (s, 1H, CH_imidaz._), 9.33 (s, 1H, C^5^−H); ^13^C NMR (DMSO-*d*_6_) δ 35.5, 111.4, 127.6, 128.3, 128.7, 129.8, 132.2, 135.9, 141.7, 160.3, 160.9; EIMS (*m/z)*: 270 [M]^+^; anal. calcd for C_13_H_10_N_4_O_3_: C, 57.78; H, 3.73; N, 20.73; found: C, 57.43; H, 3.45; N, 20.99.

**4-[4-(1-Methyl-5-nitro-1*H*-imidazol-4-yl)isoxazol-3-yl]benzonitrile (4b).** Pink powder, yield 65%, mp 189–191 °С (column, CH_2_Cl_2_); IR (ν/сm^−1^): 3109, 2227, 1615, 1480, 1354; ^1^Н NMR (CDCl_3_) δ 4.02 (s, 3H, CH_3_), 7.55 (s, 1H, CH_imidaz._), 7.67 (s, 4H, CH_Ar_), 8.99 (s, 1H, C^5^–H); ^13^С NMR (CDCl_3_) δ 36.3, 111.4, 113.6, 118.5, 129.3, 132.3, 133.0, 133.6, 135.9, 140.5, 160.0, 160.8; EIMS (*m/z*): 295 [M]^+^; anal. calcd for C_14_H_9_N_5_O_3_: C, 56.95; H, 3.07; N, 23.72; found: C, 56.85; H, 3.01; N, 23.79.

### Preparation of isoxazolines **3a–c**

Isoxazolines **3a–c** were synthesized in the same manner as isoxazoles **4a,b,d–g,i–p** (see general procedure). The reaction time is 12 h (**3a,b**) and 48 h (**3c**).

***N*,*N*-Dimethyl-4-(1-methyl-5-nitro-1*H*-imidazol-4-yl)-3-phenyl-4,5-dihydroisoxazol-5-amine (3a).** Colorless powder, yield 67%, mp 132–134 °С (column, EtOAc/hexane 1:1); ^1^H NMR (DMSO-*d*_6_) δ 2.34 (s, 6H, NMe_2_), 3.91 (s, 3H, NCH_3_), 5.28 (d, *J* = 4.2 Hz, 1Н, СН), 5.30 (d, *J* = 4.2 Hz, 1Н, СН), 7.29–7.33 (m, 3H, H_Ar_), 7.49–7.58 (m, 3H, H_Ar_), 7.89 (s, 1H, CH_imidaz._); ^13^С NMR (DMSO-*d*_6_) δ 35.9, 39.3, 49.1, 104.2, 126.8, 128.1, 129.3, 130.2, 135.5, 142.2, 142.7, 155.2; EIMS (*m/z*): 315 [M]^+^; anal. calcd for C_15_H_17_N_5_O_3_: C, 57.13; H, 5.43; N, 22.21.; found: C, 57.07; H, 5.66; N, 22.10.

**3-(4-Chlorophenyl)-*N*,*N*-dimethyl-4-(1-methyl-5-nitro-1*H*-imidazol-4-yl)-4,5-dihydroisoxazol-5-amine (3b).** Colorless powder, yield 65%, mp 164–166 °С (EtOH); IR (ν/сm^−1^): 3120, 1490, 1361, 1306; ^1^Н NMR (DMSO-*d*_6_) δ 2.34 (s, 6H, NMe_2_), 3.92 (s, 3H, СН_3_), 5.29 (d, *J* = 4.0 Hz, 1Н, СН), 5.32 (d, *J* = 4.0 Hz, 1Н, СН), 7.31 (d, *J* = 8.8 Hz, 2H, H_Ar_), 7.54 (d, *J* = 8.8 Hz, 2 H, H_Ar_), 7.89 (s, 1H, CH_imidaz._); ^13^С NMR (DMSO-*d*_6_) δ 35.9, 39.3, 49.0, 104.6, 128.3, 128.6, 129.4, 134.8, 135.5, 142.2, 142.4, 154.4; EIMS (*m/z*): 349 [M]^+^; anal. calcd for C_15_H_16_ClN_5_O_3_: C, 51.51; H, 4.61; N, 20.02; found: C, 51.65; H, 4.47; N, 20.28.

### Transformation of isoxazolines **3b**,**c** to isoxazoles **4c**,**h**

A solution of corresponding isoxazoline **3b,c** (1 mmol) in a mixture of H_2_O/HOAc (1:1, 2 mL) was stirred at room temperature for 30 minutes (**3b**) or overnight (**3c**). The formed precipitate was filtered off, washed with H_2_O and dried in a desiccator over P_2_O_5_ and recrystallized from EtOH (**4c**) or purified by flash column chromatography (CH_2_Cl_2_) (**4h**).

**3-(4-Chlorophenyl)-4-(1-methyl-5-nitro-1*H*-imidazol-4-yl)isoxazole (4c).** Pink powder, yield 70%, mp 129–131 °С (EtOH); ^1^Н NMR (DMSO-*d*_6_) δ 3.95 (s, 3H, СН_3_), 7.43–7.56 (m, 4H, H_Ar_), 8.13 (s, 1H, CH_imidaz._), 9.37 (s, 1H, C^5^–H); ^13^С NMR (DMSO-*d*_6_) δ 35.5, 111.4, 127.2, 128.8, 129.5, 131.6, 134.7, 135.9, 141.8, 159.4, 161.3; EIMS (*m/z*): 304 [M]^+^; anal. calcd for C_13_H_9_ClN_4_O_3_: C, 51.25; H, 2.98; N, 18.39; found: C, 51.36; H, 2.79; N, 18.52.

### X-ray diffraction study

X-ray analyses were accomplished on an Xcalibur 3 diffractometer using the standard procedure (graphite-monochromated Mo K-irradiation, ω-scanning with step 1^o^, T = 150.00(10) K) (**4o,p**) or 295(2) K (**4a**) (See Supporting Information File 2). Using Olex2 [52], the structures were solved with the Superflip [53] structure solution program using Charge Flipping and refined with the ShelXL [54] refinement package using Least Squares minimization. Deposition numbers for compounds **4a** (1473400), **4o** (1405543) and **4p** (1405542) contain the supplementary crystallographic data for this paper. These data can be obtained free of charge from the Cambridge Crystallographic Data Centre via http://www.ccdc.cam.ac.uk/data_request/cif.

## Supporting Information

Supporting information features copies of ^1^H and ^13^C NMR spectra for all compounds synthesized and full experimental and analytical data for isoxazolines **3** and isoxazoles **4**.

File 1 Experimental part.

File 2 NMR spectra of compounds **3a–c**, **4a–p** and X-ray study of isoxazoles **4a,o,p**.

File 3 Crystallographic information file for compound **4a**.

File 4 Crystallographic information file for compound **4o**.

File 5 Crystallographic information file for compound **4p**.

## References

[1] Giomi D, Cordero F M, Machetti F, Katritzky A R, Rees C W, Scriven E F V (2008). Isoxazoles. Comprehensive Heterocyclic Chemistry III.

[2] Gehling V S, Hewitt M C, Vaswani R G, Leblanc Y, Côté A, Nasveschuk C G, Taylor A M, Harmange J-C, Audia J E, Pardo E (2013). ACS Med Chem Lett.

[3] Di Nunno L, Vitale P, Scilimati A, Tacconelli S, Patrignani P (2004). J Med Chem.

[4] Girardin M, Dolman S J, Lauzon S, Ouellet S G, Hughes G, Fernandez P, Zhou G, O’Shea P D (2011). Org Process Res Dev.

[5] Brodney M A, Beck E M, Butler C R, Barreiro G, Johnson E F, Riddell D, Parris K, Nolan C E, Fan Y, Atchison K (2015). J Med Chem.

[6] Galenko A V, Khlebnikov A F, Novikov M S, Pakalnis V V, Rostovskii N V (2015). Russ Chem Rev.

[7] Bakulev V A, Tarasov E V, Morzherin Y Y, Luyten I, Toppet S, Dehaen W (1998). Tetrahedron.

[8] Bakulev V A, Dehaen W (2004). The Chemistry of 1,2,3-Thiadiazoles.

[9] Bakulev V A, Mokrushin V S (1986). Khim Geterotsikl Soedin.

[10] Shafran Y M, Bakulev V A, Mokrushin V S, Alexeev S G (1984). Khim Geterotsikl Soedin.

[11] Bakulev V, Dehaen W, Beryozkina T, Dehaen W, Bakulev V (2015). Thermal Rearrangements and Transformations of 1,2,3-Triazoles. Chemistry of 1,2,3-triazoles.

[12] Leban J, Tasler S, Saeb W, Chevrier C (2012). IL17 And IFN-gamma inhibition for the treatment of autoimmune inflammation. Canadian Patent.

[13] Buettelmann B, Han B, Knust H, Thomas A (2007). Aryl-isoxazol-4-yl-imidazole derivatives. WO Patent.

[14] Thorarensen A, Ruble C J, Fisher J F, Romero D L, Beauchamp T J, Northuis J M (2004). Antibacterial benzoic acid derivatives. WO Pantent.

[15] Eiden F, Patzelt G (1986). Arch Pharm.

[16] Lautens M, Roy A (2000). Org Lett.

[17] Himo F, Lovell T, Hilgraf R, Rostovtsev V V, Noodleman L, Sharpless K B, Fokin V V (2005). J Am Chem Soc.

[18] Jones R C F, Bhalay G, Carter P A (1993). J Chem Soc, Perkin Trans 1.

[19] Jones R C F, Dunn S H, Duller K A M (1996). J Chem Soc, Perkin Trans 1.

[20] Jones R C F, Bhalay G, Carter P A, Duller K A M, Dunn S H (1999). J Chem Soc, Perkin Trans 1.

[21] Jones R C F, Duller K A M (2002). ARKIVOC.

[22] Sasaki T, Yoshioka T (1968). Bull Chem Soc Jpn.

[23] Krompiec S, Bujak P, Szczepankiewicz W (2008). Tetrahedron Lett.

[24] Bujak P, Krompiec S, Malarz J, Krompiec M, Filapek M, Danikiewicz W, Kania M, Gębarowska K, Grudzka I (2010). Tetrahedron.

[25] Gong Y, Wang Y, Zhao W-T, Tang X-Y (2013). J Chem Res.

[26] Bakulev V A, Efimov I V, Belyaev N A, Zhidovinov S S, Rozin Yu A, Volkova N N, Khabarova A A, El'tsov O S (2013). Chem Heterocycl Compd.

[27] Bakulev V A, Efimov I V, Belyaev N A, Rozin Y A, Volkova N N, El’tsov O S (2012). Chem Heterocycl Compd.

[28] Beryozkina T V, Zhidovinov S S, Shafran Y M, Eltsov O S, Slepukhin P A, Leban J, Marquez J, Bakulev V A (2014). Tetrahedron.

[29] Shafran Y, Rozin Y, Beryozkina T, Zhidovinov S, Eltsov O, Subbotina J, Leban J, Novikova R, Bakulev V (2012). Org Biomol Chem.

[30] Hosmane R S, Bhan A, Rauser M E (1985). J Org Chem.

[31] Jäger V, Colinas P A, Padwa A, Pearson W H (2002). Nitrile Oxides. Synthetic Applications of 1,3-Dipolar Cycloaddition Chemistry Toward Heterocycles and Natural Products.

[32] Caramella P, Grunanger P, Padwa A (1984). 1,3-Dipolar Cycloaddition Chemistry.

[33] Grundmann C, Grünanger P (1971). The Nitrile Oxides.

[34] Pokar D, Rossi L M, Trimarco P, Vago L (1980). J Heterocycl Chem.

[35] Jelizi H, Wannassi N, Rammah M E B, Ciamala K, Knorr M, Rousselin Y, Kubicki M M, Strohmann C, Enescu M (2014). J Heterocycl Chem.

[36] Altuğ C, Dürüst Y, Elliott M C, Kariuki B M, Rorstad T, Zaal M (2010). Org Biomol Chem.

[37] Lopez A S, Munk M E, Houk K N (2013). J Org Chem.

[38] (2009). Gaussian 09.

[39] Weigend F, Ahlrichs R (2005). Phys Chem Chem Phys.

[40] Weigend F (2006). Phys Chem Chem Phys.

[41] Becke A D (1993). J Chem Phys.

[42] Becke A D (1993). J Chem Phys.

[43] Grimme S, Antony J, Ehrlich S, Krieg H (2010). J Chem Phys.

[44] Grimme S, Ehrlich S, Goerigk L (2011). J Comput Chem.

[45] Armstrong A, Boto R A, Dingwall P, Contreras-García J, Harvey M J, Mason N J, Rzepa H S (2014). Chem Sci.

[46] Houk K N, Sims J, Duke R E, Strozier R W, George J K (1973). J Am Chem Soc.

[47] Caramella P, Corsico A C, Corsaro A, Del Monto D, Albini F M (1982). Tetrahedron.

[48] Tsoleridis C A, Dimtsas J, Hatzimimikou D, Stephanidou-Stephanatou J (2006). Tetrahedron.

[49] Domingo L R, Picher M T, Arroyo P, Saez J A (2006). J Org Chem.

[50] Ess D H, Houk K N (2007). J Am Chem Soc.

[51] Fernández I, Bickelhaupt F M (2014). Chem Soc Rev.

[52] Dolomanov O V, Bourhis L J, Gildea R J, Howard J A K, Puschmann H (2009). J Appl Crystallogr.

[53] Palatinus L, Chapuis G (2007). J Appl Crystallogr.

[54] Sheldrick G M (2008). Acta Crystallogr, Sect A.

