# Supplementary material for: Combined experimental and theoretical studies of regio- and stereoselectivity in reactions of β-isoxazolyl- and β-imidazolyl enamines with nitrile oxides
Source: Beilstein J Org Chem. 2016 Nov 15;12:2390–401. doi: 10.3762/bjoc.12.233 (PMC5238569; doi:10.3762/bjoc.12.233)
Supplement: File 1 — Experimental part. [file Beilstein_J_Org_Chem-12-2390-s001.pdf]

Supporting Information

for

Combined experimental and theoretical studies of regio- and stereoselectivity in reactions of β-isoxazolyl- and β-imidazolyl enamines with nitrile oxides

Ilya V. Efimov[1], Marsel Z. Shafikov[1,2], Nikolai A. Beliaev[1], Natalia N. Volkova[1], Tetyana V. Beryozkina[1], Wim Dehaen[3], Zhijin Fan*[4], Viktoria V. Grishko[5], Gert Lubec[6], Pavel A. Slepukhin[1,7] and Vasiliy A. Bakulev*[1]

Address: [1]TOS Department, Ural Federal University named after the first President of Russia B.N. Yeltsin, 19 Mira str., 620002 Yekaterinburg, Russia, [2]Chemistry Department, University of York, Heslington, York, YO10 5DD (UK), [3]Molecular Design and Synthesis, Department of Chemistry, KU Leuven, Celestijnenlaan 200F, 3001 Leuven, Belgium, [4]State Key Laboratory of Elemento-Organic Chemistry Nankai University, Collaborative Innovation Center of Chemical Science and Engineering 300071 Tianjin, China, [5]Laboratory of Biologically Active Compounds, Institute of Technical Chemistry Ural Branch of Russian Academy of Sciences, 3 Academician Korolev str., Perm, Russia, [6]Medical University of Vienna, Department of Pediatrics, 14 Lazarettgasse, 1090 Vienna, Austria and [7]I. Ya. Postovsky Institute of Organic Synthesis, Ural Branch of Russian Academy of Sciences, 20 S. Kovalevskaya str., 620990 Yekaterinburg, Russia

Email: Vasiliy A. Bakulev - v.a.bakulev@urfu.ru; Zhijin Fan - fanj@nankai.edu.cn

*Corresponding author

Experimental part

Contens

Full experimental and analytical data for isoxazoles **4d–g,i–p** **S2**

Experimental and analytical data for isoxazoline **3** **S5**

Experimental and analytical data for isoxazole **4h** **S5**

General procedure for the preparation of 4-azolyl isoxazoles 4d–g,i–p.

To a solution of the corresponding enamine **1** (1.1 mmol) in 1,4-dioxane (5 mL) an appropriate hydroxamoyl chloride **2** (1 mmol) was added. The reaction mixture was stirred at room temperature for 12–32 h. The solvent was removed under reduced pressure and then 10 mL of a C_2H_5OH/H_2O (1:1) mixture was added to the oily precipitate. The formed precipitate was filtered off, washed with EtOH and purified by silica gel (60–120) column chromatography (EtOAc/hexane, $CHCl_3$/EtOH, CH_2Cl_2) or crystallization from EtOH to afford the desired isoxazole **4**.

3-(2-Chloro-6-fluorophenyl)-4-(1-methyl-5-nitro-1*H*-imidazol-4-yl)isoxazole (4d). Red powder, yield 86%, mp 170–172 °C (column, $CHCl_3$/EtOH 20:1). IR (ν/cm^{-1}): 3154, 3113, 1610, 1497, 1354, 1125. ^{1}H NMR ($CDCl_3$): *δ* 3.96 (s, 3 H, CH_3), 7.07 (m, 1 H, CH_{Ar}), 7.20–7.30 (m, 1 H, CH_{Ar}), 7.31–7.43 (m, 2 H, CH_{Ar} + $CH_{imidaz.}$), 9.38 (s, 1 H, C^5–H). ^{13}C NMR ($CDCl_3$): 36.0, 112.9, 114.1, 118.1, 125.16, 131.3, 133.8, 135.0, 135.2, 140.5, 155.0, 160.2, 160.9 (*J* 250 Hz). MS-EI, (*m/z*): 322 $[M]^+$. Found: C, 48.74; H, 2.19; N, 17.57. $C_{13}H_8ClFN_4O_3$ requires: C, 48.39; H, 2.50; N, 17.36%.

3-Cyclohexyl-4-(1-methyl-5-nitro-1*H*-imidazol-4-yl)isoxazole (4e). Pink powder, yield 47%, mp 82–84 °C (column, CH_2Cl_2). IR (ν/cm^{-1}): 3111, 2931, 2854, 1613, 1486, 1357. ^{1}H NMR (DMSO-d_6): *δ* 1.11–1.34 (m, 3 H, $CH_{cyclohex.}$), 1.34–1.54 (m, 2 H, $CH_{cyclohex.}$), 1.57–1.81 (m, 3 H, $CH_{cyclohex.}$), 1.89 (d, *J* 12.3 Hz, 2 H, $CH_{cyclohex.}$), 3.03–3.21 (m, 1 H, $CH_{cyclohex.}$), 3.95 (s, 3 H, CH_3), 8.16 (s, 1 H, $CH_{cyclohex.}$), 9.22 (s, 1 H, H–5). ^{13}C NMR (DMSO-d_6): 25.5, 25.7, 31.1, 34.8, 35.6, 110.5, 133.0, 135.4, 141.9, 159.8, 165.5. MS-EI (*m/z*): 276 $[M]^+$. Found: C, 56.59; H, 5.75; N, 20.22. $C_{13}H_{16}N_4O_3$ requires: C, 56.51; H, 5.84; N, 20.28%.

4-[4-(1-Methyl-4-nitro-1*H*-imidazol-5-yl)isoxazol-3-yl]benzonitrile (4f). Pink powder, yield 45%, mp 221–223 °C (column, $CHCl_3$/EtOH 50:1). IR (ν/cm^{-1}): 3125, 2225, 1536, 1494, 1331. ^{1}H NMR (DMSO-d_6): *δ* 3.61 (s, 3 H, CH_3), 7.59 (d, *J* 8.5 Hz, 2 H, H_{Ar}), 7.90 (d, *J* 8.5 Hz, 2 H, H_{Ar}), 8.09 (s, 1 H, $CH_{imidaz.}$), 9.52 (s, 1 H, C^5–H). ^{13}C NMR (DMSO-d_6): *δ* 32.9, 105.8, 112.9, 118.1, 120.6, 127.8, 132.2, 133.0, 138.5, 144.8, 159.4, 162.0. MS-EI (*m/z*): 295 $[M]^+$. Found: C, 56.89; H, 3.12; N, 23.81. $C_{14}H_9N_5O_3$ requires: C, 56.95; H, 3.07; N, 23.72%.

3-(2-Chloro-6-fluorophenyl)-4-(1-methyl-4-nitro-1*H*-imidazol-5-yl)isoxazole (4g). Colorless powder, yield 83%, mp 210–213 °C (column, $CHCl_3$/EtOH 20:1). IR (ν/cm^{-1}): 3121, 3097, 1607, 1572, 1330, 1120. ^{1}H NMR (DMSO-d_6): δ 3.64 (s, 3 H, CH_3), 7.30–7.45 (m, 2 H, CH_{Ar}), 7.56 (m, 1 H, CH_{Ar}), 8.01 (s, 1 H, $CH_{imidaz.}$), 9.66 (s, 1 H, C^5–H). ^{13}C NMR (DMSO-d_6): δ 32.7, 108.0, 115.1, 115.3, 120.4, 125.9, 133.1, 133.2, 138.3, 144.5, 154.6, 159.8, 161.2. MS-EI (*m/z*): 287 [M-Cl]$^+$. Found: C, 48.72; H, 2.19; N, 17.57. $C_{13}H_8ClFN_4O_3$ requires: C, 48.39; H, 2.50; N, 17.36.

Methyl 3-(4-methoxyphenyl)-3'-phenyl-4,5'-bi-isoxazole-4'-carboxylate (4i). Colorless powder, yield 73%, mp 115–116 °C (column, EtOAc/hexane 1:2). IR (ν/cm^{-1}): 3124, 1720, 1609, 1524, 1254, 1120. ^{1}H NMR (DMSO-d_6): δ 3.48 (s, 3 H, OCH_3), 3.80 (s, 3 H, OCH_3), 7.02–7.07 (m, 2 H, H_{Ar}), 7.42–7.60 (m, 5 H, H_{Ar}), 7.63–7.68 (m, 2 H, H_{Ar}), 9.70 (s, 1 H, C^5–H). ^{13}C NMR (DMSO-d_6): δ 52.4, 55.8, 106.9, 110.6, 115.0, 119.8, 127.8, 128.8, 129.5, 129.8, 130.7, 159.8, 161.1, 161.3, 162.8, 163.4, 164.5. MS-EI (*m/z*): 376 [M]$^+$. Found: C, 67.13; H, 4.44; N, 7.32. $C_{21}H_{16}N_2O_5$ requires: C, 67.02; H, 4.28; N, 7.44%.

Methyl 3-(4-cyanophenyl)-3'-phenyl-4,5'-bi-isoxazole-4'-carboxylate (4j). Colorless powder, yield 65%, mp 174–175 °C (column, EtOAc/hexane 1:2). IR (ν/cm^{-1}): 3116, 2227, 1715, 1633, 1125. ^{1}H NMR (DMSO-d_6): δ 3.55 (s, 3 H, OCH_3), 7.44–7.56 (m, 3 H, H_{Ar}), 7.60–7.65 (m, 2 H, H_{Ar}), 7.74 (d, *J* 8.5 Hz, 2 H, H_{Ar}), 7.89 (d, *J* 8.5 Hz, 2 H, H_{Ar}), 9.72 (s, 1 H, C^5–H). ^{13}C NMR (DMSO-d_6): δ 52.4, 107.4, 110.5, 113.6, 118.7, 127.7, 128.8, 129.4, 129.6, 130.7, 132.4, 133.4, 159.2, 161.0, 162.9, 163.7, 164.0. MS-EI (*m/z*): 371 [M]$^+$. Found: C, 67.98; H, 3.47; N, 11.38. $C_{21}H_{13}N_3O_4$ requires: C, 67.92; H, 3.53; N, 11.32%.

Methyl 3-(4-chlorophenyl)-3'-phenyl-4,5'-bi-isoxazole-4'-carboxylate (4k). Colorless powder, yield 80%, mp 135–136 °C (column, EtOAc/hexane 1:2). IR (ν/cm^{-1}): 3134, 1706, 1620, 1556, 1142. ^{1}H NMR (DMSO-d_6): δ 3.55 (s, 3 H, OCH_3), 7.43–7.58 (m, 7 H, H_{Ar}), 7.60–7.66 (m, 2 H, H_{Ar}), 9.62 (s, 1 H, C^5–H). ^{13}C NMR (DMSO-d_6): δ 52.4, 107.2, 110.5, 126.6, 127.7, 128.8, 129.5, 129.6, 130.3, 130.7, 135.8, 159.4, 161.1, 162.8, 163.7, 164.0. MS-EI (*m/z*): 380 [M]$^+$. Found: C, 63.02; H, 3.37; N, 7.51. $C_{20}H_{13}ClN_2O_4$ requires: C, 63.08; H, 3.44; N, 7.36%.

Methyl 3-(2-chloro-6-fluorophenyl)-3'-phenyl-4,5'-bi-isoxazole-4'-carboxylate (4l). Colorless powder, yield 90%, mp 101–102 °C (column, EtOAc/hexane 1:1). IR (ν/cm^{-1}): 3150, 2923, 1707, 1610, 1567, 1130. ^{1}H NMR (DMSO-d_6): δ 3.66 (s, 3 H, OCH_3), 7.43–7.63 (m, 8 H, H_{Ar}), 7.70–7.73 (m, 1 H, H_{Ar}), 10.04 (s, 1 H, C^5–H). ^{13}C NMR (DMSO-d_6): δ 52.7, 109.0, 109.4, 115.6, 116.0, 126.5, 127.7, 128.8, 129.5, 130.7, 134.0, 134.3, 153.5, 159.4, 161.3, 161.9, 162.8, 163.4. MS-EI (*m/z*): 398 [M]$^+$. Found: C, 60.13; H, 3.15; N, 7.11. $C_{20}H_{12}ClFN_2O_4$ requires: C, 60.24; H, 3.03; N, 7.02%.

Methyl 3-cyclohexyl-3'-phenyl-4,5'-bi-isoxazole-4'-carboxylate (4m). Colorless powder, yield 54%, mp 65–67 °C (column, EtOAc/hexane 1:1). IR (ν/cm^{-1}): 2936, 2857, 1721, 1327, 1121. ^{1}H NMR (DMSO-d_6): δ 1.22–1.50 (m, 4 H, $CH_{cyclohex.}$), 1.52–1.63 (m, 2 H, $CH_{cyclohex.}$), 1.74–1.77 (m, 1 H, $CH_{cyclohex.}$), 1.81–1.94 (m, 1 H, $CH_{cyclohex.}$), 2.01 (m, 2 H, $CH_{cyclohex.}$), 3.04–3.15 (m, 1 H, $CH_{cyclohex.}$), 3.72 (s, 3H, OCH_3), 7.43–7.54 (m, 3 H, H_{Ar}), 7.60 (m, 2 H, H_{Ar}), 9.51 (s, 1 H, C^5–H). ^{13}C NMR (DMSO-d_6): δ 25.4, 25.6, 31.2, 35.4, 52.1, 106.3, 109.1, 127.5, 128.3, 129.0, 130.1, 161.0, 162.2, 162.3, 164.3, 164.5. MS-EI (*m/z*): 352 [M]$^+$. Found: C, 68.25; H, 5.79; N, 7.85. $C_{20}H_{20}N_2O_4$ requires: C, 68.17; H, 5.72; N, 7.95%.

Methyl 3'-(2-chlorophenyl)-3-(4-chlorophenyl)-4,5'-bi-isoxazole-4'-carboxylate (4n). Colorless powder, yield 80%, mp 101–102 °C (column, EtOAc/hexane 1:1). ^{1}H NMR (DMSO-d_6): δ 3.43 (s, 3 H, OCH_3), 7.43–7.72 (m, 8 H, H_{Ar}), 9.85 (s, 1 H, C^5–H). ^{13}C NMR (DMSO-d_6): δ 52.4, 106.9, 111.6, 126.7, 127.5, 127.8, 129.6, 129.9, 130.3, 131.8, 132.3, 133.2, 135.8, 159.5, 160.6, 161.4, 163.6, 163.9. MS-EI (*m/z*): 414 [M]$^+$. Found: C, 57.79; H, 2.81; N, 6.89. $C_{20}H_{12}Cl_2N_2O_4$ requires: C, 57.85; H, 2.91; N, 6.75%.

Methyl 3'-phenyl-3-(pyridin-2-yl)-4,5'-bi-isoxazole-4'-carboxylate (4o). Colorless powder, yield 50%, mp 147–149 °C (column, EtOAc/hexane 1:1). IR (ν/cm^{-1}): 3128, 1716, 1647, 1447, 1384, 1126. ^{1}H NMR (DMSO-d_6): δ 3.24 (s, 3 H, OCH_3), 7.50–7.56 (m, 4 H, H_{Ar}), 7.67–7.69 (m, 2 H, H_{Ar}), 8.00–8.05 (m, 2 H, H_{Ar}), 8.51–8.52 (m, 1 H, H_{Ar}), 9.81 (s, 1 H, C^5–H). ^{13}C NMR (DMSO-d_6): δ 51.3, 107.3, 110.2, 122.6, 125.2, 127.4, 128.4, 129.0, 130.2, 137.7, 147.0, 149.7, 159.0, 160.8, 162.1, 163.0, 164.5. MS-EI (*m/z*): 347 [M]$^+$. Found: C, 66.07; H, 4.11; N, 12.31. $C_{19}H_{13}N_3O_4$ requires: C, 65.70; H, 3.77; N, 12.10%.

Ethyl 3,3'-diphenyl-4,5'-bi-isoxazole-4'-carboxylate (4p). Colorless powder, yield 60%, mp 104–105 °C (column, EtOAc/hexane 1:1). IR (ν/cm^{-1}): 3134, 1715, 1643, 1444, 1122. ^1H NMR (DMSO-d_6): δ 1.02 (t, J 7.1 Hz, 3 H, OCH_2CH_3), 3.96 (q, J 7.1 Hz, 2 H, $OC\underline{H_2}CH_3$), 7.43–7.56 (m, 8 H, H_{Ar}), 7.62–7.64 (m, 2 H, H_{Ar}), 9.62 (s, 1 H, C^5–H). ^{13}C NMR (DMSO-d_6): δ 13.8, 61.4, 107.0, 111.0, 127.7, 127.8, 128.3, 128.7, 129.5, 129.6, 130.7, 130.9, 160.2, 160.6, 162.8, 163.6, 164.3. MS-EI (*m/z*): 360 $[M]^+$. Found: C, 69.81; H, 4.63; N, 7.65. $C_{21}H_{16}N_2O_4$ requires: C, 69.99; H, 4.48; N, 7.77%.

Preparation of isoxazolines 3c.

Isoxazoline **3c** was synthesized in the same manner as isoxazoles **4a,b,d–g,i–p** (See general procedure). The reaction time is 48 h.

3-Cyclohexyl-*N*,*N*-dimethyl-4-(1-methyl-4-nitro-1*H*-imidazol-5-yl)-4,5-dihydroisoxazol-5-amine (3c). Yellow powder, yield 21%, mp 169–171 °C (column, $CHCl_3$/EtOH 1:1). IR (ν/cm^{-1}): 2929, 2852, 1555, 1504, 1334. ^1H NMR ($CDCl_3$): δ 1.03–1.82 (m, 10 H, $CH_{cyclohex.}$), 2.20–2.78 (m, 1 H, CH), 2.36 (s, 6 H, NMe_2), 3.60 (s, 3 H, CH_3), 5.18 (d, J 4.0 Hz, 1 H, CH), 5.71 (d, J 4.0 Hz, 1 H, CH), 7.37 (s, 1 H, $CH_{imidaz.}$). ^{13}C NMR ($CDCl_3$): δ 25.6, 25.6, 25.8, 29.9, 30.9, 32.5, 37.1, 39.0, 46.2, 70.6, 100.9, 127.8, 137.1, 145.3, 159.0. MS-EI (*m/z*): 321. Found: 56.44; H, 7.52; N, 21.53. $C_{15}H_{23}N_5O_3$ requires: C, 56.06; H, 7.21; N, 21.79%.

Transformation of isoxazolines 3b,c to isoxazoles 4c,h.

A solution isoxazoline **3c** (1 mmol) in a mixture of H_2O:HOAc (1:1) (2 mL) was stirred at room temperature overnight. The formed precipitate was filtered off, washed with H_2O and dried in a desiccator over P_2O_5 and purified by flash column chromatography (CH_2Cl_2).

3-Cyclohexyl-4-(1-methyl-4-nitro-1*H*-imidazol-5-yl)isoxazole (4h). Colorless powder, yield 72%, mp 123−125 °C (column, CH_2Cl_2). IR (ν/cm^{-1}): 3133, 2930, 2855, 1496, 1331. ^1H NMR ($CDCl_3$): δ 1.16–1.75 (m, 10 H, CH_2), 2.48–2.54 (m, 1 H, CH), 3.55 (s, 3 H, CH_3), 7.59 (s, 1 H, C^5–H), 8.47 (s, 1 H, $CH_{imidaz.}$). ^{13}C NMR ($CDCl_3$): δ 25.7, 26.0, 33.0, 35.9, 105.6, 121.0, 136.8, 146.4, 158.3, 166.4. MS-EI (*m/z*): 276 $[M]^+$. Found: C, 56.88; H, 6.07; N, 19.97. $C_{13}H_{16}N_4O_3$ requires: C, 56.51; H, 5.84; N, 20.28%.
