# Supplementary material for: Combined experimental and theoretical studies of regio- and stereoselectivity in reactions of β-isoxazolyl- and β-imidazolyl enamines with nitrile oxides
Source: Beilstein J Org Chem. 2016 Nov 15;12:2390–401. doi: 10.3762/bjoc.12.233 (PMC5238569; doi:10.3762/bjoc.12.233)
Supplement: File 2 — NMR spectra of compounds 3a–c, 4a–p and X-ray study of isoxazoles 4a,o,p. [file Beilstein_J_Org_Chem-12-2390-s002.pdf]

Supporting Information

for

Combined experimental and theoretical studies of regio- and stereoselectivity in reactions of β-isoxazolyl- and β-imidazolyl enamines with nitrile oxides

Ilya V. Efimov[1], Marsel Z. Shafikov[1,2], Nikolai A. Beliaev[1], Natalia N. Volkova[1], Tetyana V. Beryozkina[1], Wim Dehaen[3], Zhijin Fan*[4], Viktoria V. Grishko[5], Gert Lubec[6], Pavel A. Slepukhin[1,7] and Vasiliy A. Bakulev*[1]

Address: [1]TOS Department, Ural Federal University named after the first President of Russia B.N. Yeltsin, 19 Mira str., 620002 Yekaterinburg, Russia, [2]Chemistry Department, University of York, Heslington, York, YO10 5DD (UK), [3]Molecular Design and Synthesis, Department of Chemistry, KU Leuven, Celestijnenlaan 200F, 3001 Leuven, Belgium, [4]State Key Laboratory of Elemento-Organic Chemistry Nankai University, Collaborative Innovation Center of Chemical Science and Engineering 300071 Tianjin, China, [5]Laboratory of Biologically Active Compounds, Institute of Technical Chemistry Ural Branch of Russian Academy of Sciences, 3 Academician Korolev str., Perm, Russia, [6]Medical University of Vienna, Department of Pediatrics, 14 Lazarettgasse, 1090 Vienna, Austria and [7]I. Ya. Postovsky Institute of Organic Synthesis, Ural Branch of Russian Academy of Sciences, 20 S. Kovalevskaya str., 620990 Yekaterinburg, Russia

Email: Vasiliy A. Bakulev - v.a.bakulev@urfu.ru; Zhijin Fan - fanj@nankai.edu.cn
*Corresponding author

NMR spectra of compounds 3a–c, 4a–p and X-ray study of isoxazoles 4a,o,p

Contents

X-ray diffraction study S2
NMR spectra S4

X-ray diffraction study

X-ray analyses were accomplished on an Xcalibur 3 diffractometer using the standard procedure (graphite-monochromated MoK-irradiation, ω-scanning with step 1o, T = 150.00(10) K) (**4o,p**) or 295(2) K (**4a**).

4a. $C_{13}H_{10}N_4O_3$, M = 270.25, orthorhombic, a = 6.5164(4) Å, b = 10.4597(4) Å, c = 17.7719(8) Å, V = 1211.33(10) Å^3, space group $P2_12_12_1$ (no. 19), Z = 4, μ(Mo Kα) = 0.109 mm^{-1}, D_{calc} = 1.4818 g/cm^3, 4294 reflections measured (4.52° ≤ 2Θ ≤ 61.22°), 3018 unique (R_{int} = 0.0169, R_{sigma} = 0.0324) which were used in all calculations. The final R_1 was R_1 = 0.0440, wR_2 = 0.1032 (I>=2σ(I)) and R_1 = 0.0565, wR_2 = 0.1112 (all data). Goodness-of-fit on F^2 1.055, largest diff. peak/hole 0.17/-0.25 ēÅ^{-3}.

4o. $C_{20}H_{15}N_3O_4$, M =361.35, monoclinic, a = 8.9292(3) Å, b = 12.2608(4) Å, c = 15.9631(6) Å, β = 101.183(3)°, V = 1714.45(10) Å^3, T = 150.00(10), space group $P2_1/c$ (no. 14), Z = 4, μ(MoKα) = 0.100 mm^{-1}, 8807 reflections measured, 4683 unique (R_{int} = 0.0263) which were used in all calculations. The final wR_2 was 0.1333 (all data) and R_1 was 0.0453 (I>2σ(I)).

4p. $C_{21}H_{16}N_2O_4$, M =360.36, monoclinic, a = 8.9596(4) Å, b = 12.5516(3) Å, c = 15.7754(4) Å, β = 100.144(3)°, V = 1746.33(10) Å^3, T = 150.00(10), space group $P2_1/c$ (no. 14), Z = 4, μ(MoKα) = 0.096 mm^{-1}, 8512 reflections measured, 4626 unique (R_{int} = 0.0208) which were used in all calculations. The final wR_2 was 0.1379 (all data) and R_1 was 0.0438 (I>2σ(I)).

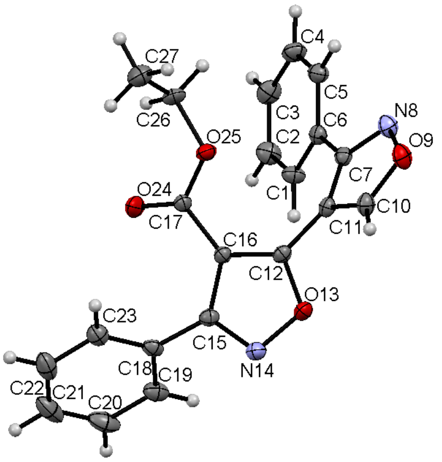

NMR Spectra of isoxazoles 4a–p description. The ^{1}H and ^{13}C NMR spectra for products **4a–p** demonstrate that all of the compounds belong to the same structural type. The ^{1}H NMR spectra of isoxazoles **4a–p** contain signals of C^5–H of isoxazole ring in the range of 9.42–9.85 ppm apart from 3-cyclohexyl isoxazoles **4e,h,m** and 2,6-dihalophenylisoxazoles **4d,g,l** which are out of this range and exhibit those signals at 9.19 and 10.04–10.32 ppm, respectively. ^{13}C NMR spectra of **4a–p** show the signals of the carbon atoms at the 3-, 4- and 5-positions of the new isoxazole ring at 159.4−161.4 ppm, 105.8–110.5 and 161.2–165.5 ppm, respectively. 2D NMR spectra show direct coupling of C^5 with H, that could be diagnostic for 3,4-disubstituted

isoxazoles **4a-p**. For the alternative 3,5-disubstituted isoxazoles **5** we would have expected to observe the direct coupling of H with the C^4–H carbon. One can conclude that, 3,4- and 3,5-disubstituted isoxazoles could be easily distinguished by NMR spectroscopy due to the structural features.

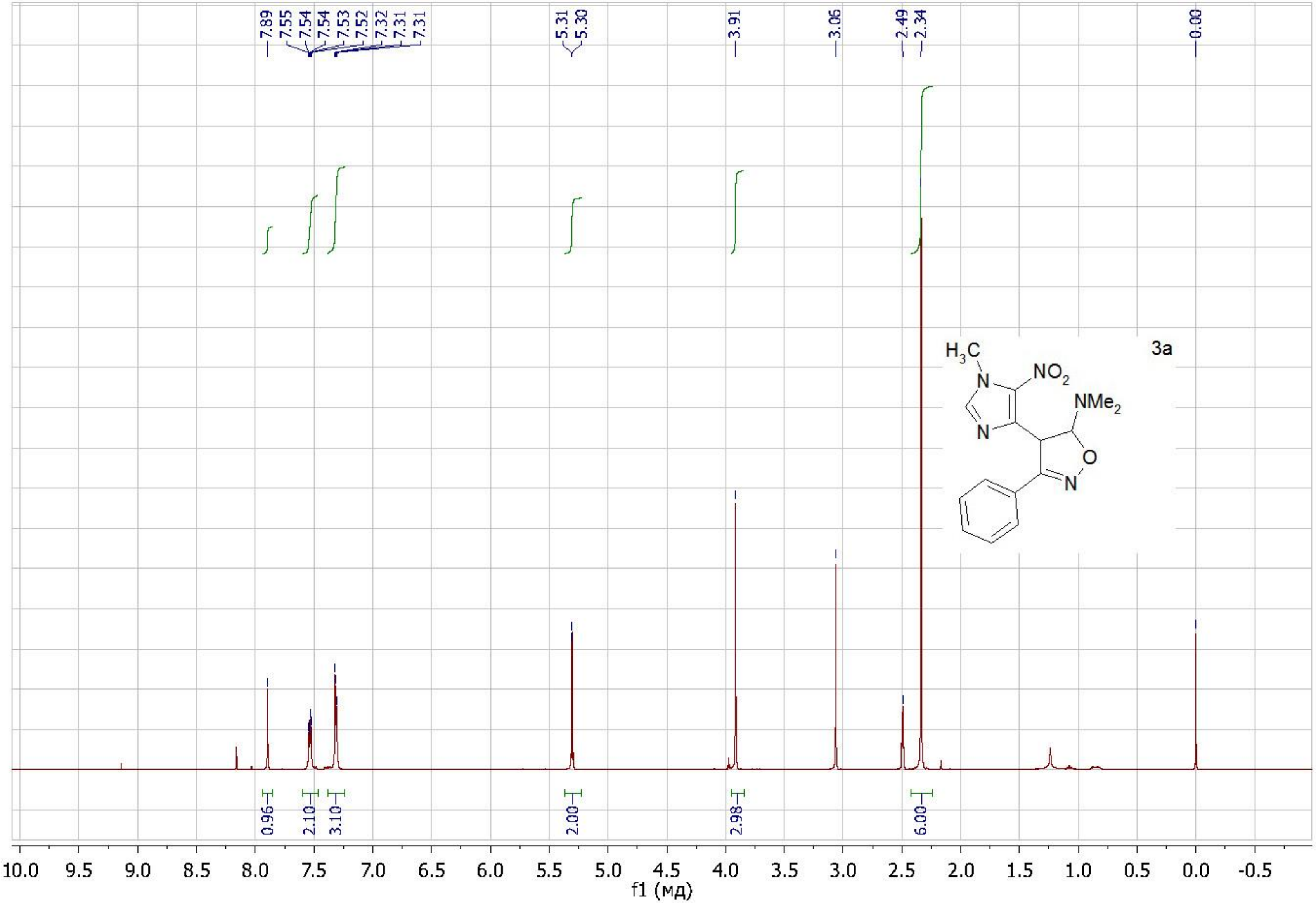
7.89
7.55
7.54
7.54
7.53
7.52
7.32
7.31
7.31
5.31
5.30
3.91
3.06
2.49
2.34
0.00
3a
H3C
N
NO2
NMe2
N
O
N
0.96
2.10
3.10
2.00
2.98
6.00
10.0
9.5
9.0
8.5
8.0
7.5
7.0
6.5
6.0
5.5
5.0
4.5
4.0
3.5
3.0
2.5
2.0
1.5
1.0
0.5
0.0
-0.5
f1 (мд)

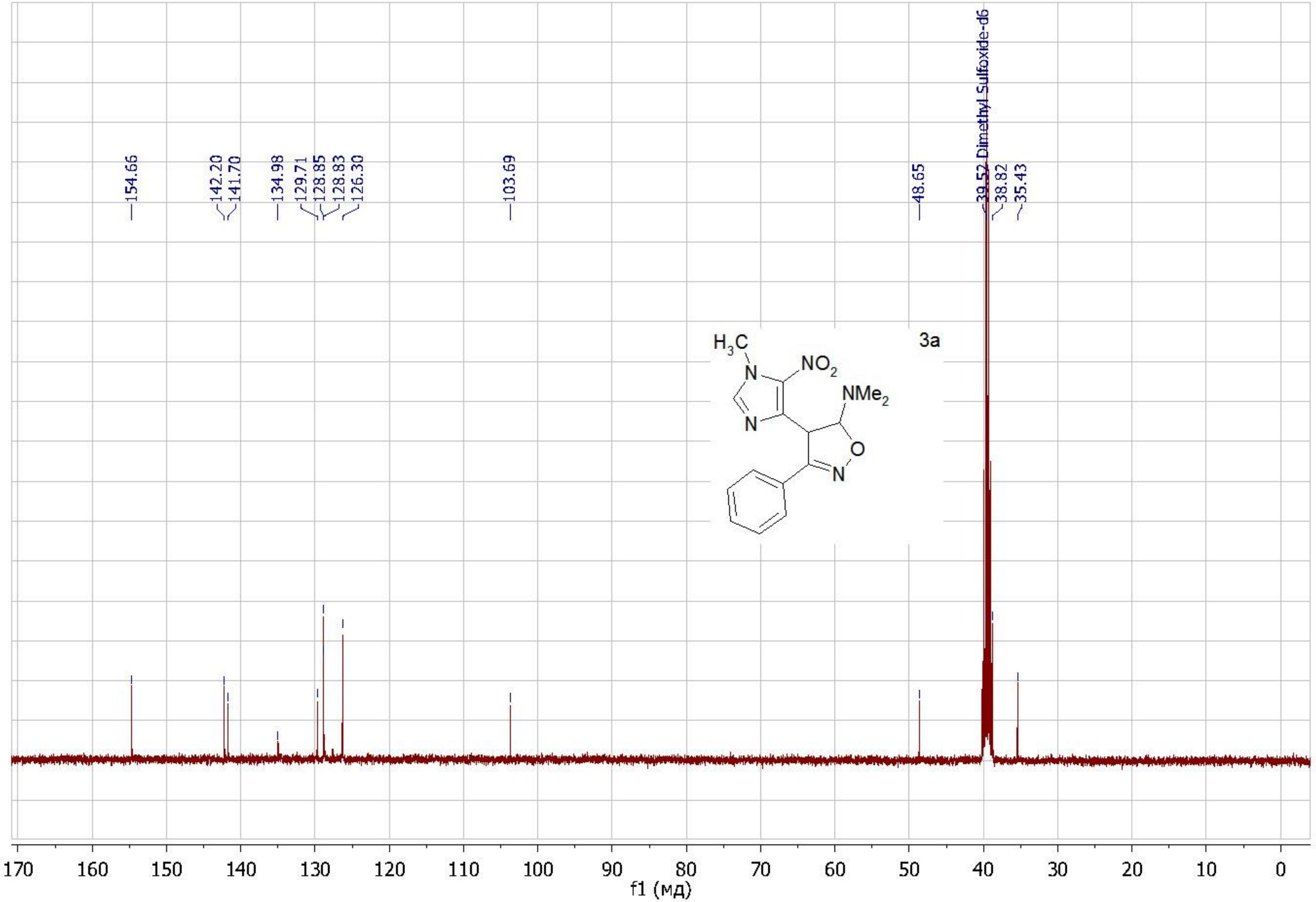

154.66
142.20
141.70
134.98
129.71
128.85
128.83
126.30
103.69
48.65
39.52 Dimethyl Sulfoxide-d6
38.82
35.43
H3C
NO2
NMe2
N
N
O
N
3a
170
160
150
140
130
120
110
100
90
80
70
60
50
40
30
20
10
0
f1 (мд)

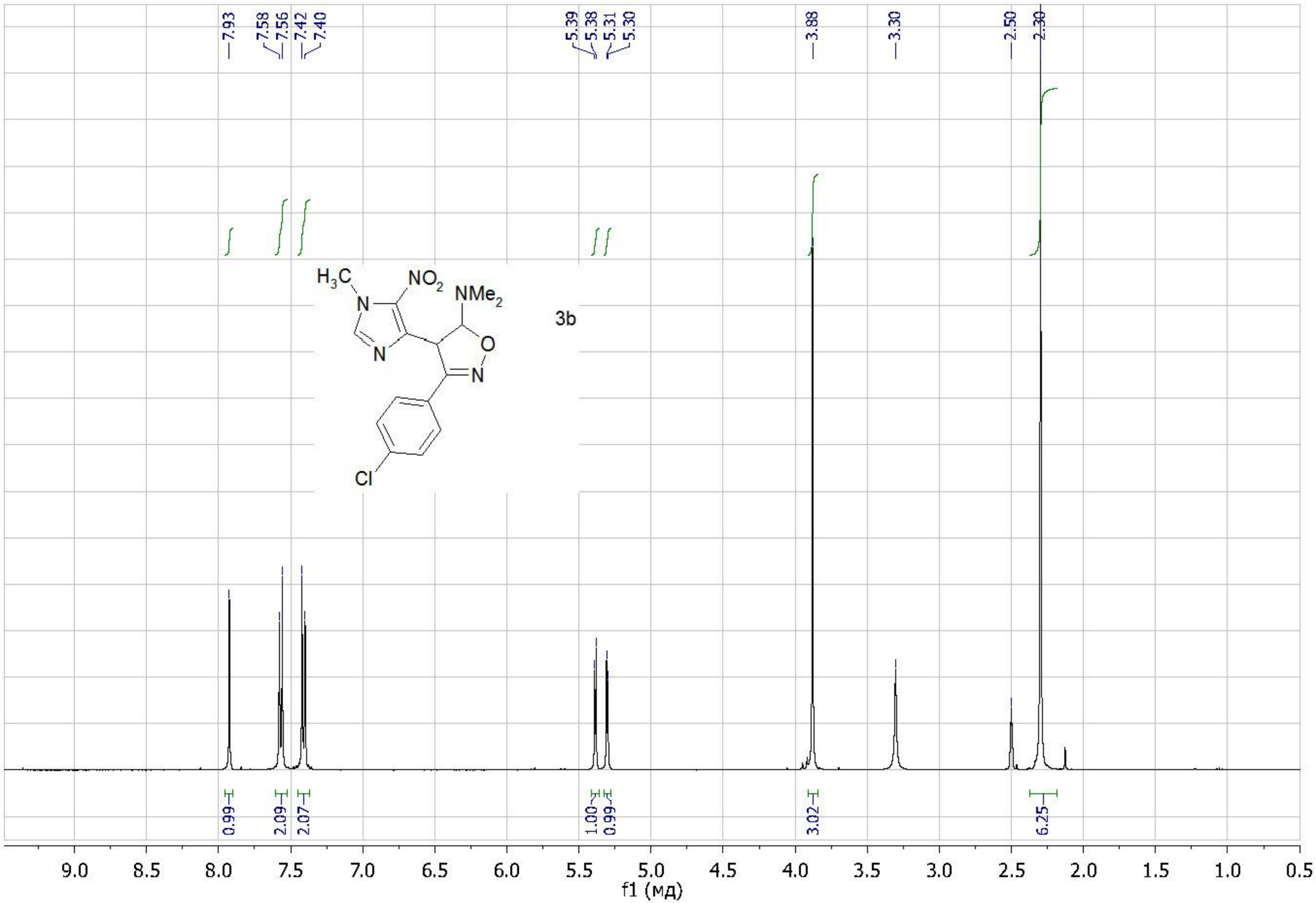

7.93
7.58
7.56
7.42
7.40
5.39
5.38
5.31
5.30
3.88
3.30
2.50
2.30
H_3C
NO_2
NMe_2
N
N
O
N
Cl
3b
0.99
2.09
2.07
1.00
0.99
3.02
6.25
9.0
8.5
8.0
7.5
7.0
6.5
6.0
5.5
5.0
4.5
4.0
3.5
3.0
2.5
2.0
1.5
1.0
0.5
f1 (мд)

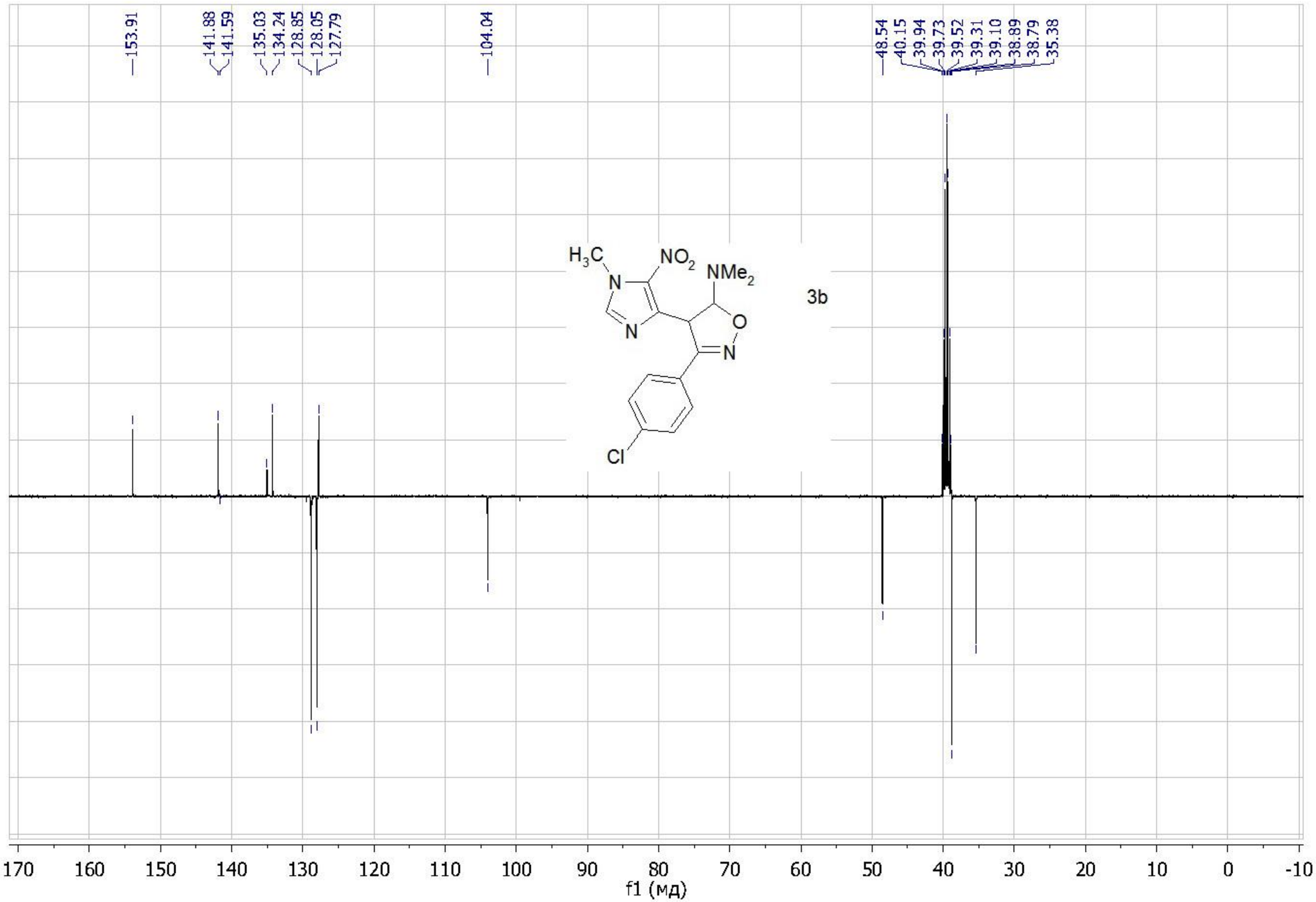
153.91
141.88
141.59
135.03
134.24
128.85
128.05
127.79
104.04
48.54
40.15
39.94
39.73
39.52
39.31
39.10
38.89
38.79
35.38
H_3C
NO_2
NMe_2
N
N
O
N
Cl
3b
170 160 150 140 130 120 110 100 90 80 70 60 50 40 30 20 10 0 -10
f1 (мд)

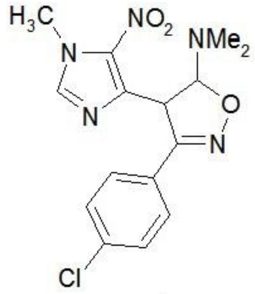
H3C
NO2
NMe2
N
N
O
N
Cl

3b

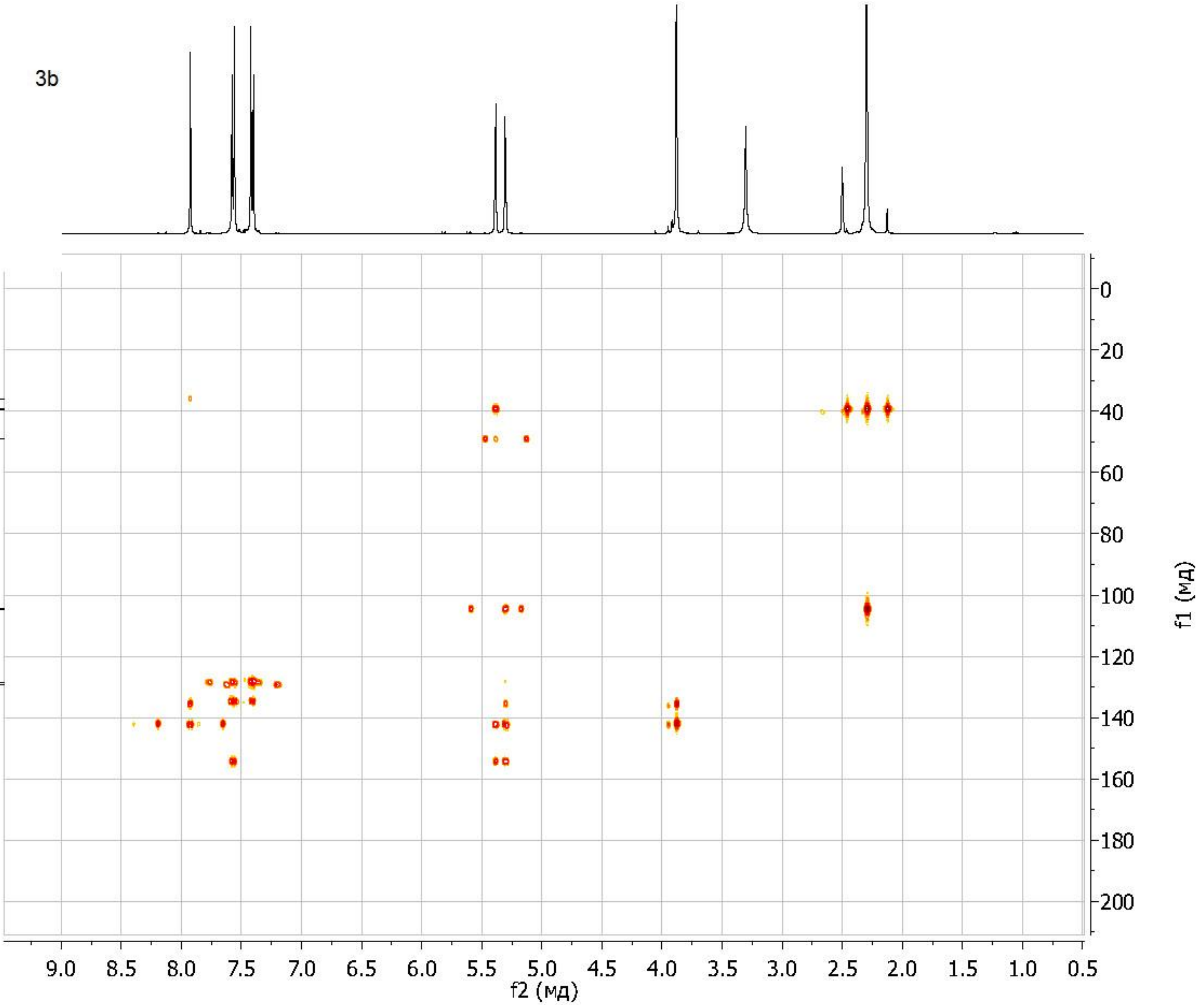
9.0 8.5 8.0 7.5 7.0 6.5 6.0 5.5 5.0 4.5 4.0 3.5 3.0 2.5 2.0 1.5 1.0 0.5
f2 (мд)
0 20 40 60 80 100 120 140 160 180 200
f1 (мд)

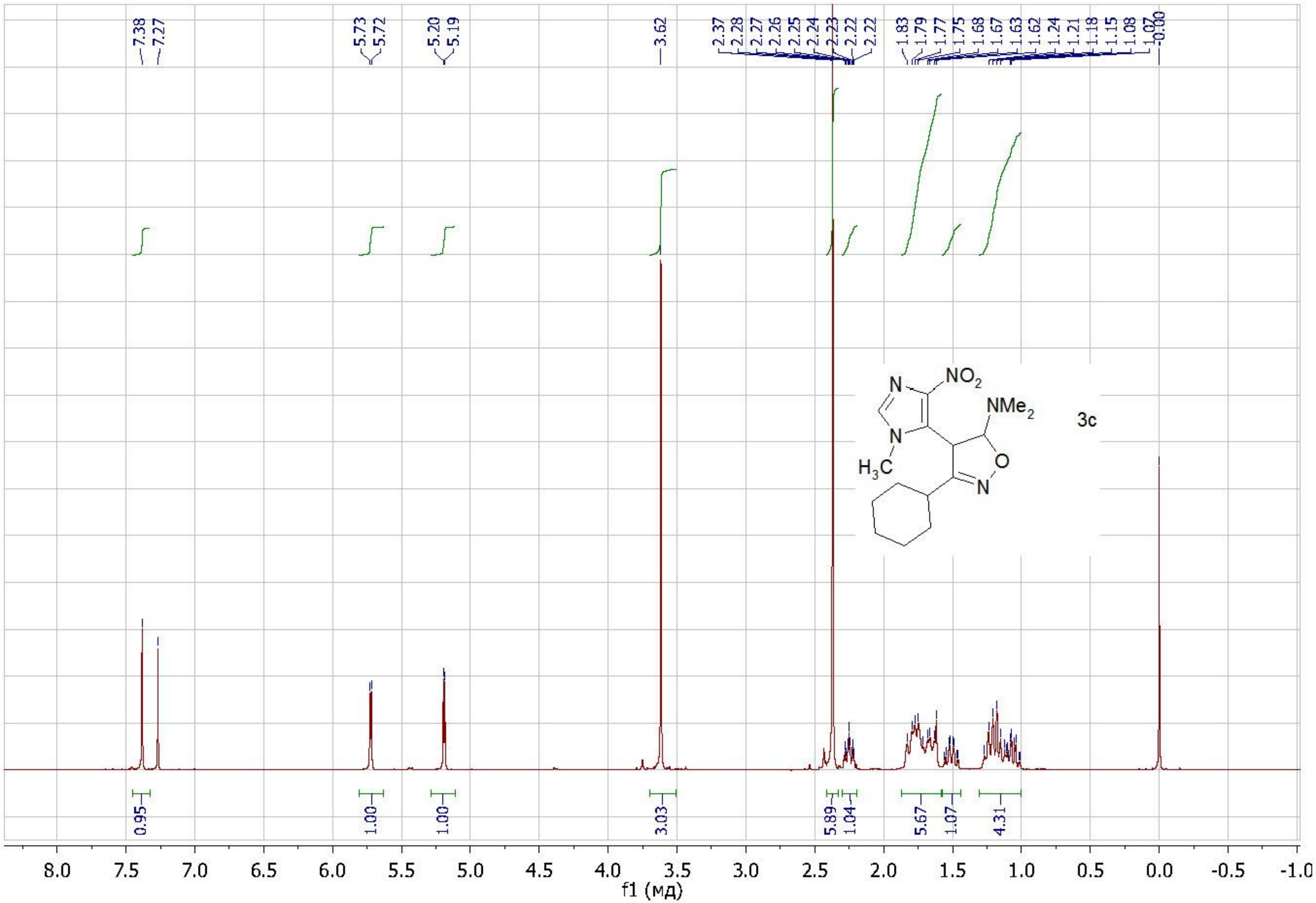
7.38
7.27
5.73
5.72
5.20
5.19
3.62
2.37
2.28
2.27
2.26
2.25
2.24
2.23
2.22
2.22
1.83
1.79
1.77
1.75
1.68
1.67
1.63
1.62
1.24
1.21
1.18
1.15
1.08
1.07
0.00
NO2
N
NMe2
N
H3C
O
N
3c
0.95
1.00
1.00
3.03
5.89
1.04
5.67
1.07
4.31
8.0
7.5
7.0
6.5
6.0
5.5
5.0
4.5
4.0
3.5
3.0
2.5
2.0
1.5
1.0
0.5
0.0
-0.5
-1.0
f1 (мд)

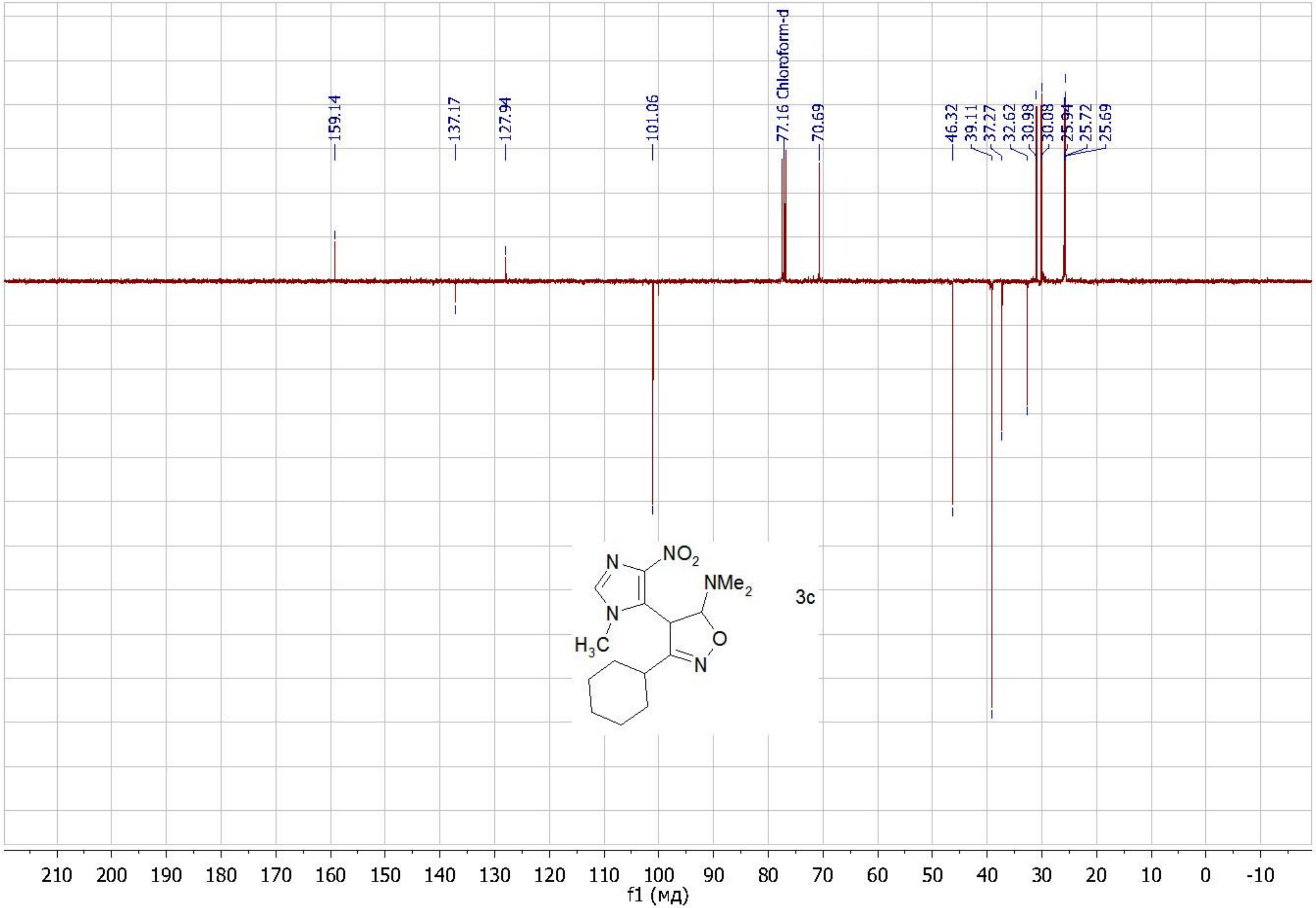

159.14
137.17
127.94
101.06
77.16 Chloroform-d
70.69
46.32
39.11
37.27
32.62
30.98
30.08
25.94
25.72
25.69
NO2
N
NMe2
N
H3C
O
N
210 200 190 180 170 160 150 140 130 120 110 100 90 80 70 60 50 40 30 20 10 0 -10
f1 (мд)

3c

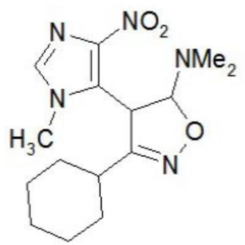

3c

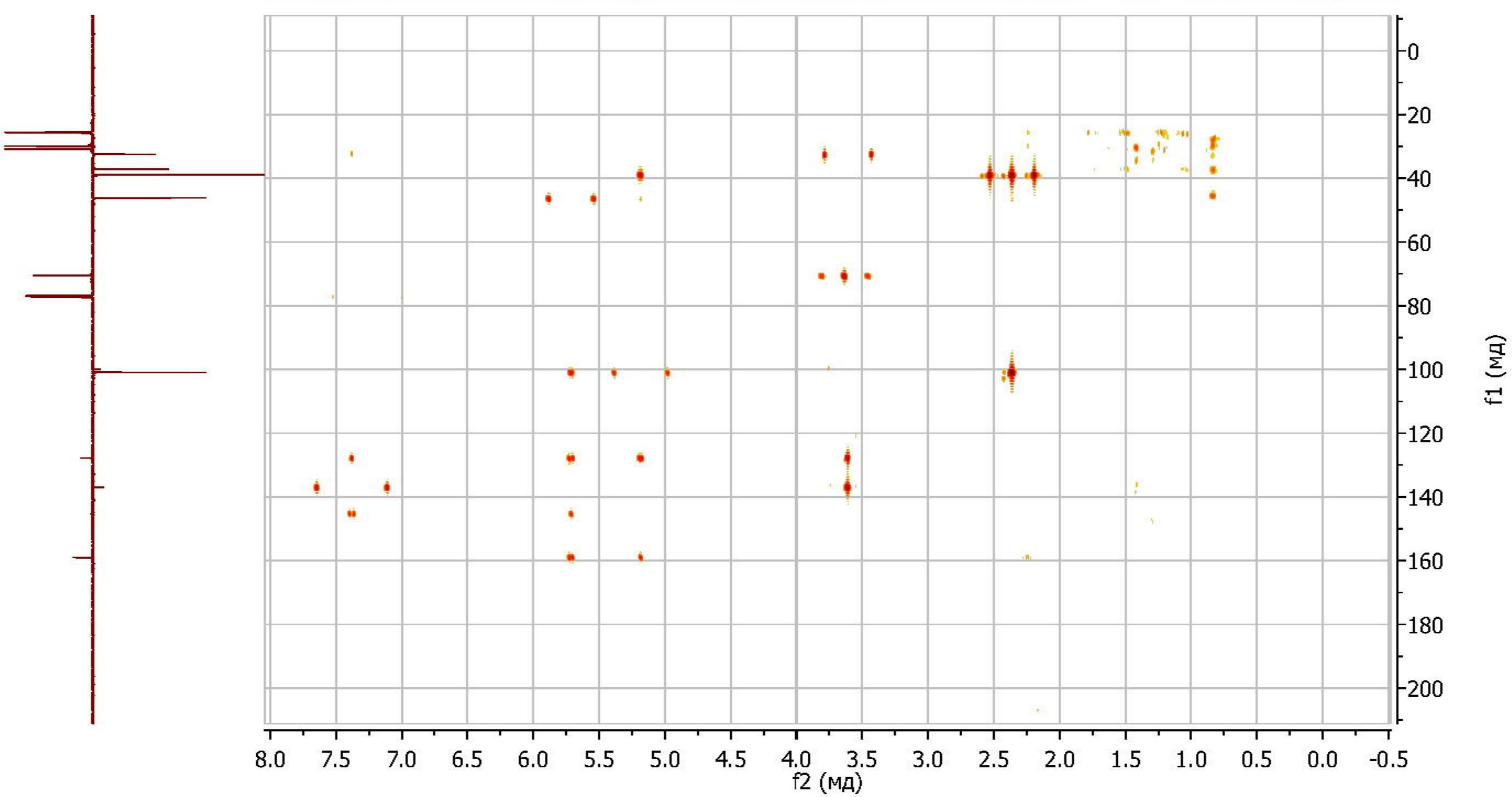

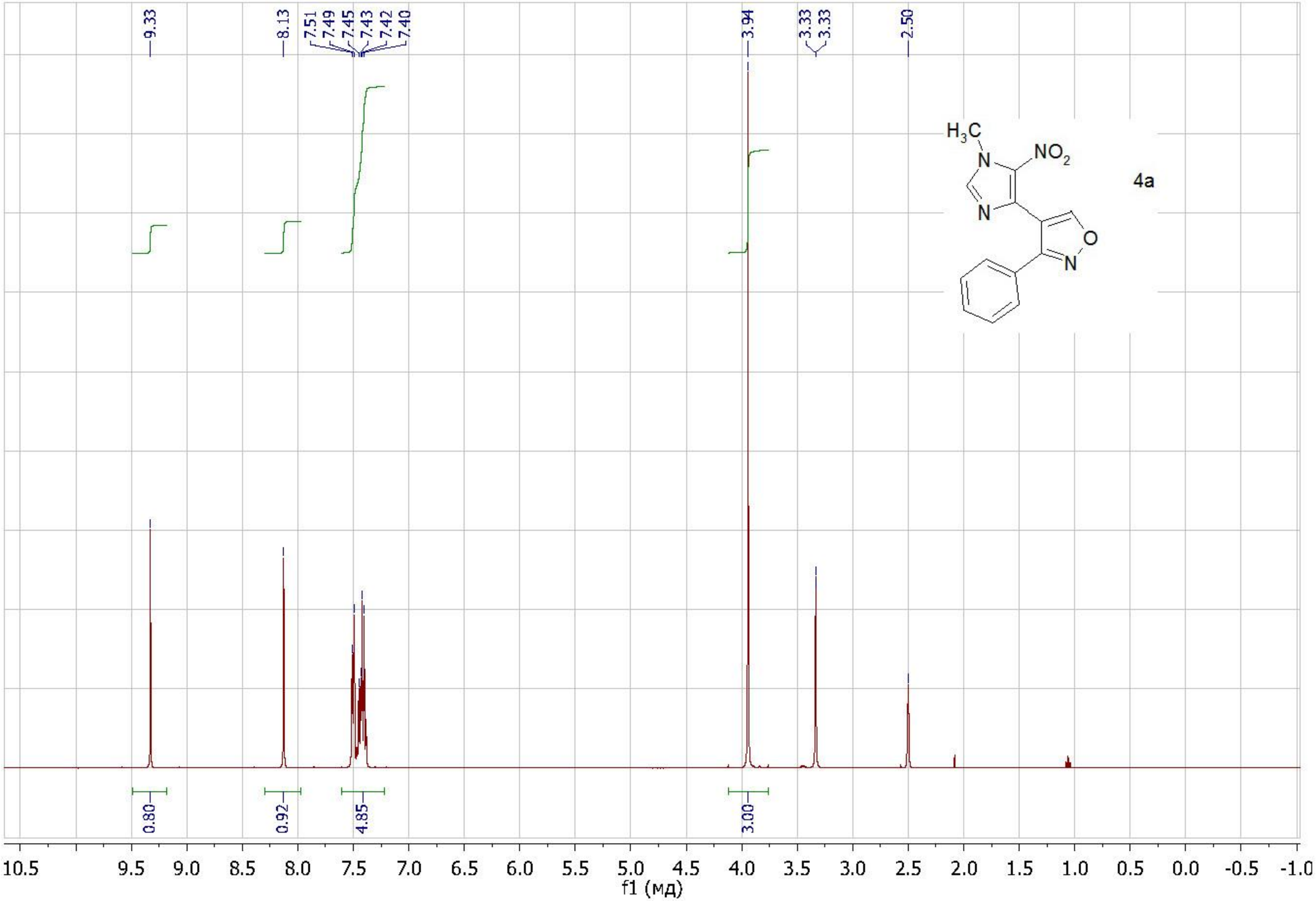
9.33
8.13
7.51
7.49
7.45
7.43
7.42
7.40
3.94
3.33
3.33
2.50
H3C
NO2
N
N
O
N
4a
0.80
0.92
4.85
3.00
10.5
9.5
9.0
8.5
8.0
7.5
7.0
6.5
6.0
5.5
5.0
4.5
4.0
3.5
3.0
2.5
2.0
1.5
1.0
0.5
0.0
-0.5
-1.0
f1 (мд)

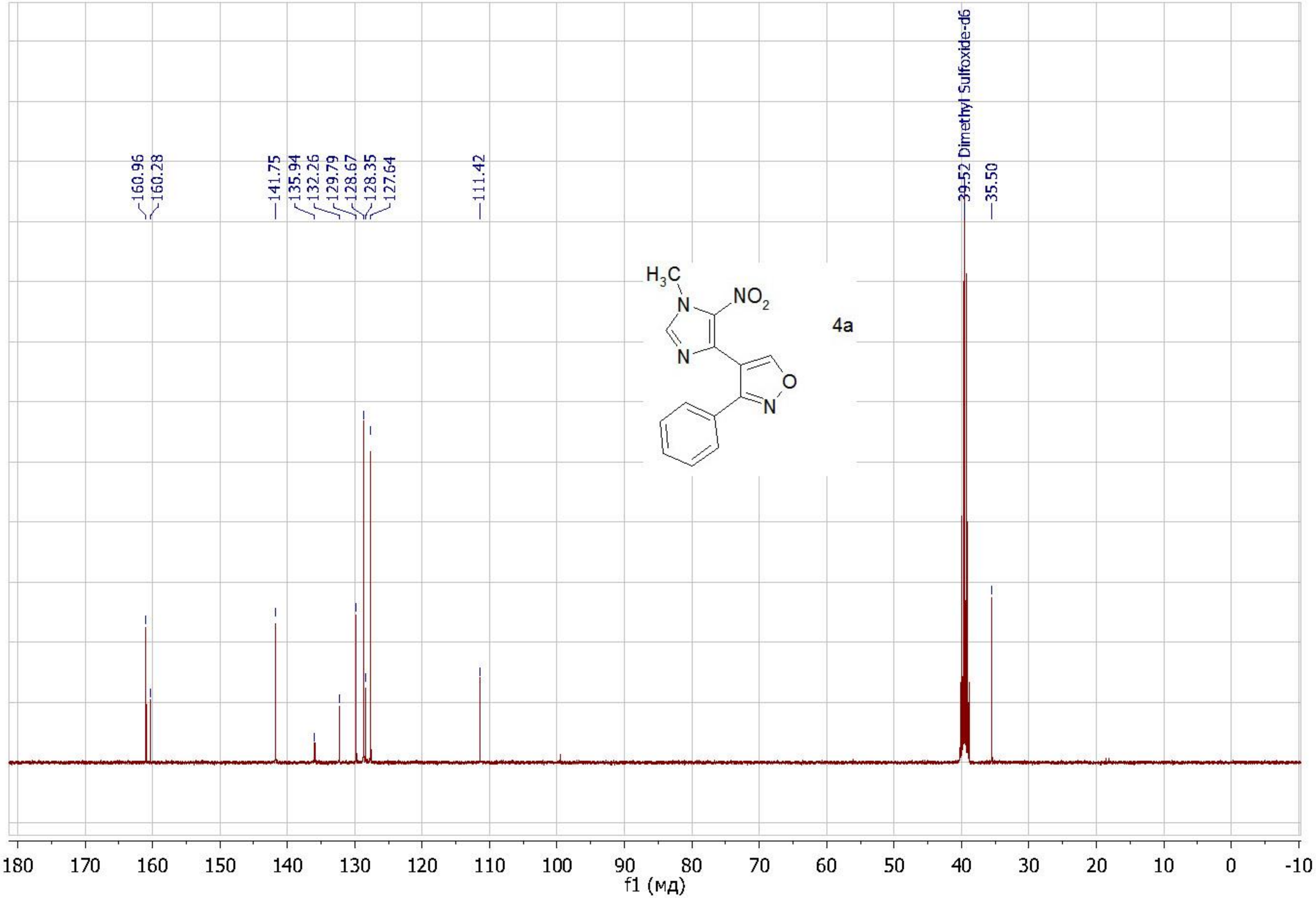
160.96
160.28
141.75
135.94
132.26
129.79
128.67
128.35
127.64
111.42
39.52 Dimethyl Sulfoxide-d6
35.50
H3C
NO2
N
N
O
N
4a
180 170 160 150 140 130 120 110 100 90 80 70 60 50 40 30 20 10 0 -10
f1 (мд)

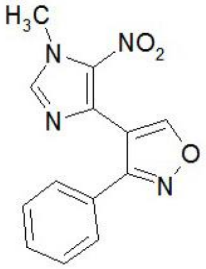
H3C
NO2
N
N
O
N

4a

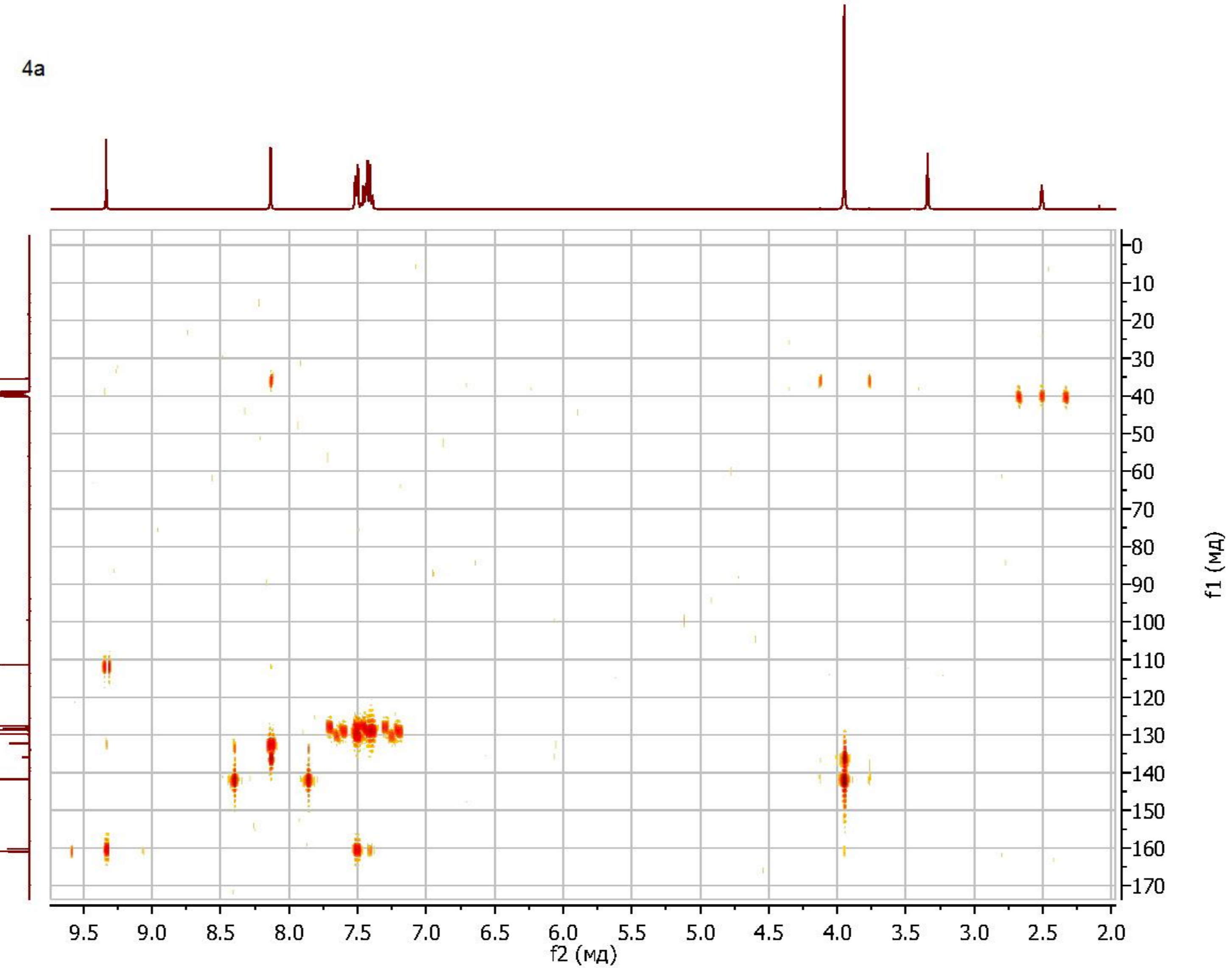
f2 (мд)
f1 (мд)
9.5 9.0 8.5 8.0 7.5 7.0 6.5 6.0 5.5 5.0 4.5 4.0 3.5 3.0 2.5 2.0
0 10 20 30 40 50 60 70 80 90 100 110 120 130 140 150 160 170

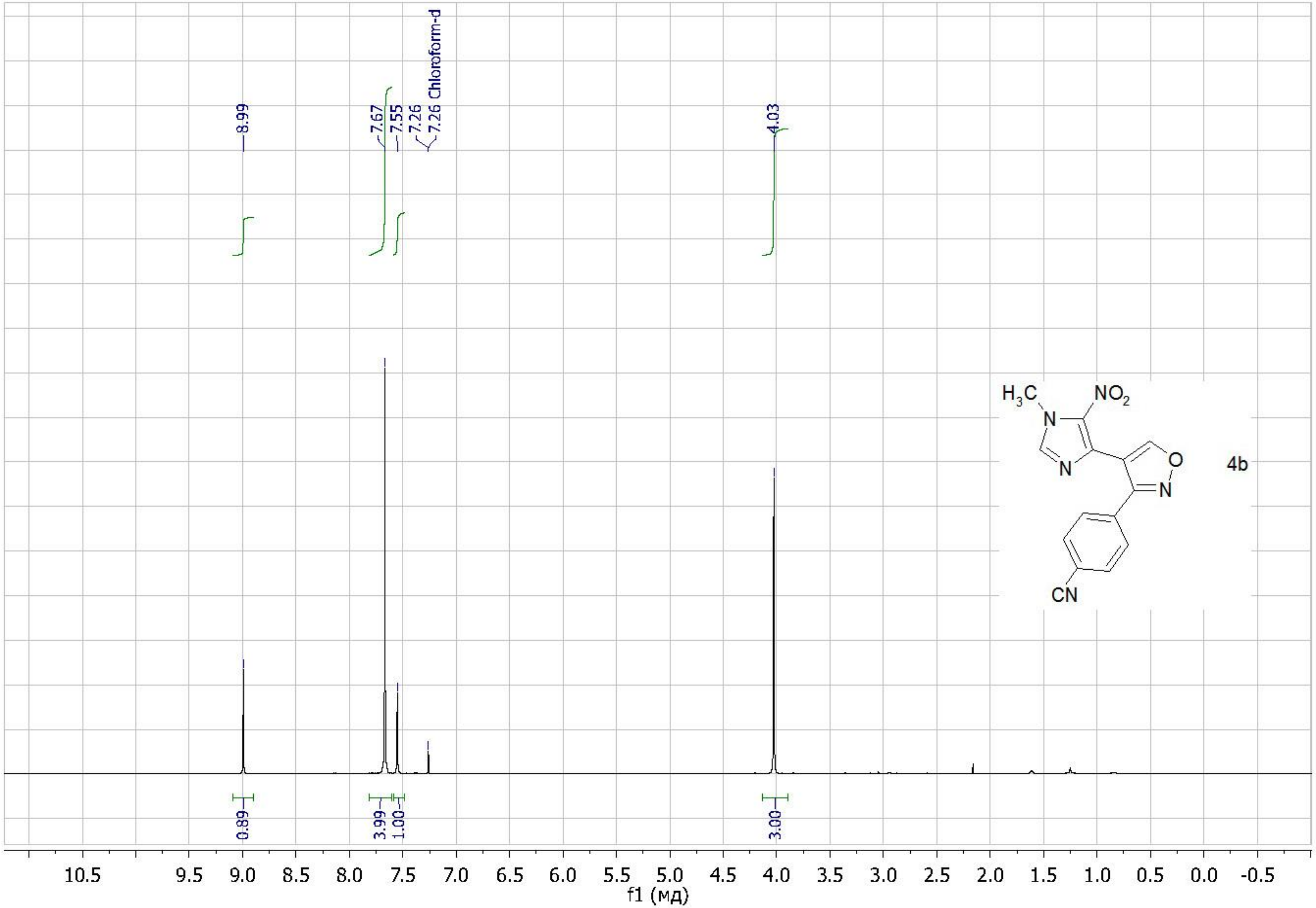

8.99
7.67
7.55
7.26
7.26 Chloroform-d
4.03
0.89
3.99
1.00
3.00
10.5
9.5
9.0
8.5
8.0
7.5
7.0
6.5
6.0
5.5
5.0
4.5
4.0
3.5
3.0
2.5
2.0
1.5
1.0
0.5
0.0
-0.5
f1 (мд)
H_3C
NO_2
N
N
O
N
CN
4b

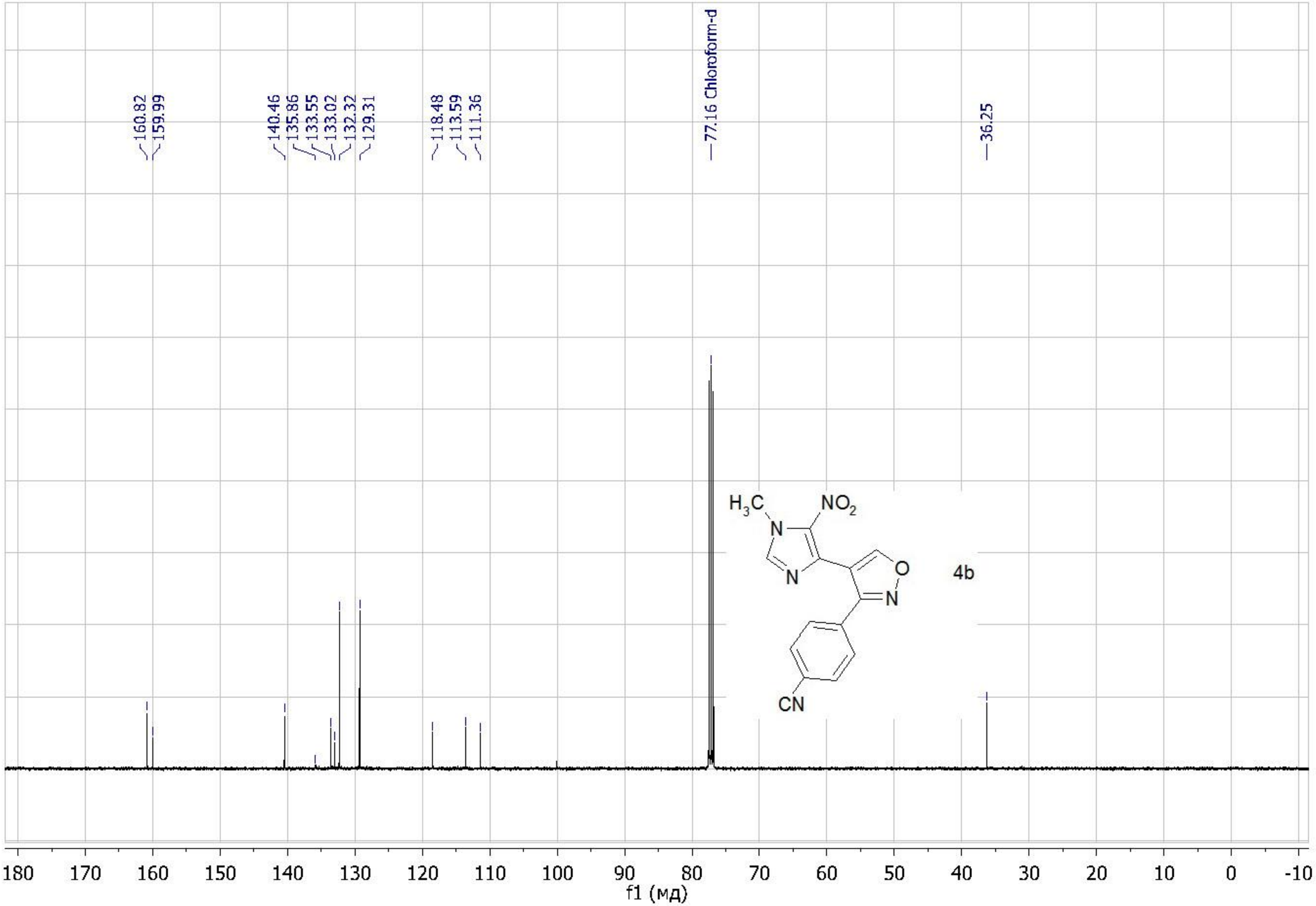

160.82
159.99
140.46
135.86
133.55
133.02
132.32
129.31
118.48
113.59
111.36
77.16 Chloroform-d
36.25
H3C
NO2
N
N
O
N
CN
4b
180 170 160 150 140 130 120 110 100 90 80 70 60 50 40 30 20 10 0 -10
f1 (мд)

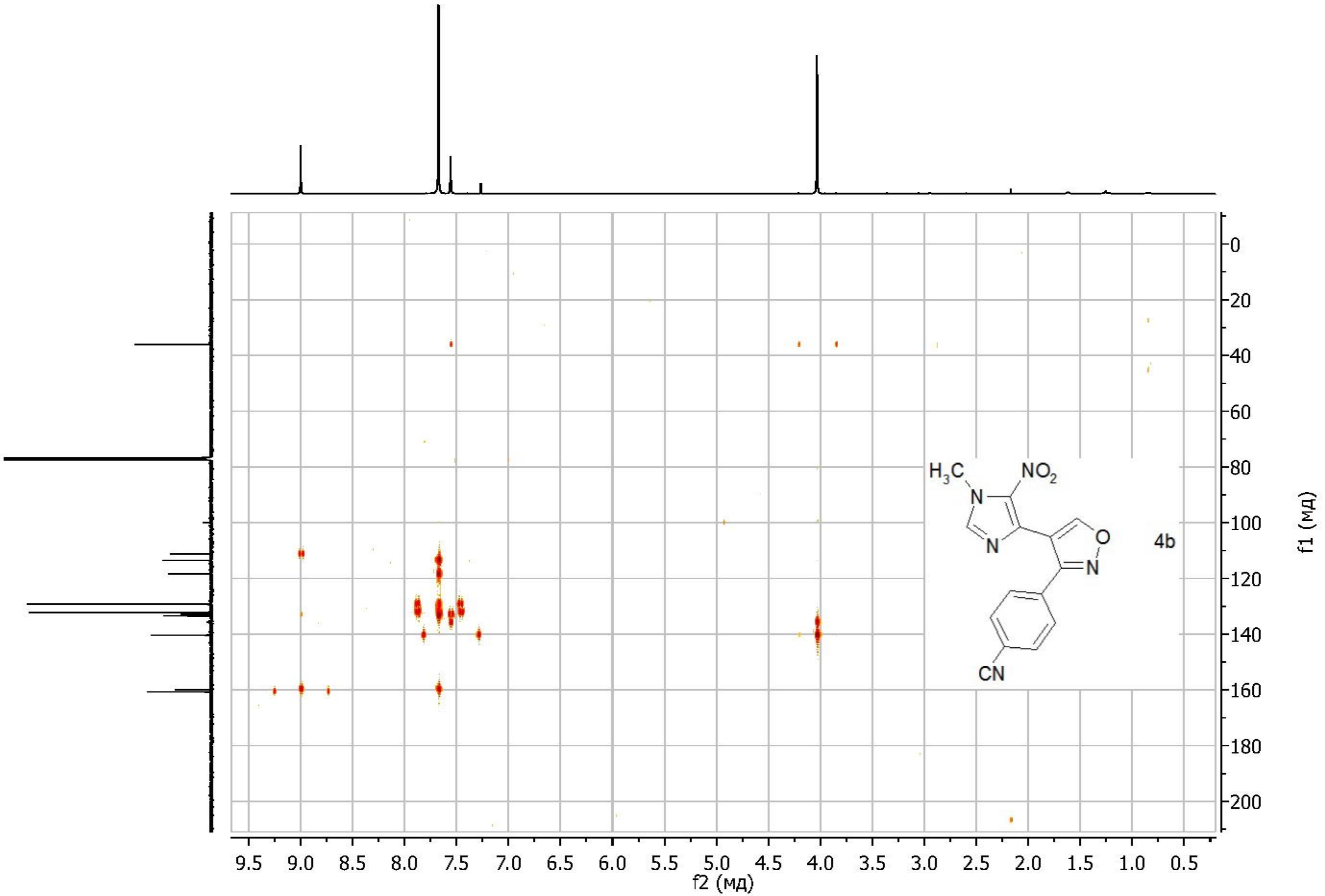

4b
f2 (мд)
f1 (мд)

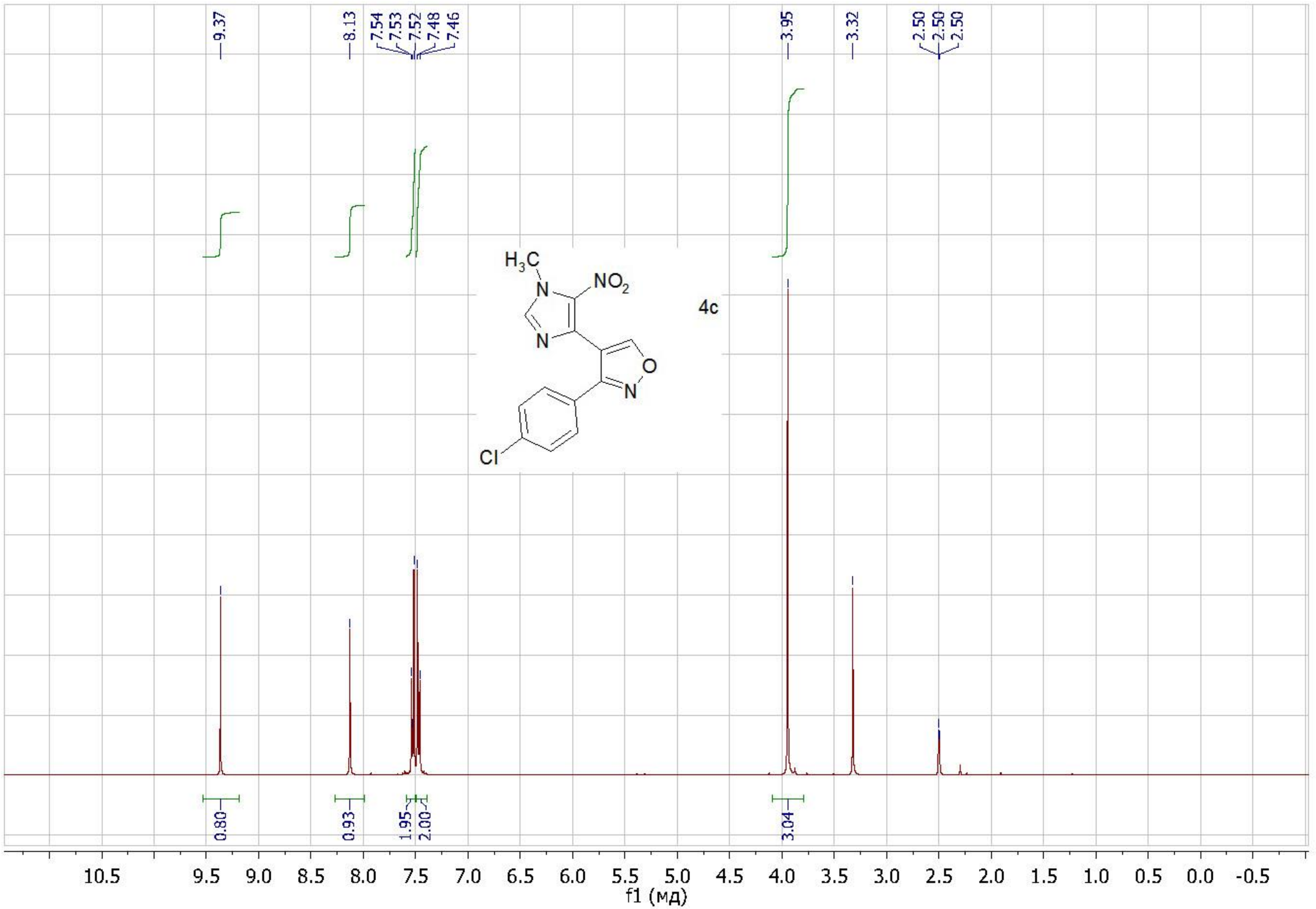

9.37
8.13
7.54
7.53
7.52
7.48
7.46
3.95
3.32
2.50
2.50
2.50
H3C
N
NO2
N
O
N
Cl
4c
0.80
0.93
1.95
2.00
3.04
10.5
9.5
9.0
8.5
8.0
7.5
7.0
6.5
6.0
5.5
5.0
4.5
4.0
3.5
3.0
2.5
2.0
1.5
1.0
0.5
0.0
-0.5
f1 (мд)

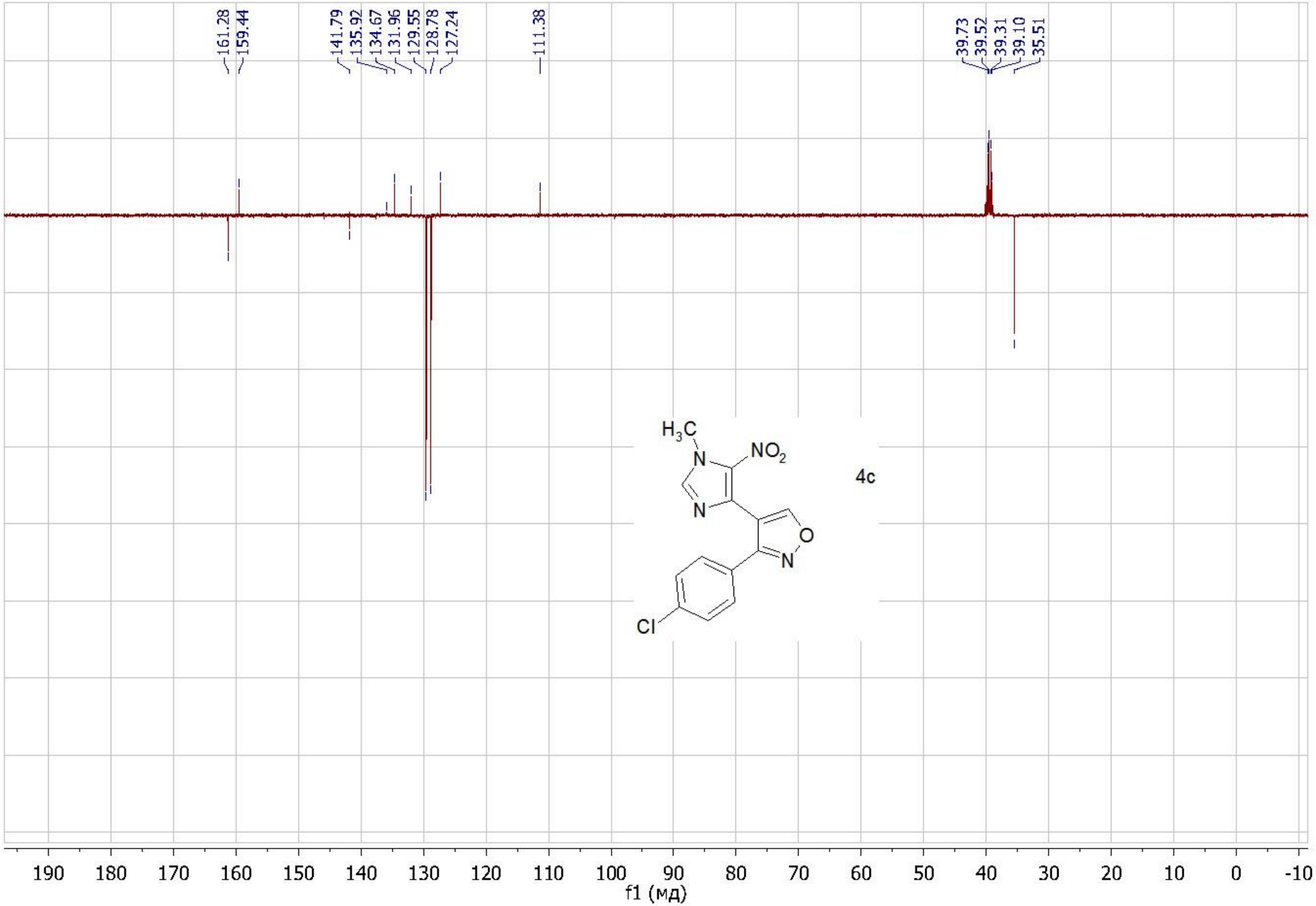
161.28
159.44
141.79
135.92
134.67
131.96
129.55
128.78
127.24
111.38
39.73
39.52
39.31
39.10
35.51
H3C
NO2
N
N
O
N
Cl
4c
190 180 170 160 150 140 130 120 110 100 90 80 70 60 50 40 30 20 10 0 -10
f1 (мд)

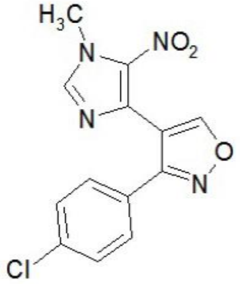

4c

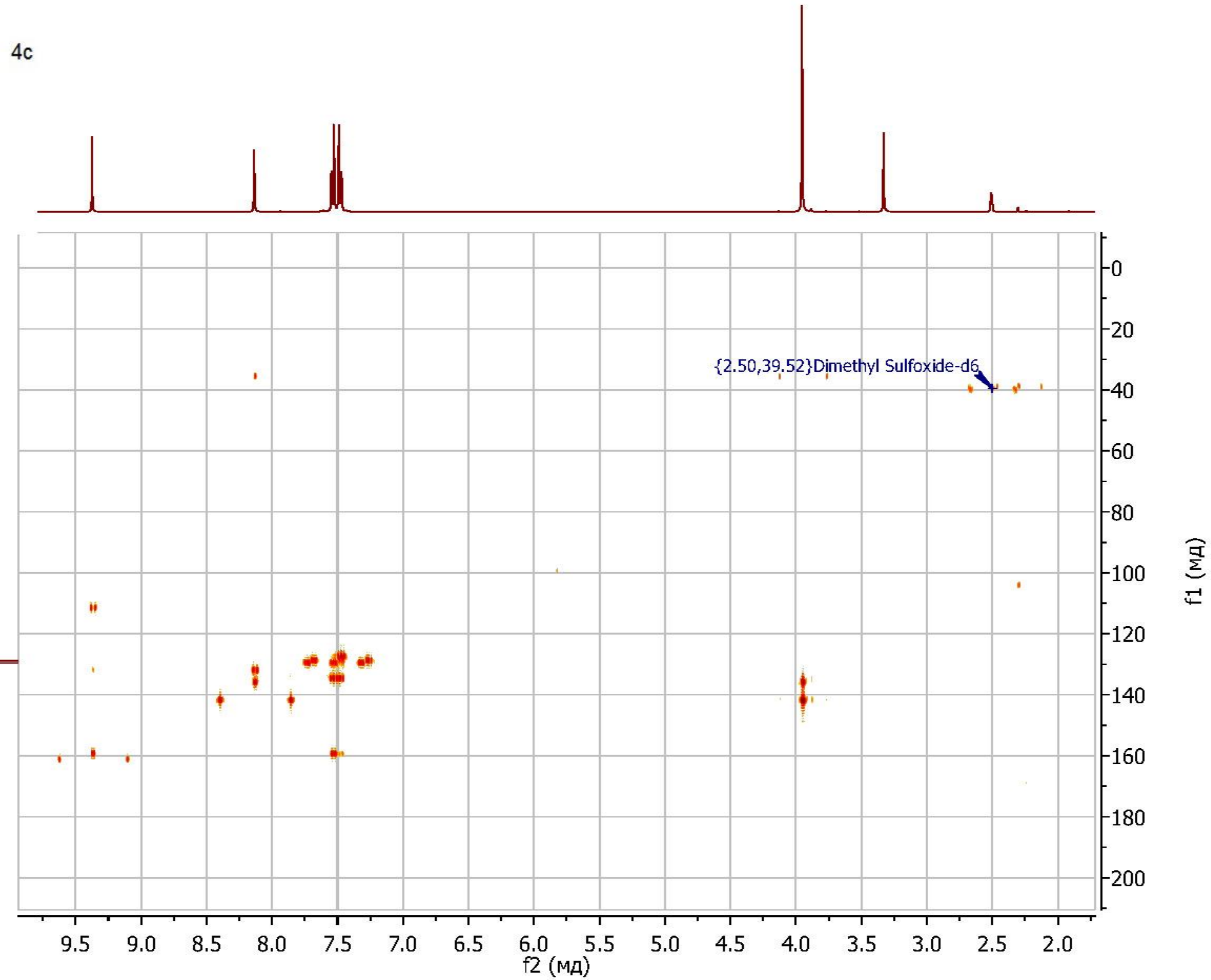

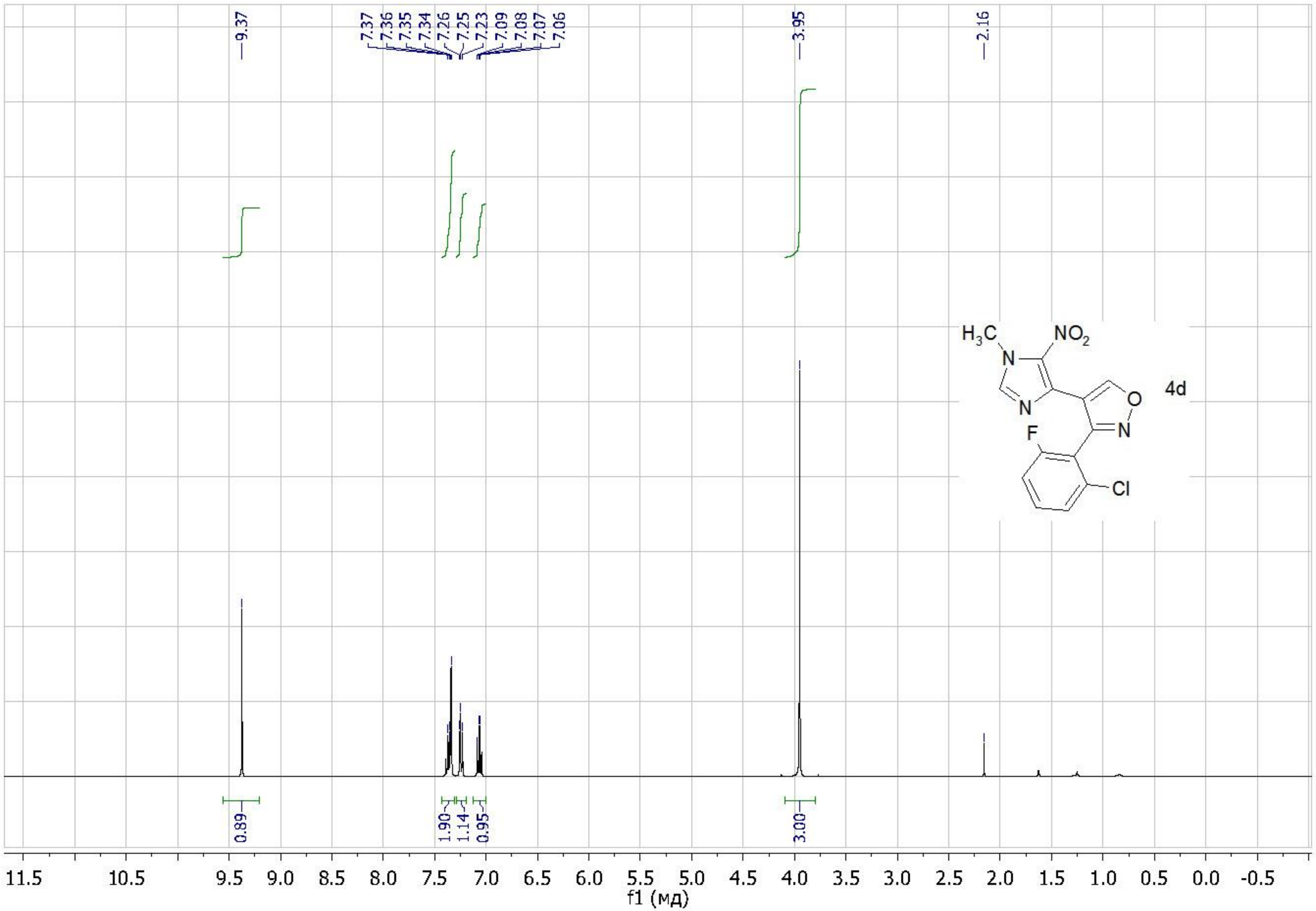

9.37
7.37
7.36
7.35
7.34
7.26
7.25
7.23
7.09
7.08
7.07
7.06
3.95
2.16
H3C
NO2
N
N
O
N
F
Cl
4d
0.89
1.90
1.14
0.95
3.00
11.5
10.5
9.5
9.0
8.5
8.0
7.5
7.0
6.5
6.0
5.5
5.0
4.5
4.0
3.5
3.0
2.5
2.0
1.5
1.0
0.5
0.0
-0.5
f1 (мд)

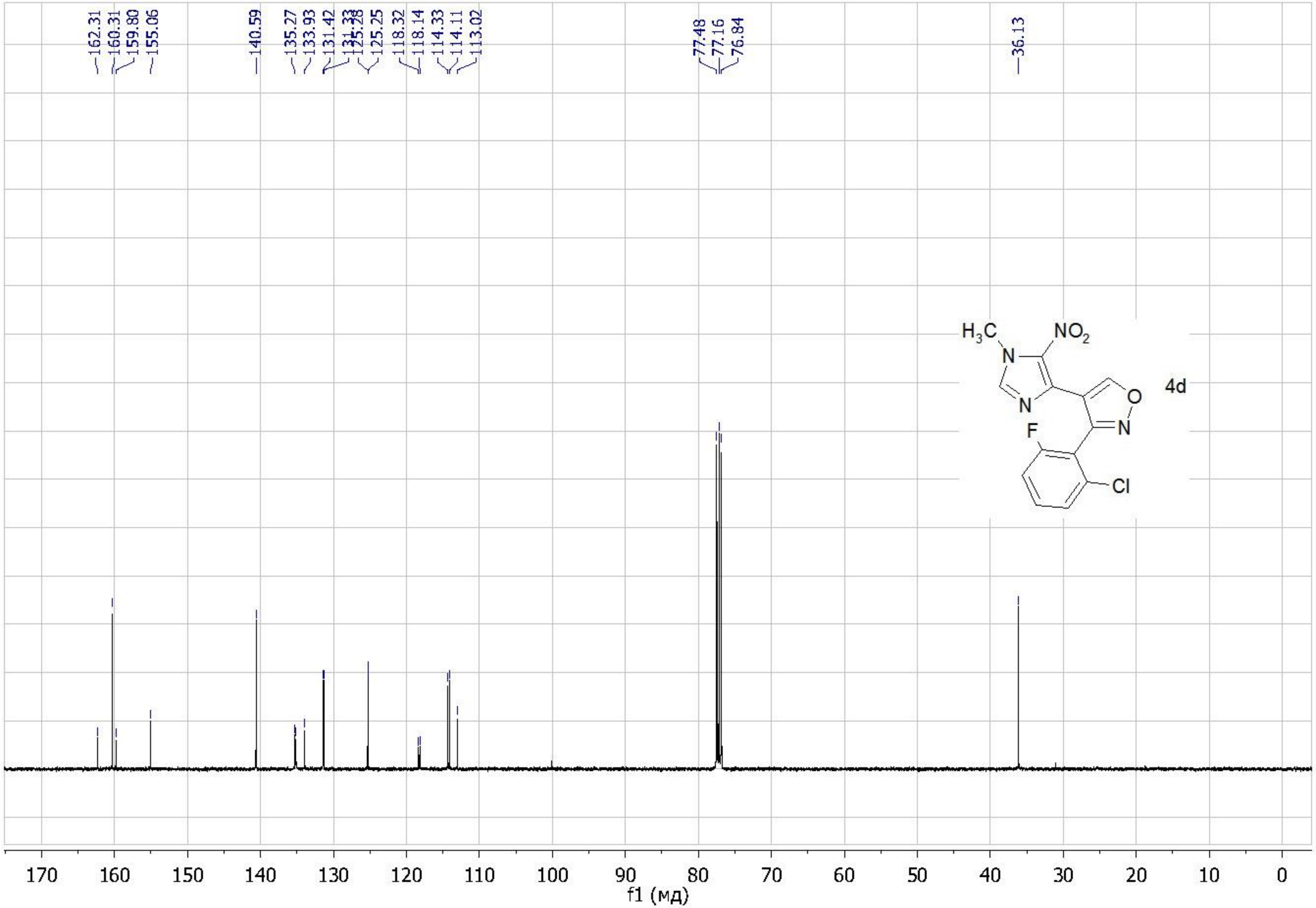
162.31
160.31
159.80
155.06
140.59
135.27
133.93
131.42
131.33
125.28
125.25
118.32
118.14
114.33
114.11
113.02
77.48
77.16
76.84
36.13
170 160 150 140 130 120 110 100 90 80 70 60 50 40 30 20 10 0
f1 (мд)
H3C
NO2
N
N
O
N
F
Cl
4d

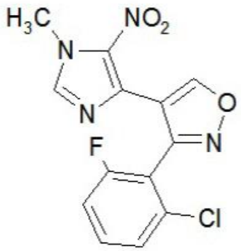

H3C
NO2
N
N
O
N
F
Cl

4d

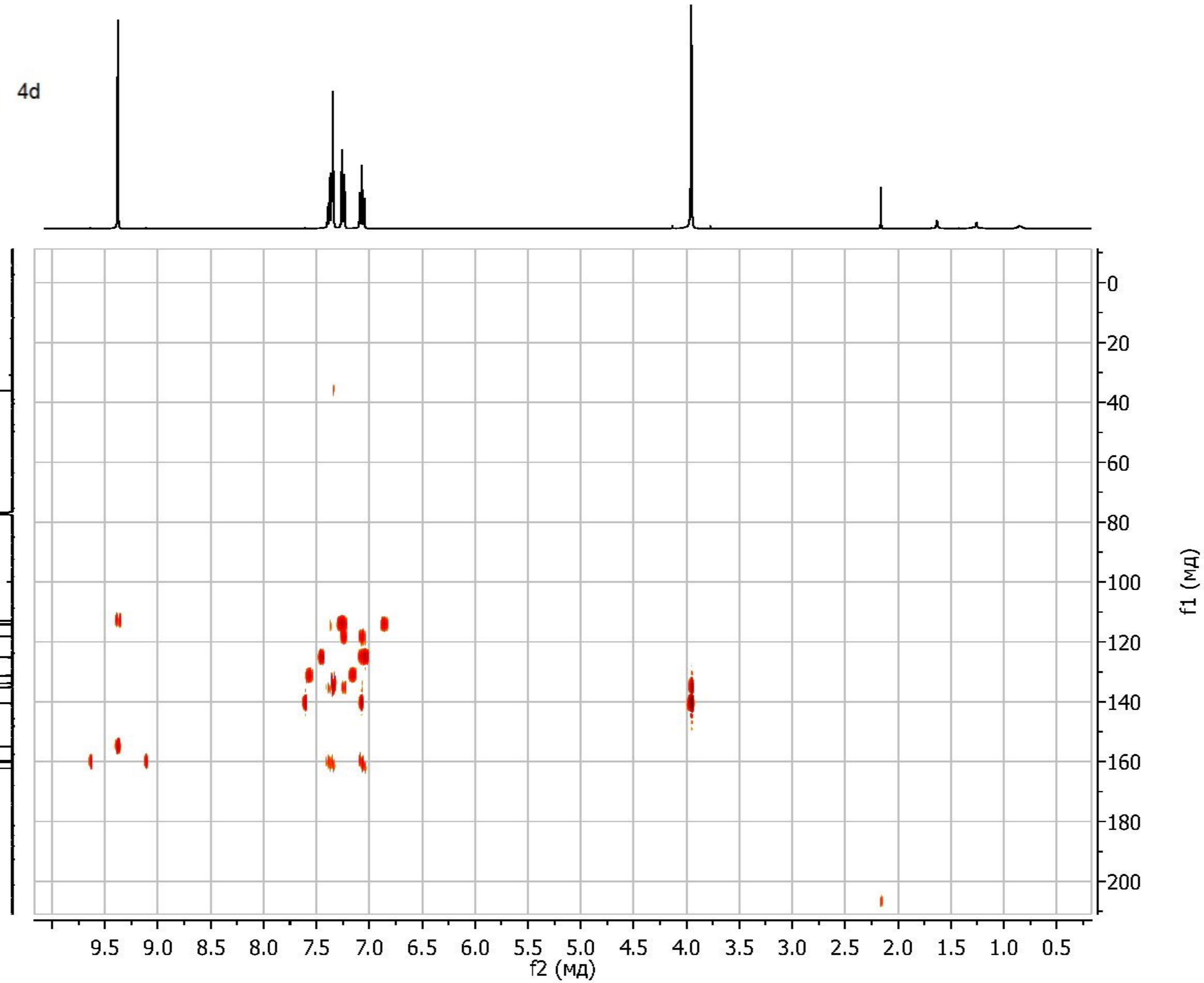

9.5 9.0 8.5 8.0 7.5 7.0 6.5 6.0 5.5 5.0 4.5 4.0 3.5 3.0 2.5 2.0 1.5 1.0 0.5
f2 (мд)
0 20 40 60 80 100 120 140 160 180 200
f1 (мд)

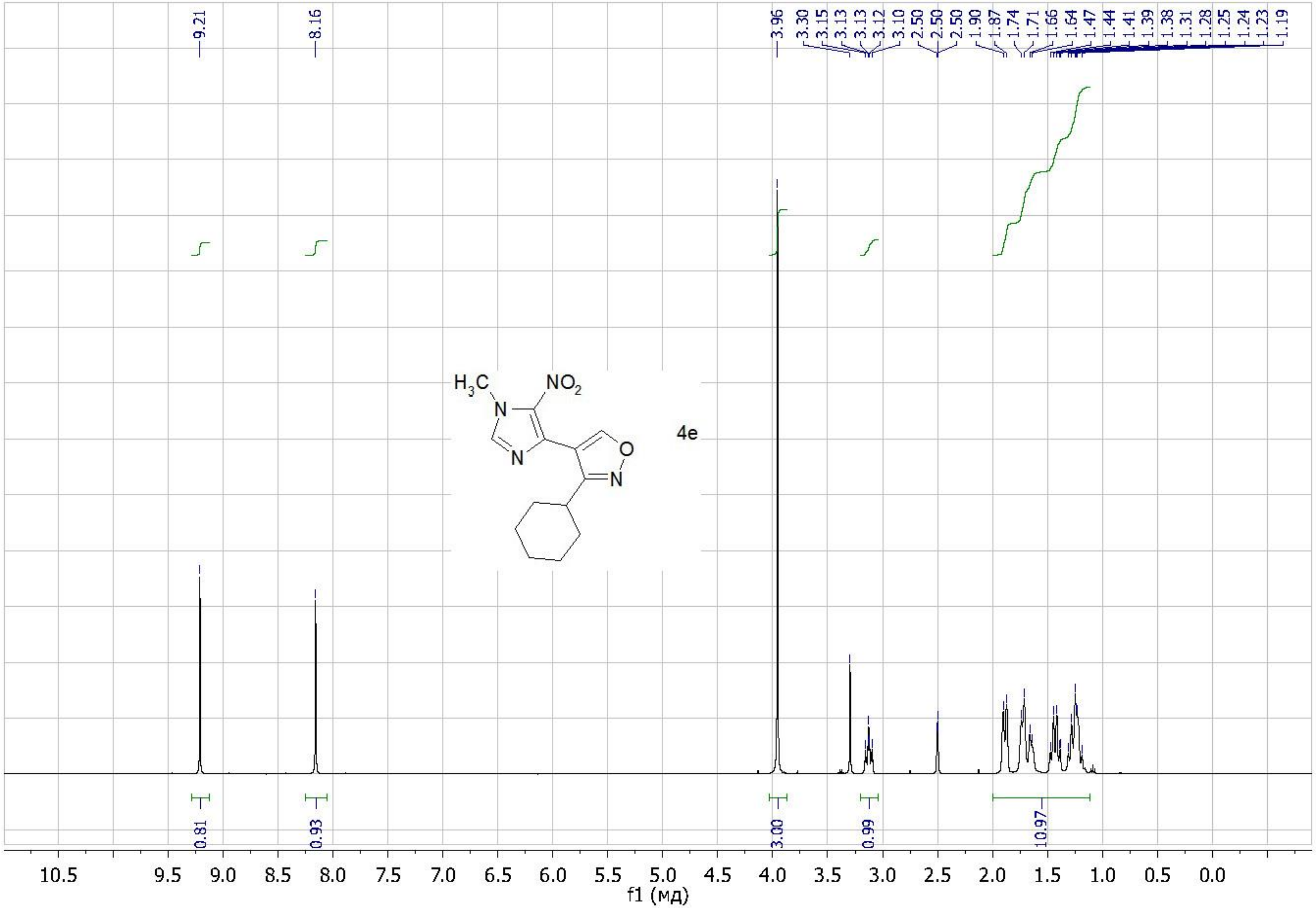
9.21
8.16
3.96
3.30
3.15
3.13
3.13
3.12
3.10
2.50
2.50
2.50
1.90
1.87
1.74
1.71
1.66
1.64
1.47
1.44
1.41
1.39
1.38
1.31
1.28
1.25
1.24
1.23
1.19
H3C
NO2
N
N
O
N
4e
0.81
0.93
3.00
0.99
10.97
10.5 9.5 9.0 8.5 8.0 7.5 7.0 6.5 6.0 5.5 5.0 4.5 4.0 3.5 3.0 2.5 2.0 1.5 1.0 0.5 0.0
f1 (мд)

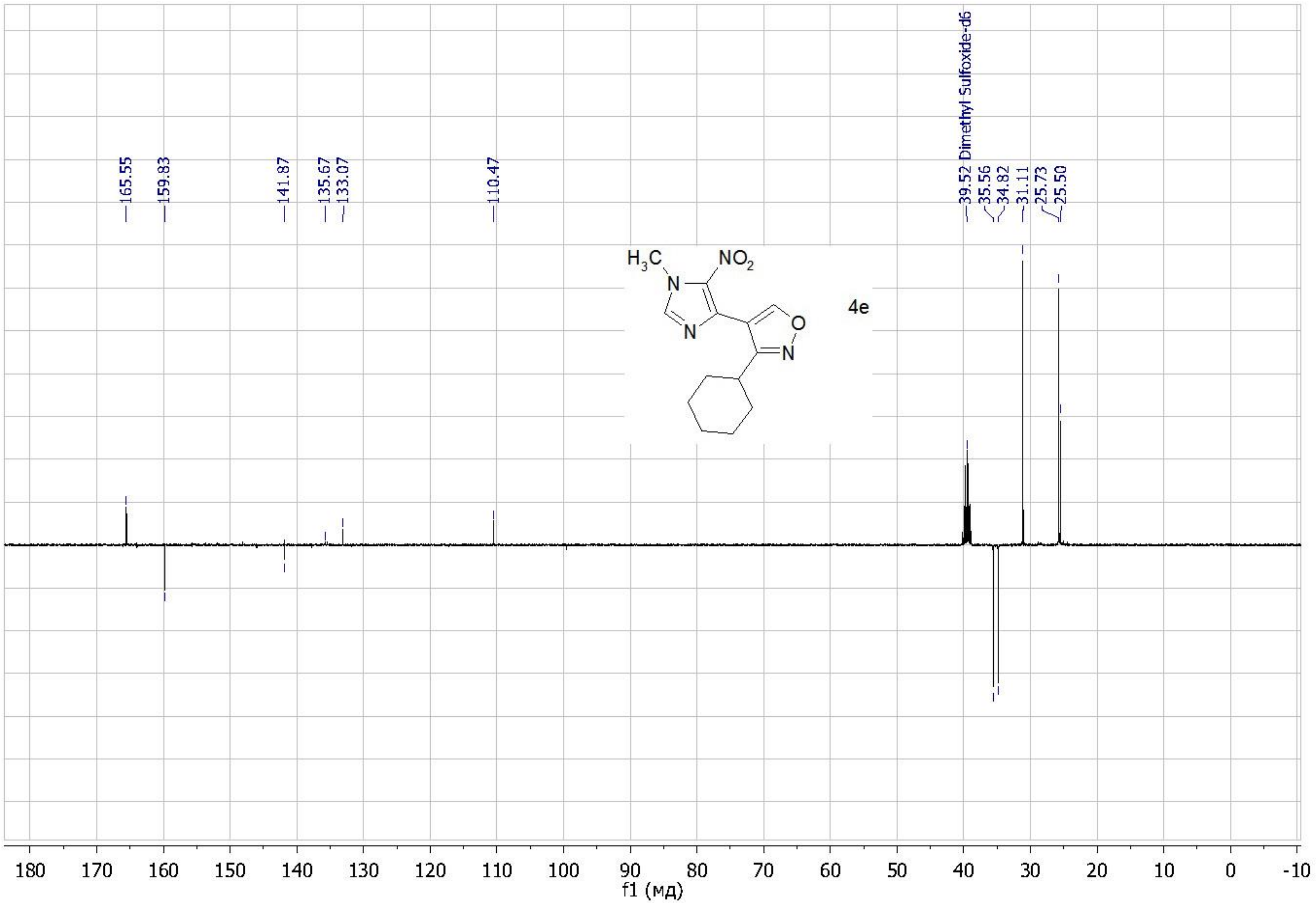

165.55
159.83
141.87
135.67
133.07
110.47
39.52 Dimethyl Sulfoxide-d6
35.56
34.82
31.11
25.73
25.50
4e
180 170 160 150 140 130 120 110 100 90 80 70 60 50 40 30 20 10 0 -10
f1 (мд)

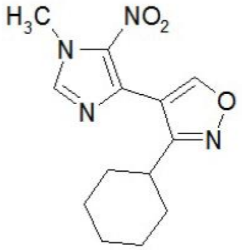

H_3C
NO_2
N
N
O
N

4e

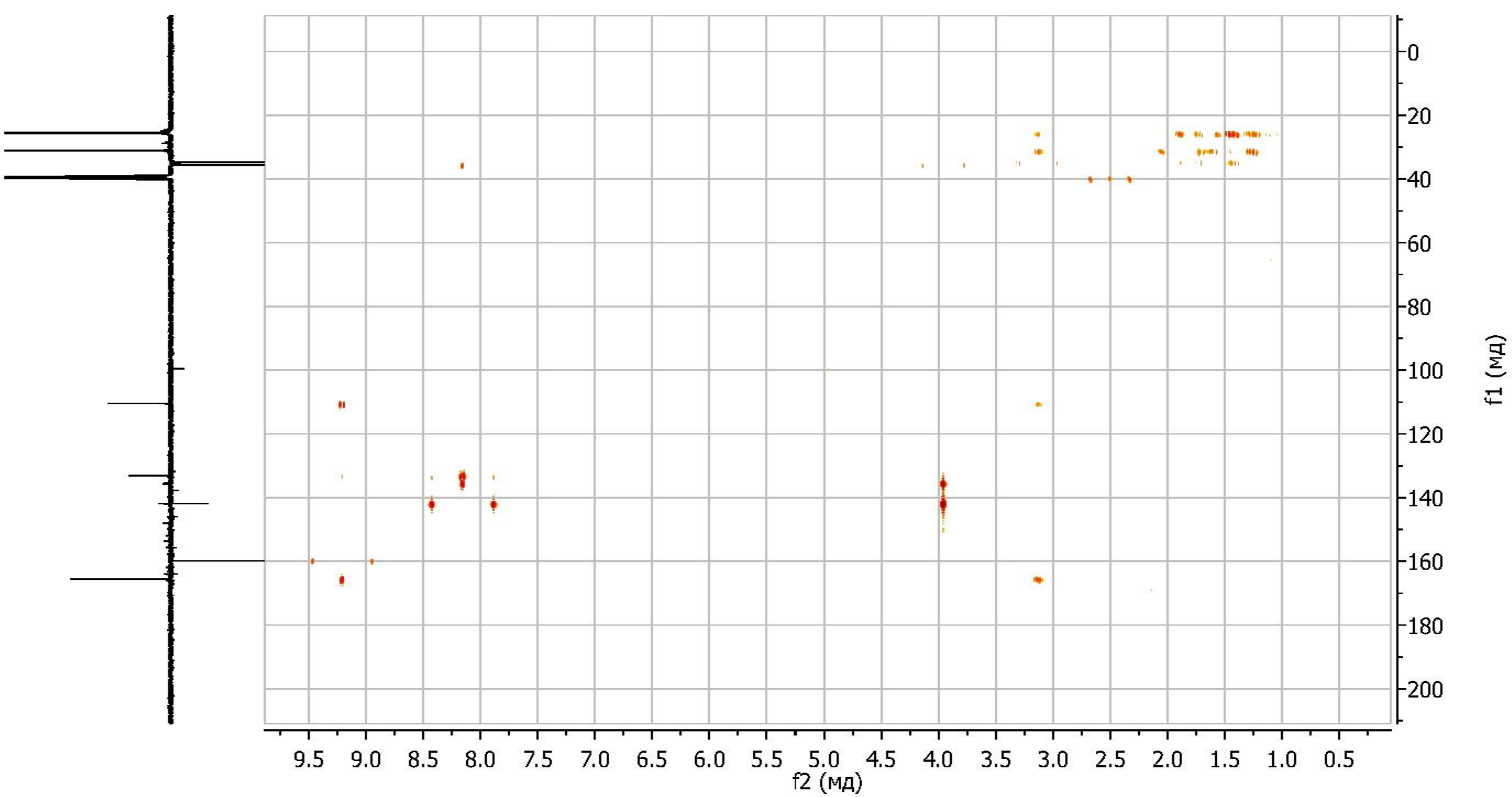

f2 (мд)
f1 (мд)
9.5 9.0 8.5 8.0 7.5 7.0 6.5 6.0 5.5 5.0 4.5 4.0 3.5 3.0 2.5 2.0 1.5 1.0 0.5
0 20 40 60 80 100 120 140 160 180 200

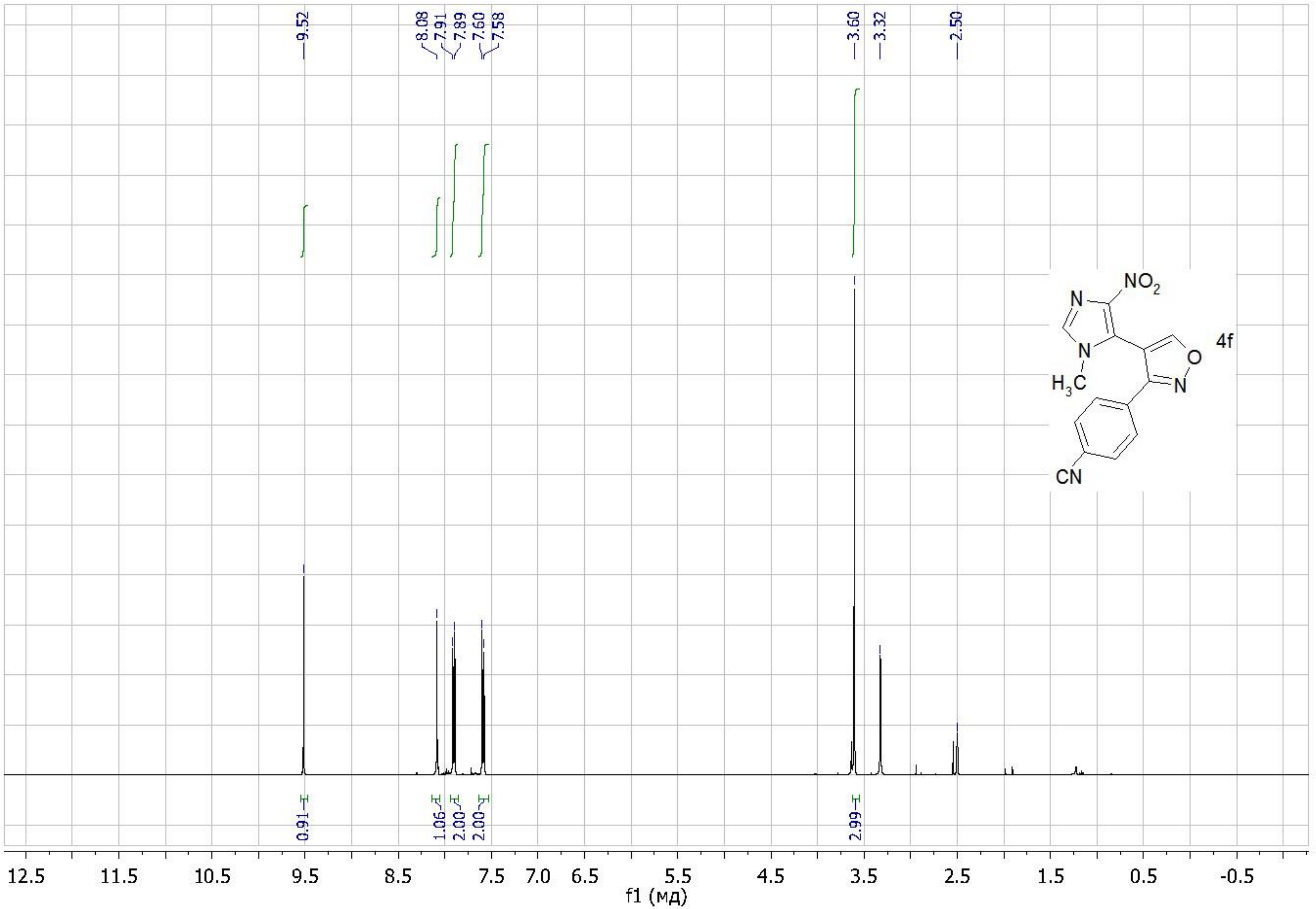
9.52
8.08
7.91
7.89
7.60
7.58
3.60
3.32
2.50
0.91
1.06
2.00
2.00
2.99
12.5
11.5
10.5
9.5
8.5
7.5
7.0
6.5
5.5
4.5
3.5
2.5
1.5
0.5
-0.5
f1 (мд)
NO2
N
N
H3C
O
N
CN
4f

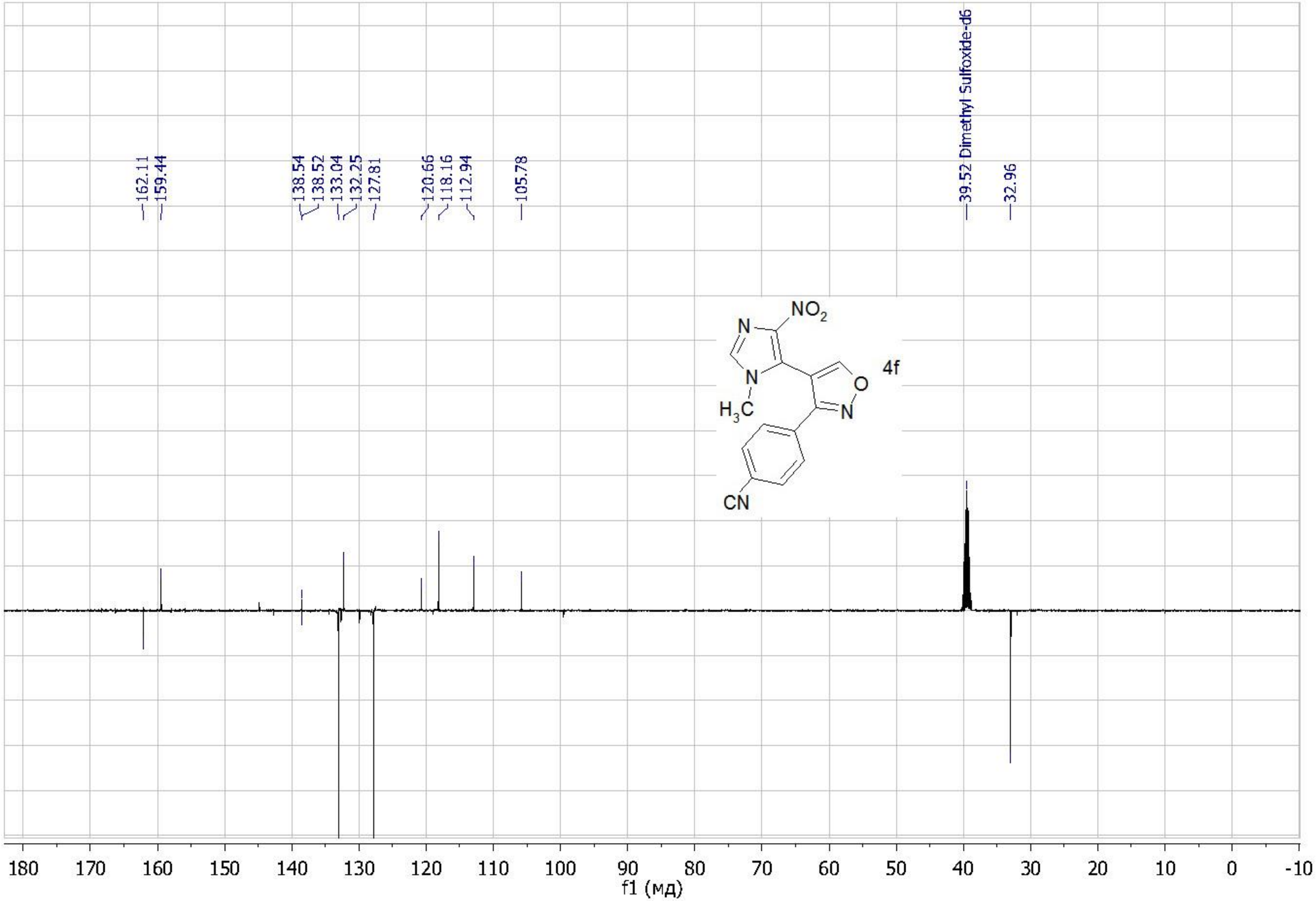
162.11
159.44
138.54
138.52
133.04
132.25
127.81
120.66
118.16
112.94
105.78
39.52 Dimethyl Sulfoxide-d6
32.96
NO_2
N
N
H_3C
O
N
CN
4f
180 170 160 150 140 130 120 110 100 90 80 70 60 50 40 30 20 10 0 -10
f1 (мд)

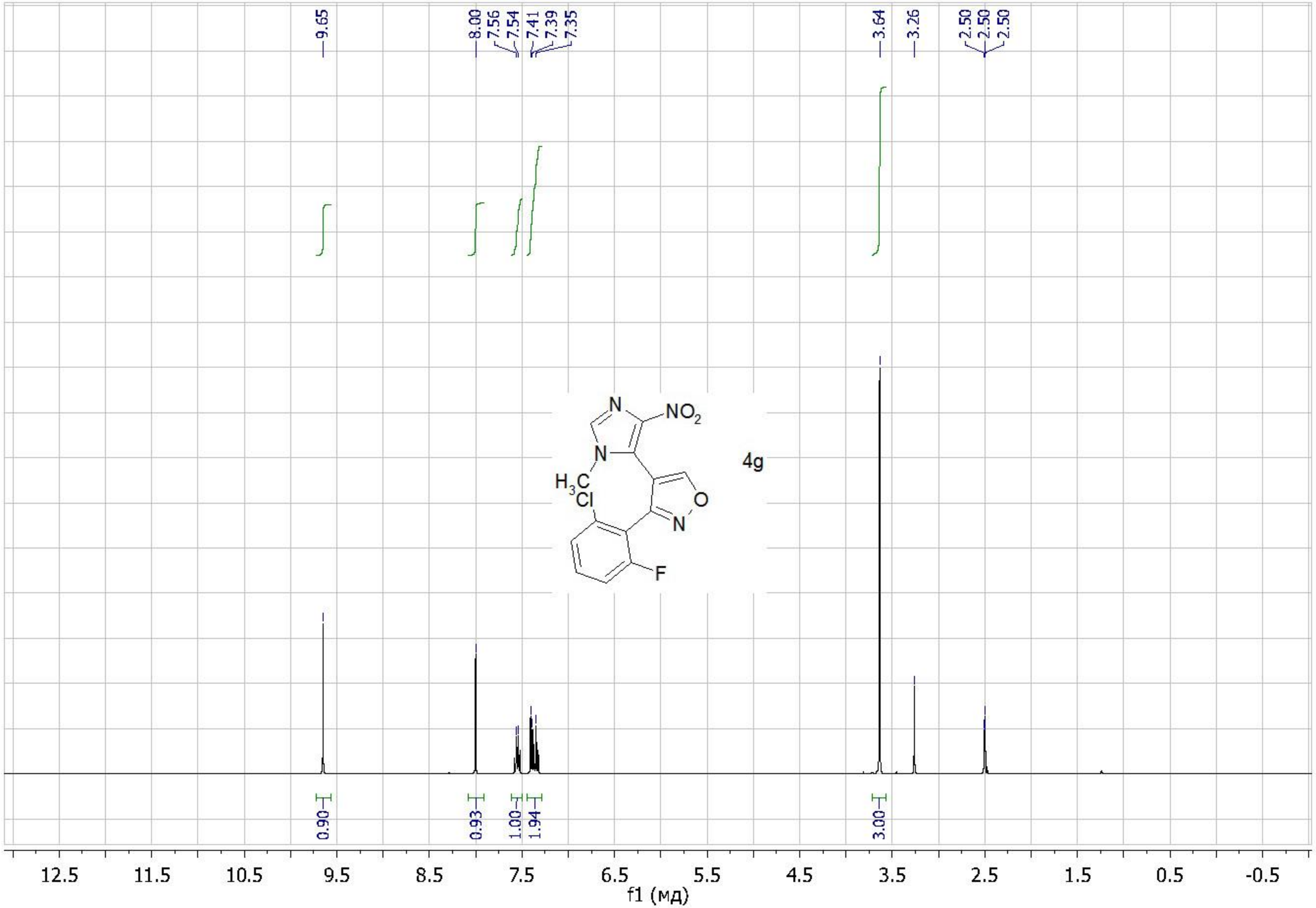
9.65
8.00
7.56
7.54
7.41
7.39
7.35
3.64
3.26
2.50
2.50
2.50
N
NO2
N
4g
H3C
Cl
O
N
F
0.90
0.93
1.00
1.94
3.00
12.5
11.5
10.5
9.5
8.5
7.5
6.5
5.5
4.5
3.5
2.5
1.5
0.5
-0.5
f1 (мд)

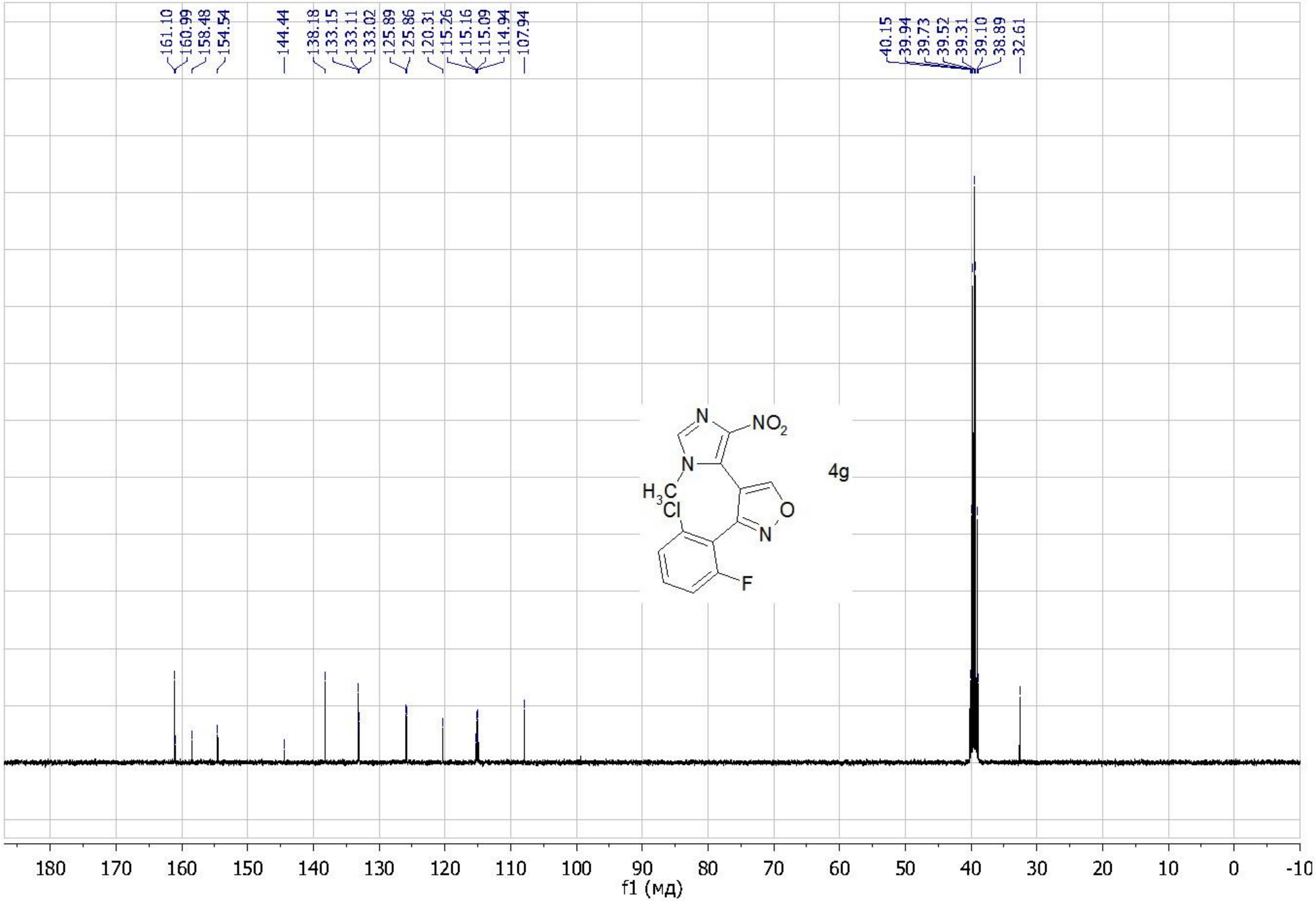

161.10
160.99
158.48
154.54
144.44
138.18
133.15
133.11
133.02
125.89
125.86
120.31
115.26
115.16
115.09
114.94
107.94
40.15
39.94
39.73
39.52
39.31
39.10
38.89
32.61
N
NO2
N
H3C
Cl
O
N
F
4g
180
170
160
150
140
130
120
110
100
90
80
70
60
50
40
30
20
10
0
-10
f1 (мд)

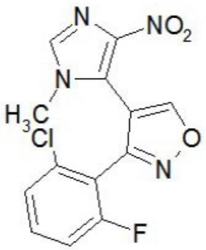

4g

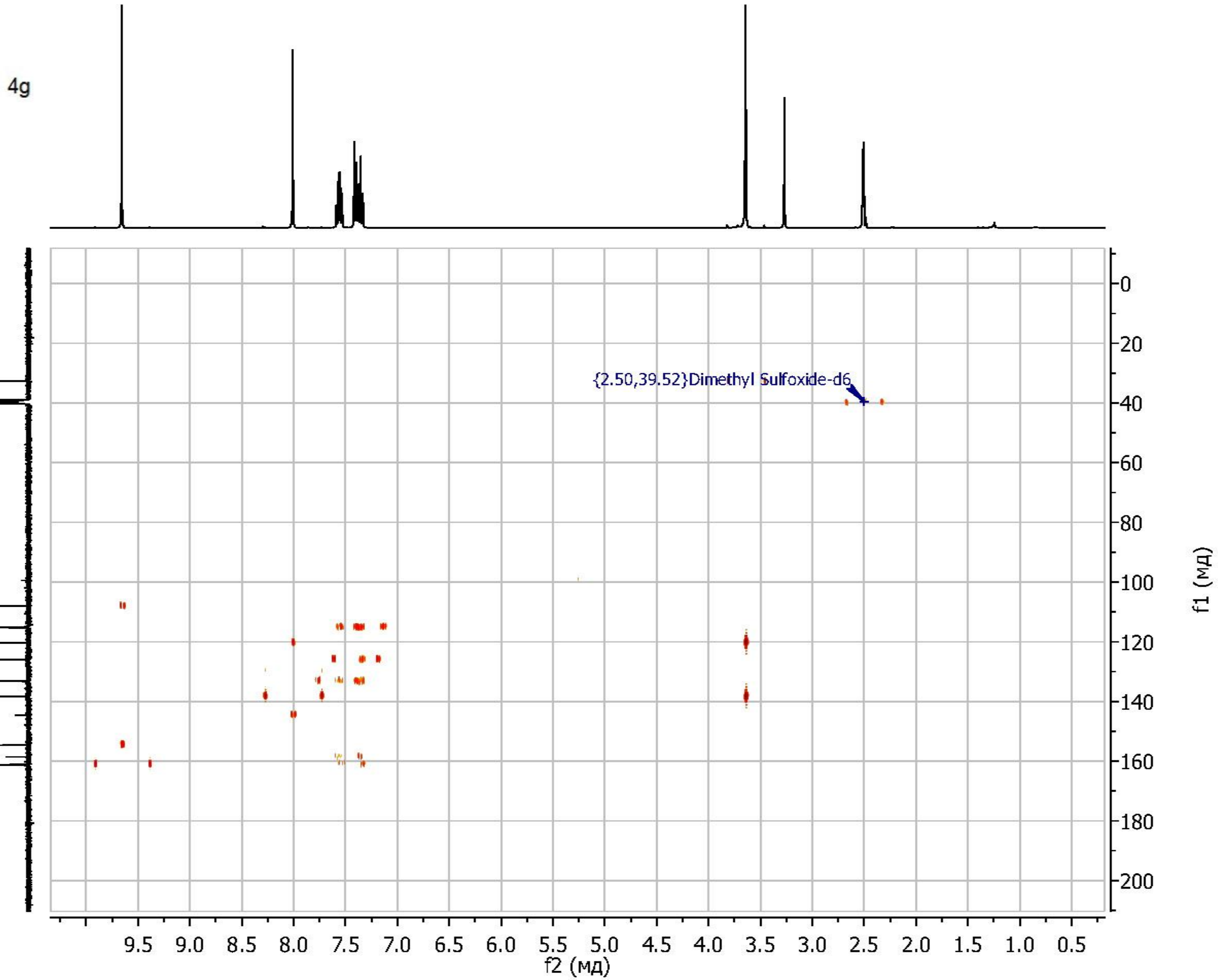

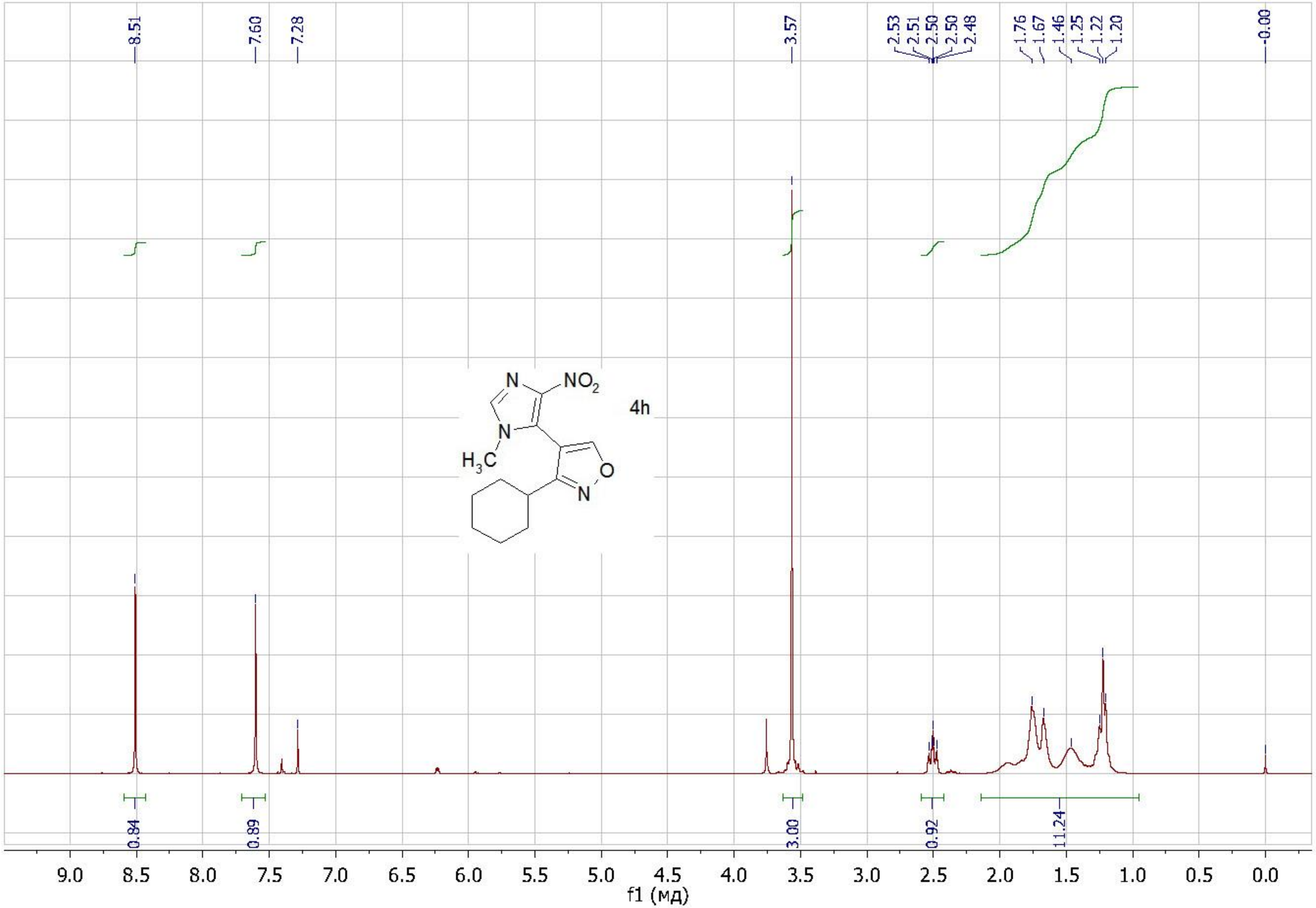
8.51
7.60
7.28
3.57
2.53
2.51
2.50
2.50
2.48
1.76
1.67
1.46
1.25
1.22
1.20
-0.00
NO2
N
4h
N
H3C
O
N
0.84
0.89
3.00
0.92
11.24
9.0
8.5
8.0
7.5
7.0
6.5
6.0
5.5
5.0
4.5
4.0
3.5
3.0
2.5
2.0
1.5
1.0
0.5
0.0
f1 (мд)

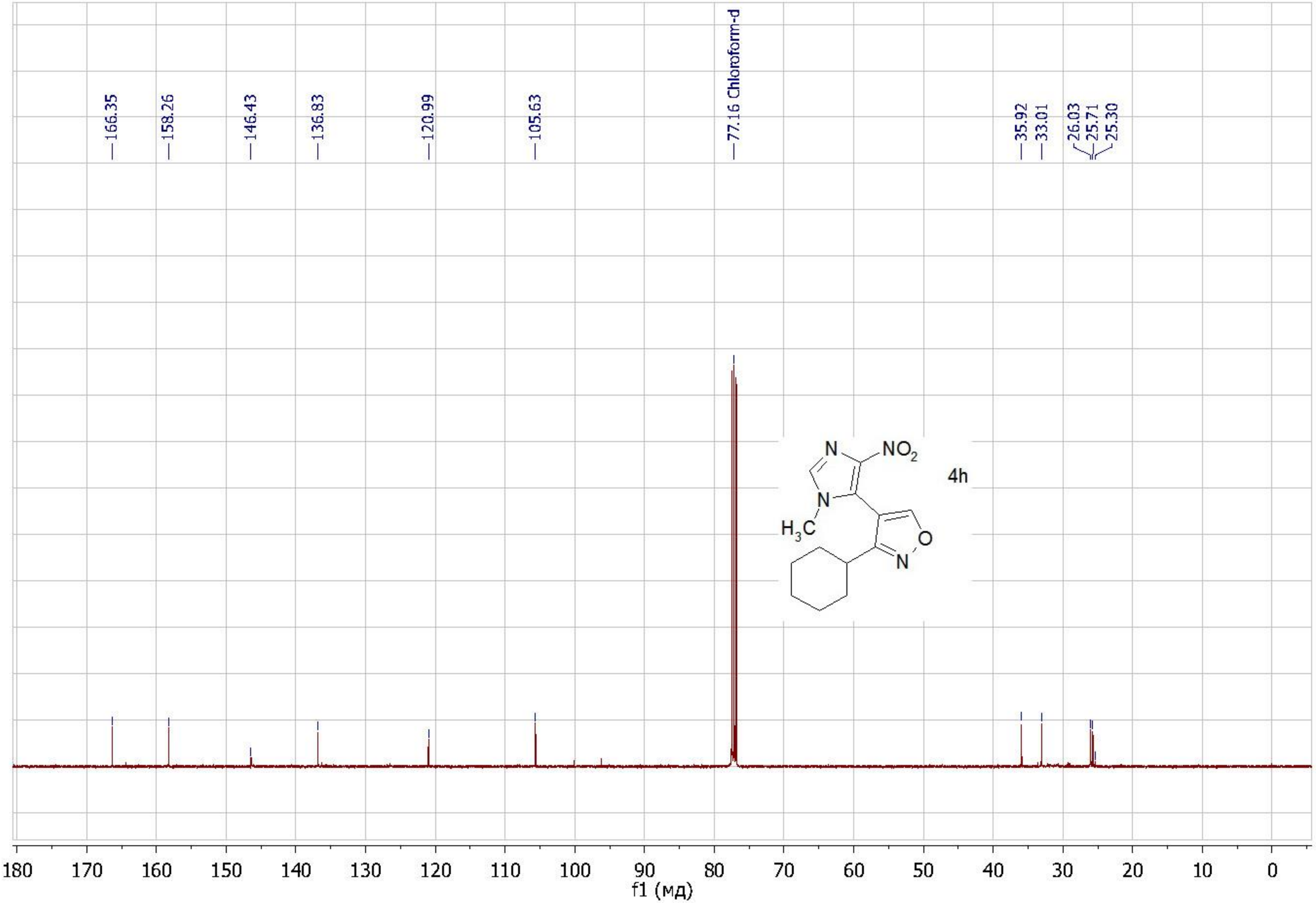
166.35
158.26
146.43
136.83
120.99
105.63
77.16 Chloroform-d
35.92
33.01
26.03
25.71
25.30
NO2
N
N
H3C
O
N
4h
180 170 160 150 140 130 120 110 100 90 80 70 60 50 40 30 20 10 0
f1 (мд)

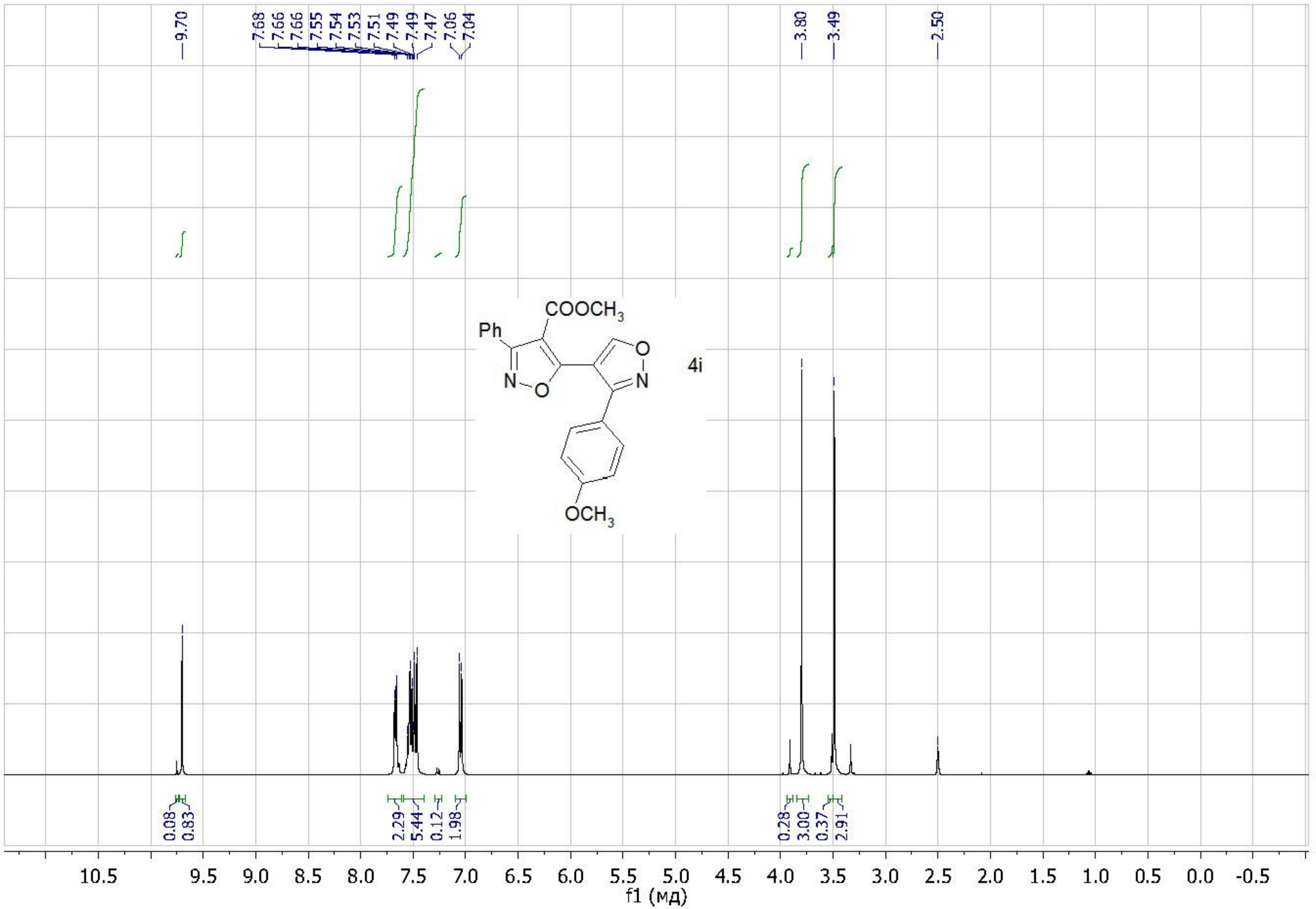
9.70
7.68
7.66
7.66
7.55
7.54
7.53
7.51
7.49
7.49
7.47
7.06
7.04
3.80
3.49
2.50
COOCH3
Ph
N
O
O
N
4i
OCH3
0.08
0.83
2.29
5.44
0.12
1.98
0.28
3.00
0.37
2.91
10.5
9.5
9.0
8.5
8.0
7.5
7.0
6.5
6.0
5.5
5.0
4.5
4.0
3.5
3.0
2.5
2.0
1.5
1.0
0.5
0.0
-0.5
f1 (мд)

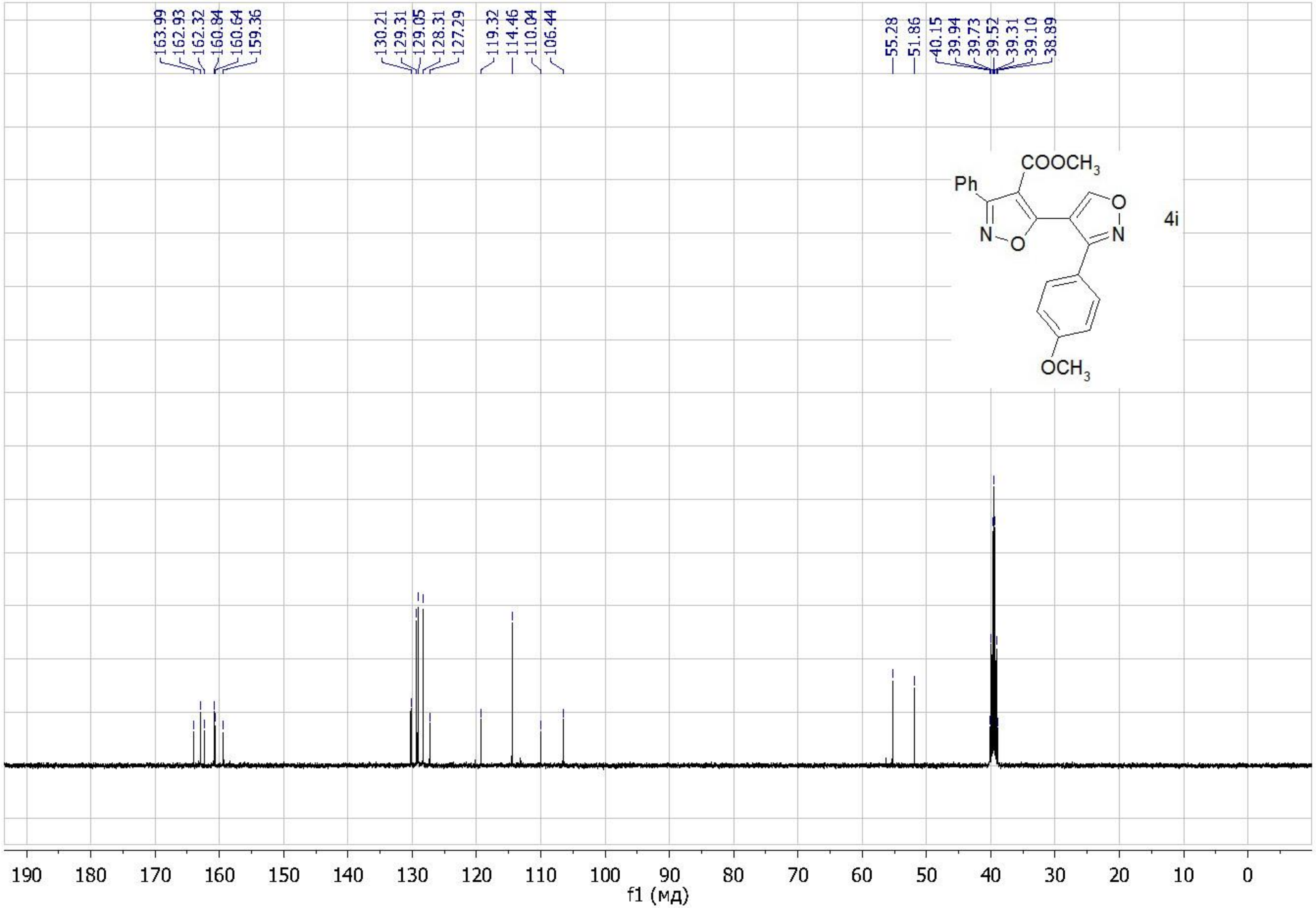
163.99
162.93
162.32
160.84
160.64
159.36
130.21
129.31
129.05
128.31
127.29
119.32
114.46
110.04
106.44
55.28
51.86
40.15
39.94
39.73
39.52
39.31
39.10
38.89
COOCH3
Ph
O
N
N
O
OCH3
4i
190 180 170 160 150 140 130 120 110 100 90 80 70 60 50 40 30 20 10 0
f1 (мд)

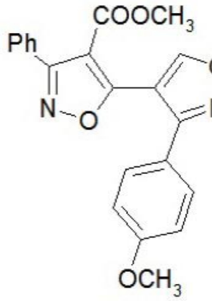

4i

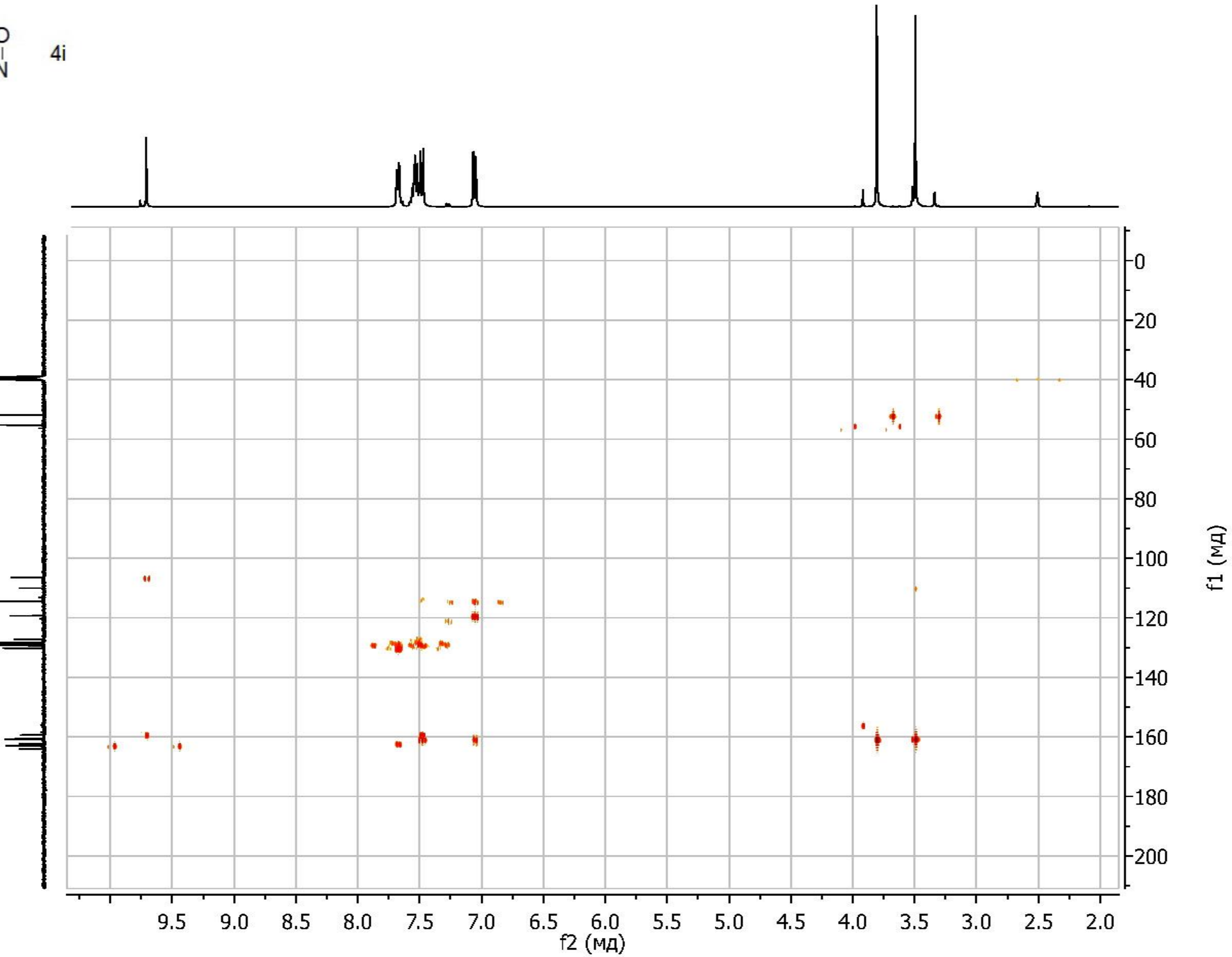

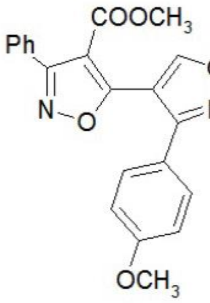

4i

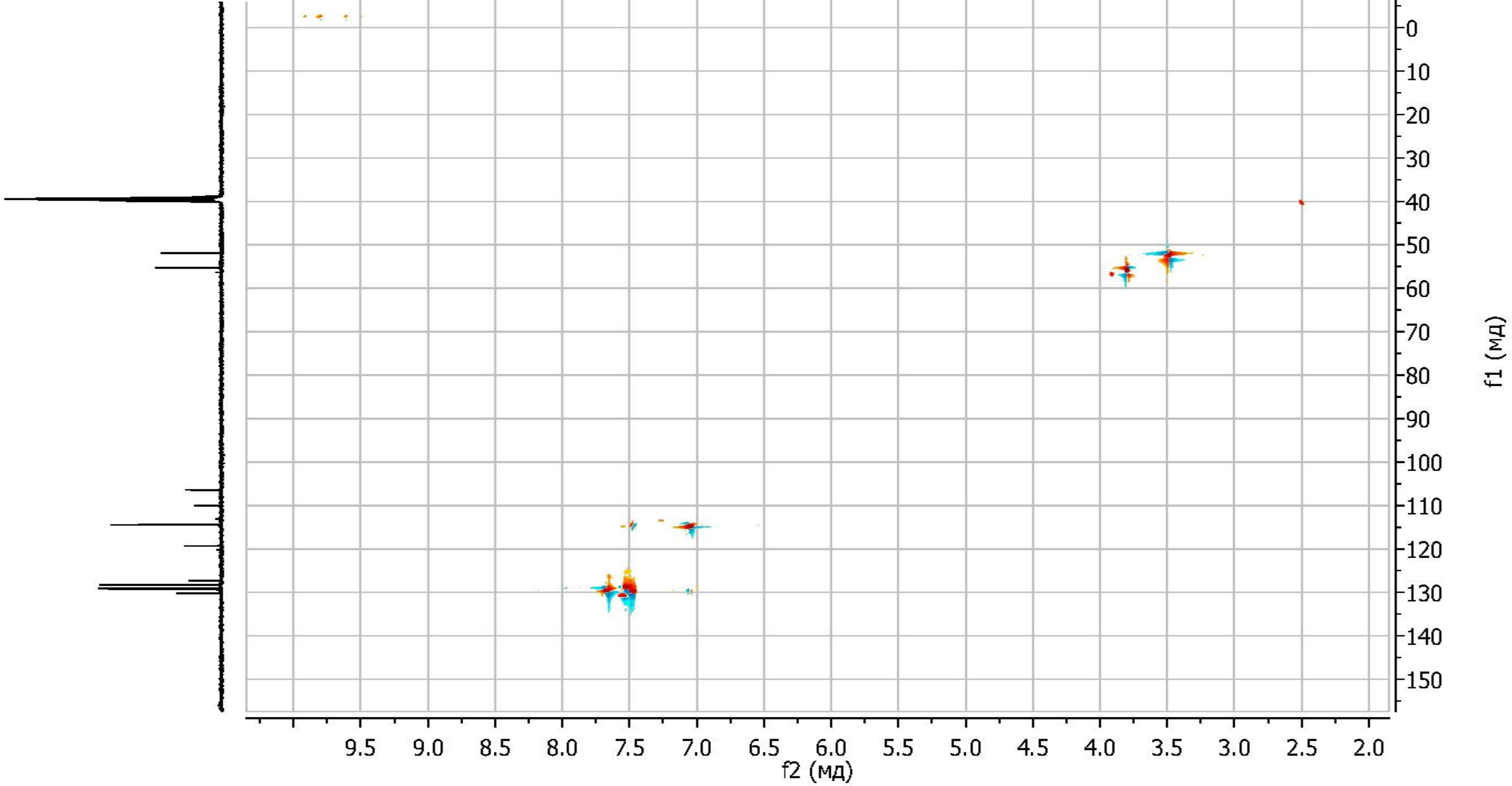

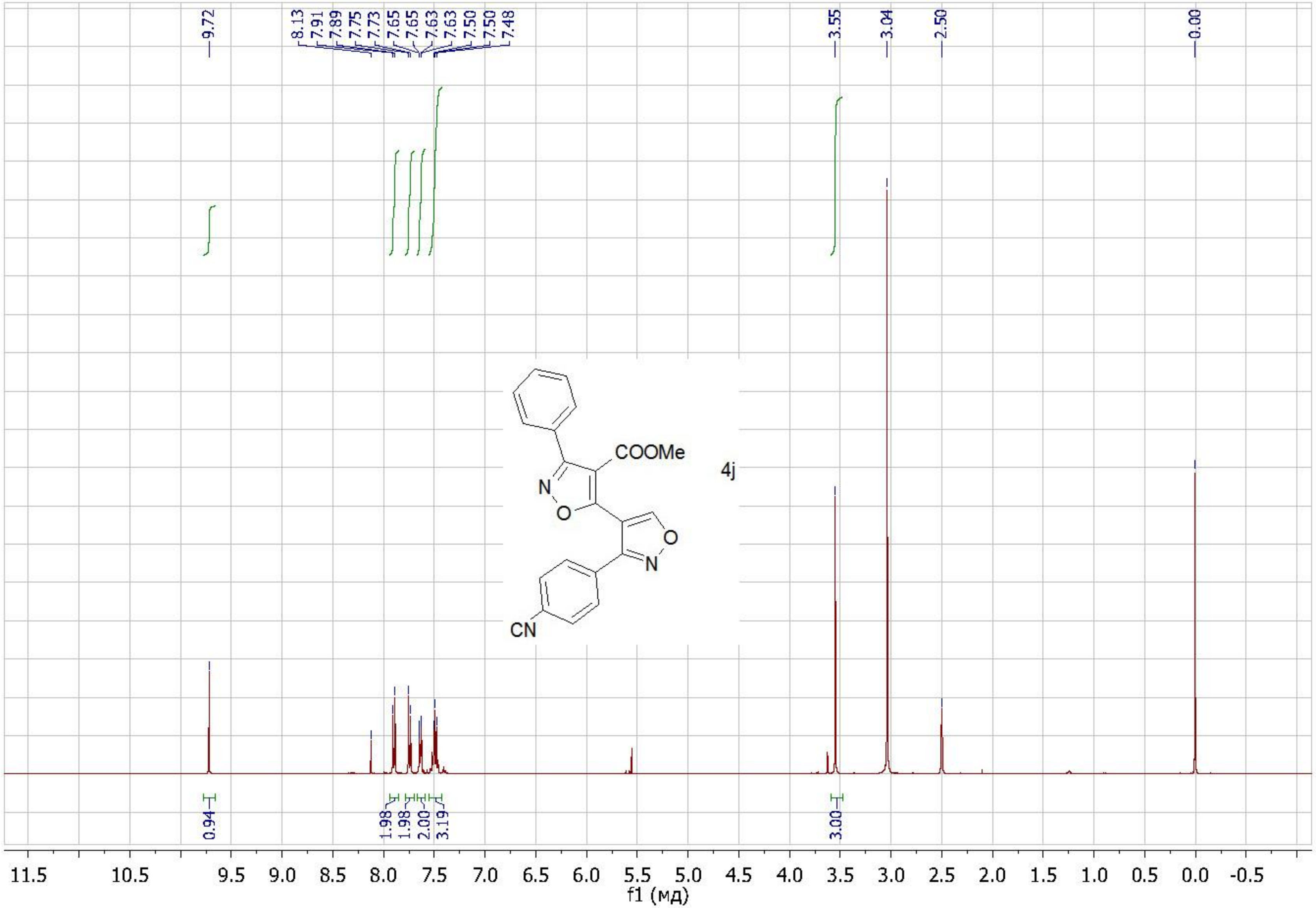
9.72
8.13
7.91
7.89
7.75
7.73
7.65
7.65
7.63
7.63
7.50
7.50
7.48
3.55
3.04
2.50
0.00
COOMe
4j
N
O
N
CN
0.94
1.98
1.98
2.00
3.19
3.00
f1 (мд)

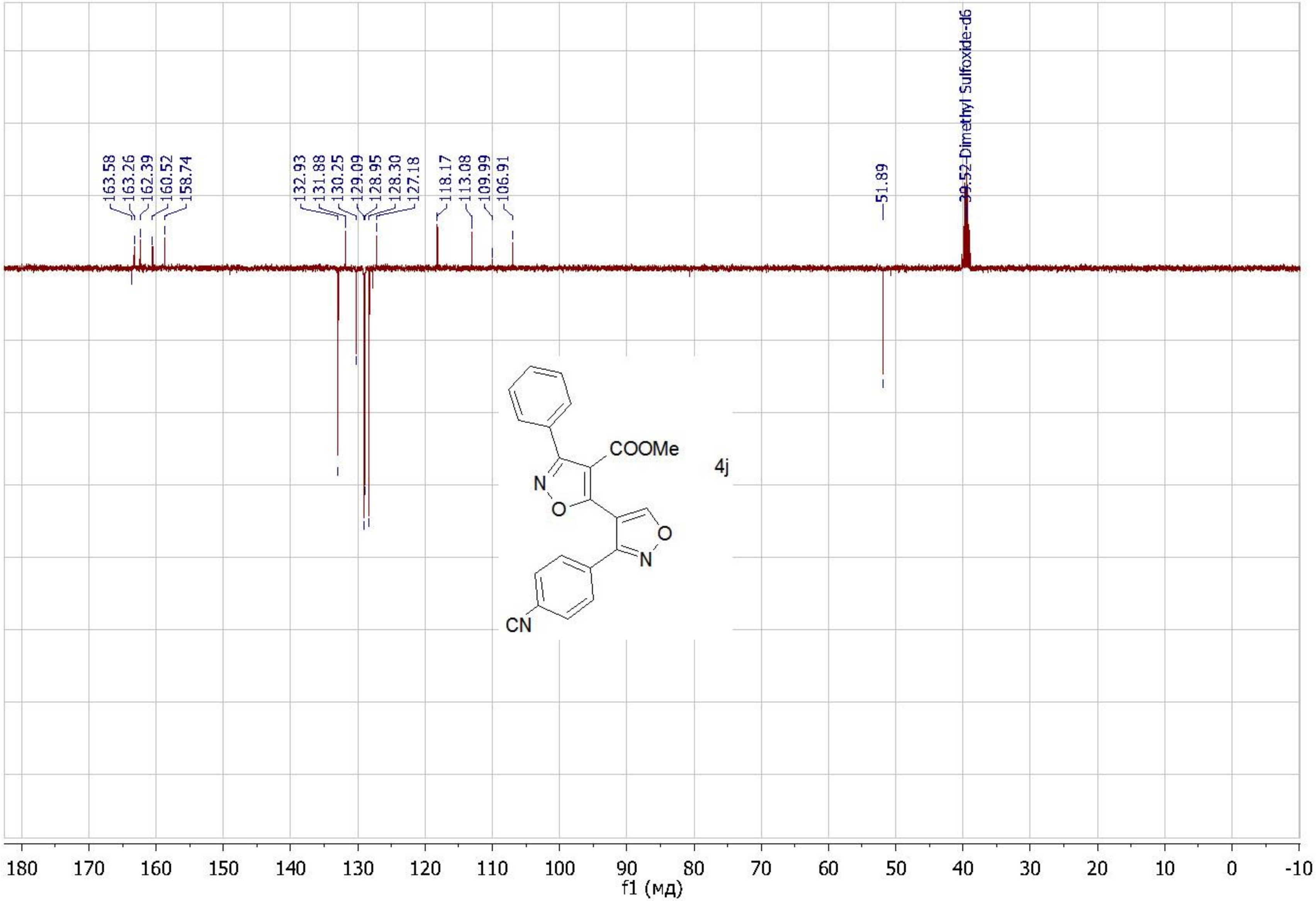
163.58
163.26
162.39
160.52
158.74
132.93
131.88
130.25
129.09
128.95
128.30
127.18
118.17
113.08
109.99
106.91
51.89
39.52 Dimethyl Sulfoxide-d6
COOMe
N
O
N
O
CN
4j
180 170 160 150 140 130 120 110 100 90 80 70 60 50 40 30 20 10 0 -10
f1 (мд)

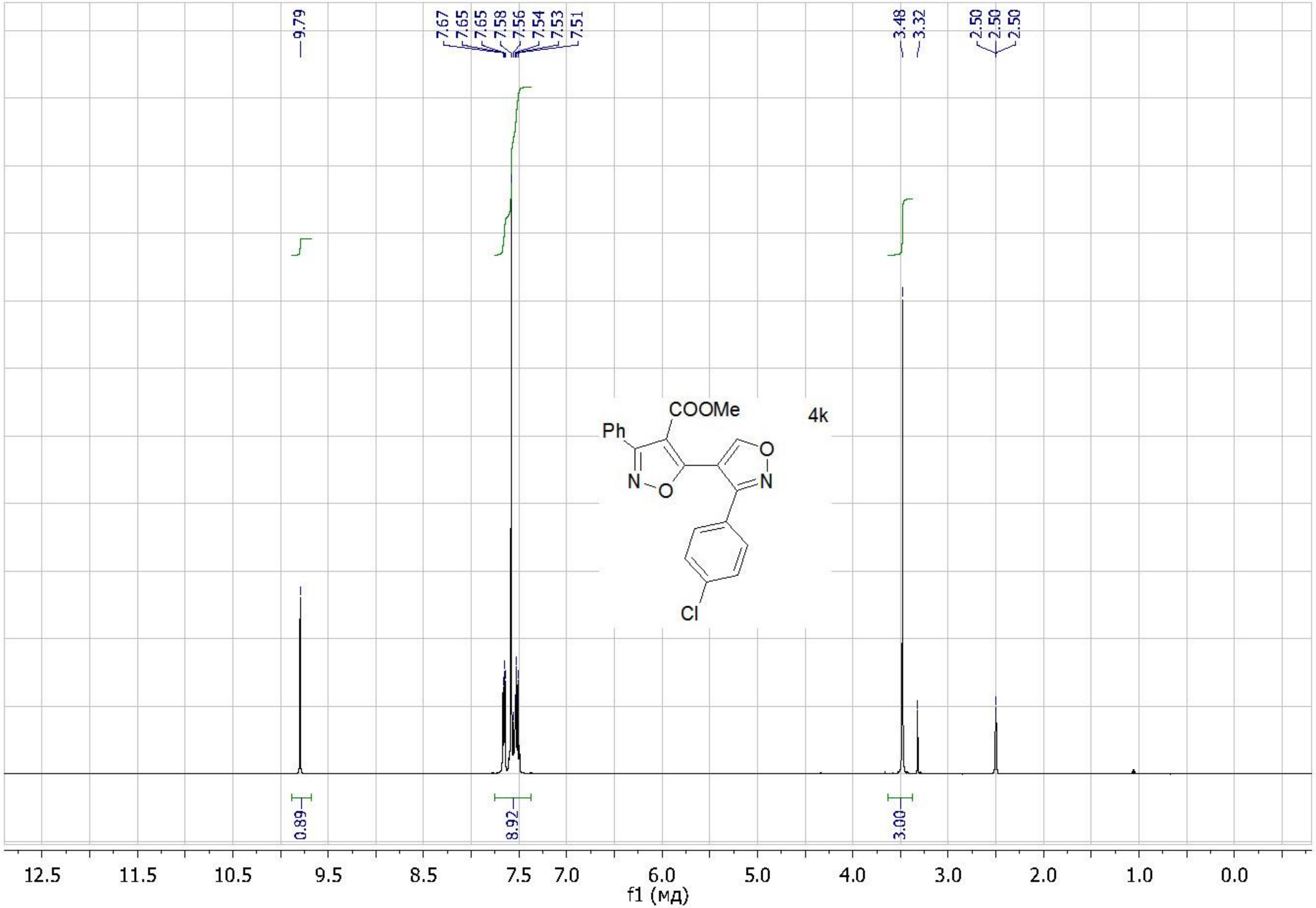

9.79
7.67
7.65
7.65
7.58
7.56
7.54
7.53
7.51
3.48
3.32
2.50
2.50
2.50
COOMe
4k
Ph
N
O
O
N
Cl
0.89
8.92
3.00
12.5
11.5
10.5
9.5
8.5
7.5
7.0
6.0
5.0
4.0
3.0
2.0
1.0
0.0
f1 (мд)

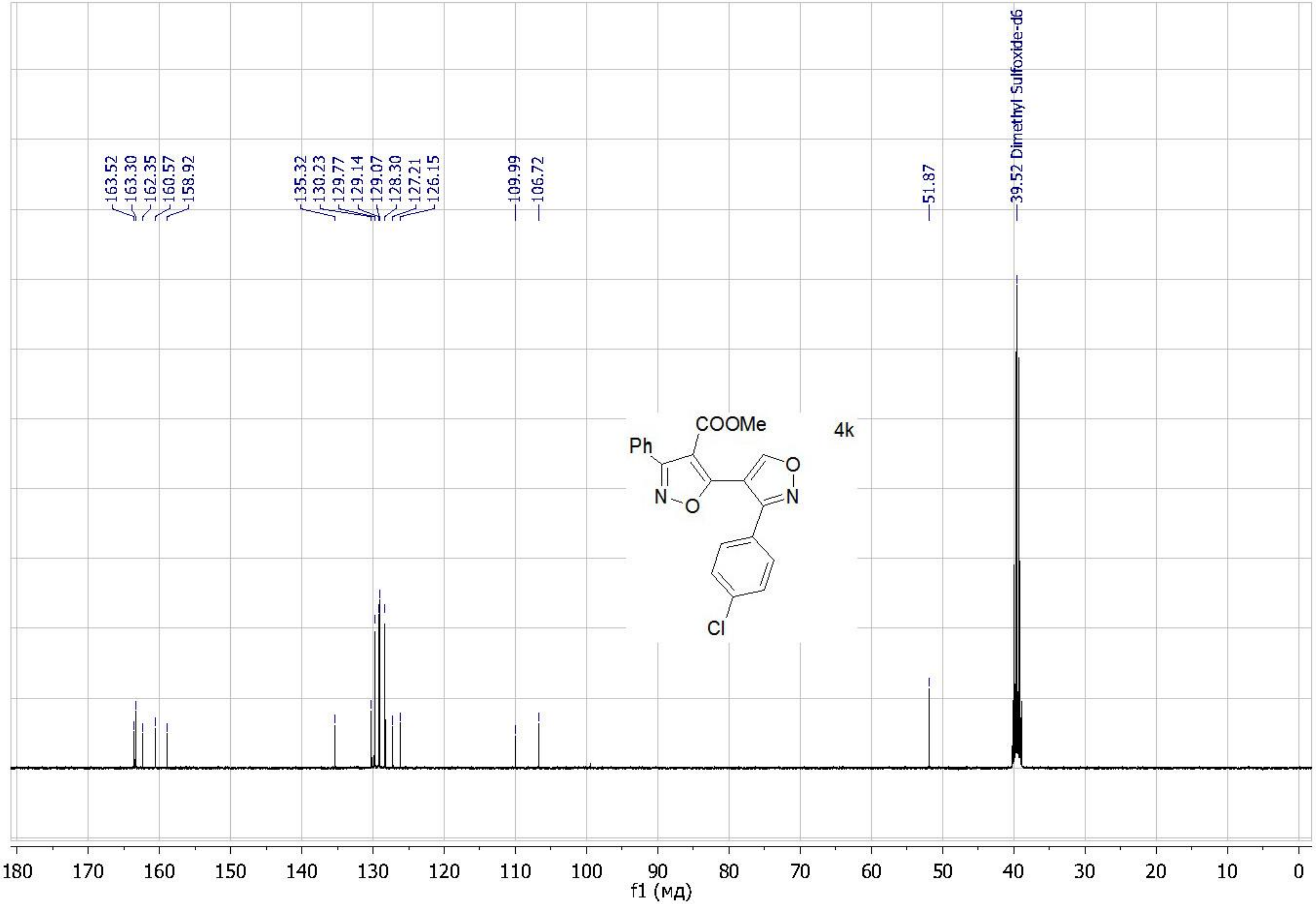
163.52
163.30
162.35
160.57
158.92
135.32
130.23
129.77
129.14
129.07
128.30
127.21
126.15
109.99
106.72
51.87
39.52 Dimethyl Sulfoxide-d6
COOMe
Ph
N
O
N
O
Cl
4k
180 170 160 150 140 130 120 110 100 90 80 70 60 50 40 30 20 10 0
f1 (мд)

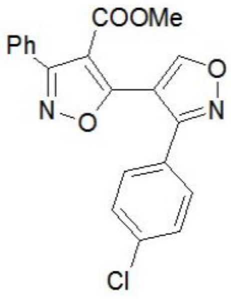

4k

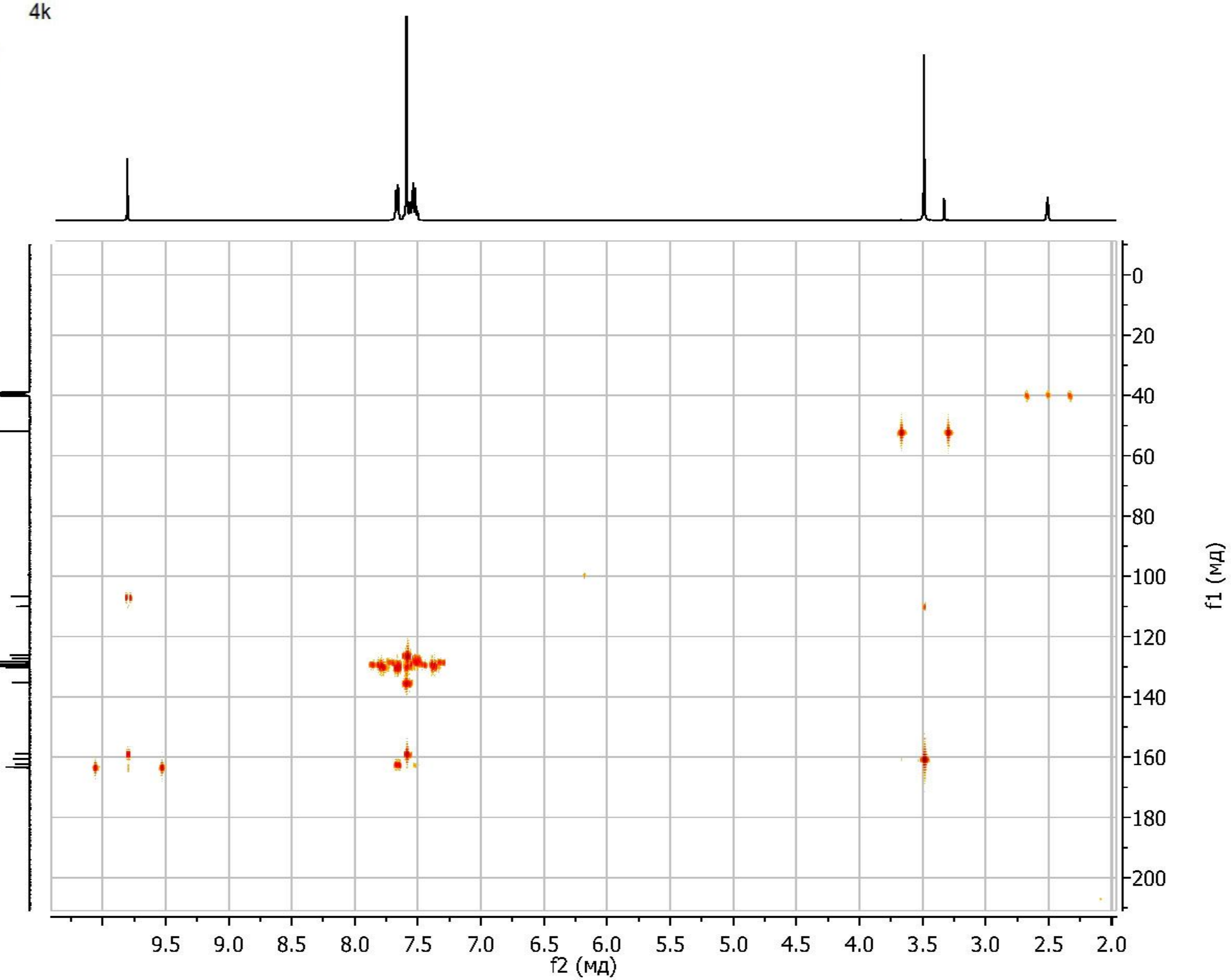

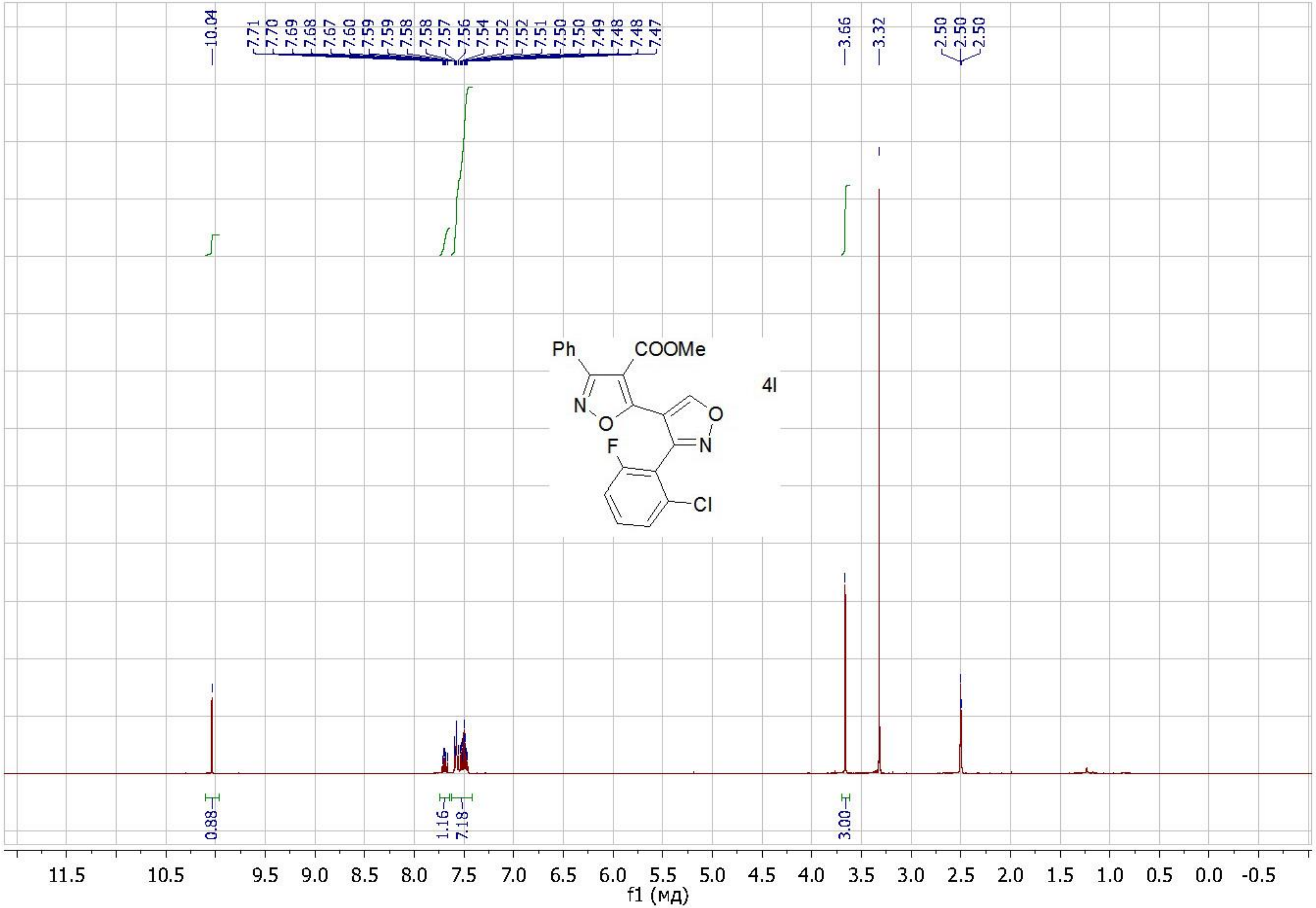

10.04
7.71 7.70 7.69 7.68 7.67 7.60 7.59 7.59 7.58 7.58 7.57 7.56 7.54 7.52 7.52 7.51 7.50 7.50 7.49 7.48 7.48 7.47
3.66
3.32
2.50 2.50 2.50
Ph
COOMe
4l
N
O
F
Cl
0.88
1.16
7.18
3.00
11.5 10.5 9.5 9.0 8.5 8.0 7.5 7.0 6.5 6.0 5.5 5.0 4.5 4.0 3.5 3.0 2.5 2.0 1.5 1.0 0.5 0.0 -0.5
f1 (мд)

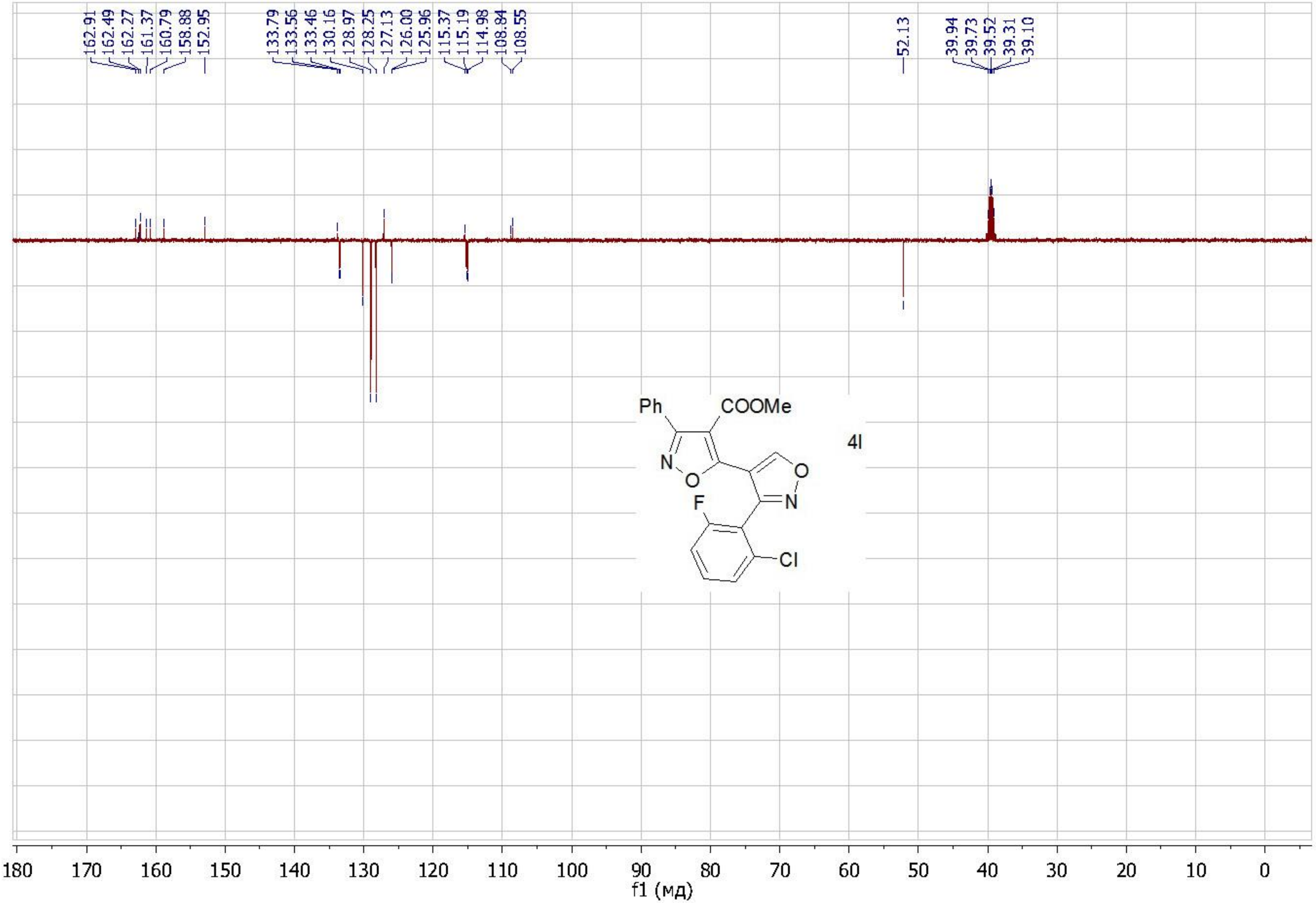
162.91
162.49
162.27
161.37
160.79
158.88
152.95
133.79
133.56
133.46
130.16
128.97
128.25
127.13
126.00
125.96
115.37
115.19
114.98
108.84
108.55
52.13
39.94
39.73
39.52
39.31
39.10
Ph
COOMe
N
O
O
N
F
Cl
4l
180 170 160 150 140 130 120 110 100 90 80 70 60 50 40 30 20 10 0
f1 (мд)

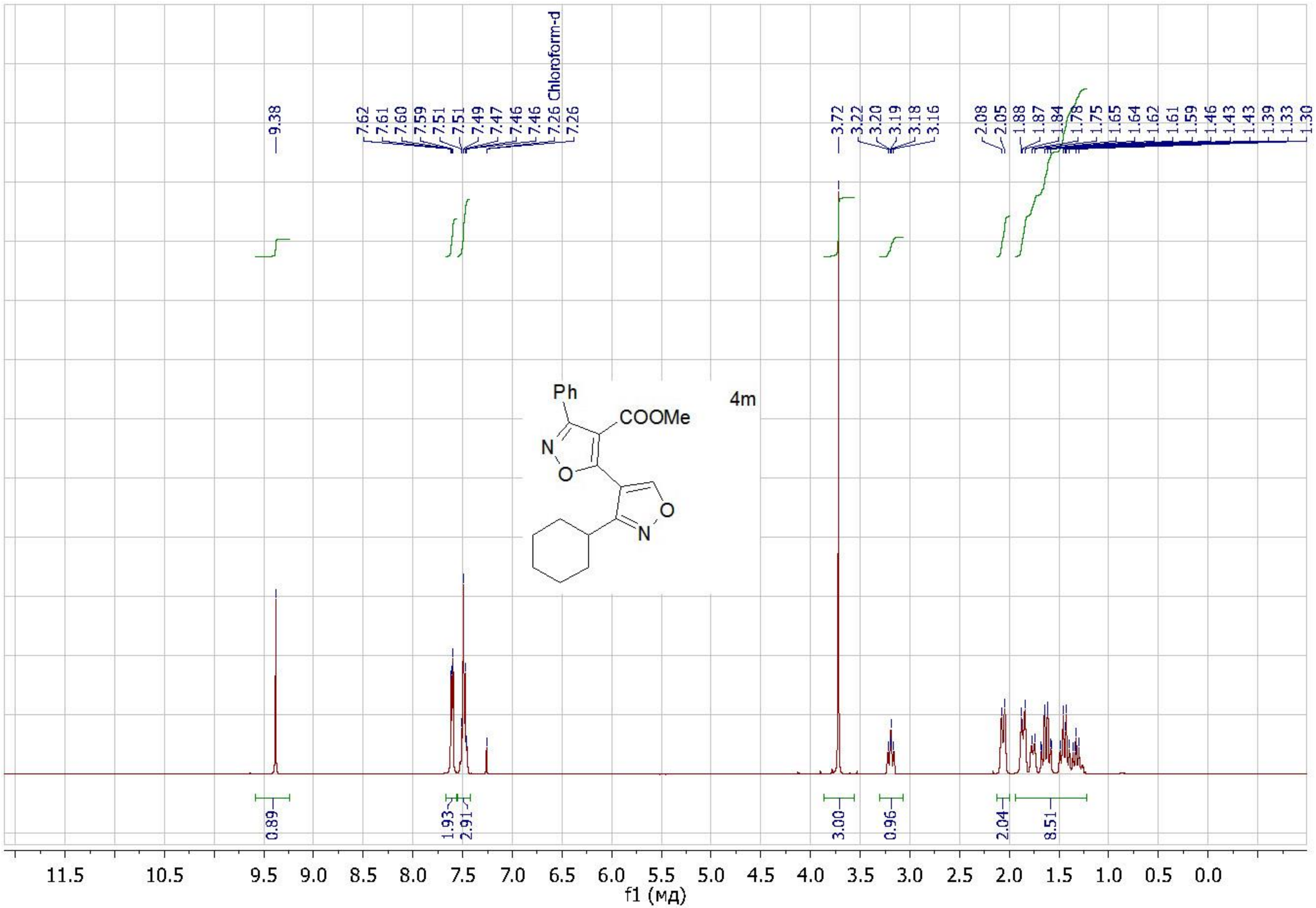
4m
Ph
COOMe
N
O
O
N
Chloroform-d
9.38
7.62
7.61
7.60
7.59
7.51
7.51
7.49
7.47
7.46
7.46
7.26
7.26
3.72
3.22
3.20
3.19
3.18
3.16
2.08
2.05
1.88
1.87
1.84
1.78
1.75
1.65
1.64
1.62
1.61
1.59
1.46
1.43
1.43
1.39
1.33
1.30
0.89
1.93
2.91
3.00
0.96
2.04
8.51
11.5
10.5
9.5
9.0
8.5
8.0
7.5
7.0
6.5
6.0
5.5
5.0
4.5
4.0
3.5
3.0
2.5
2.0
1.5
1.0
0.5
0.0
f1 (мд)

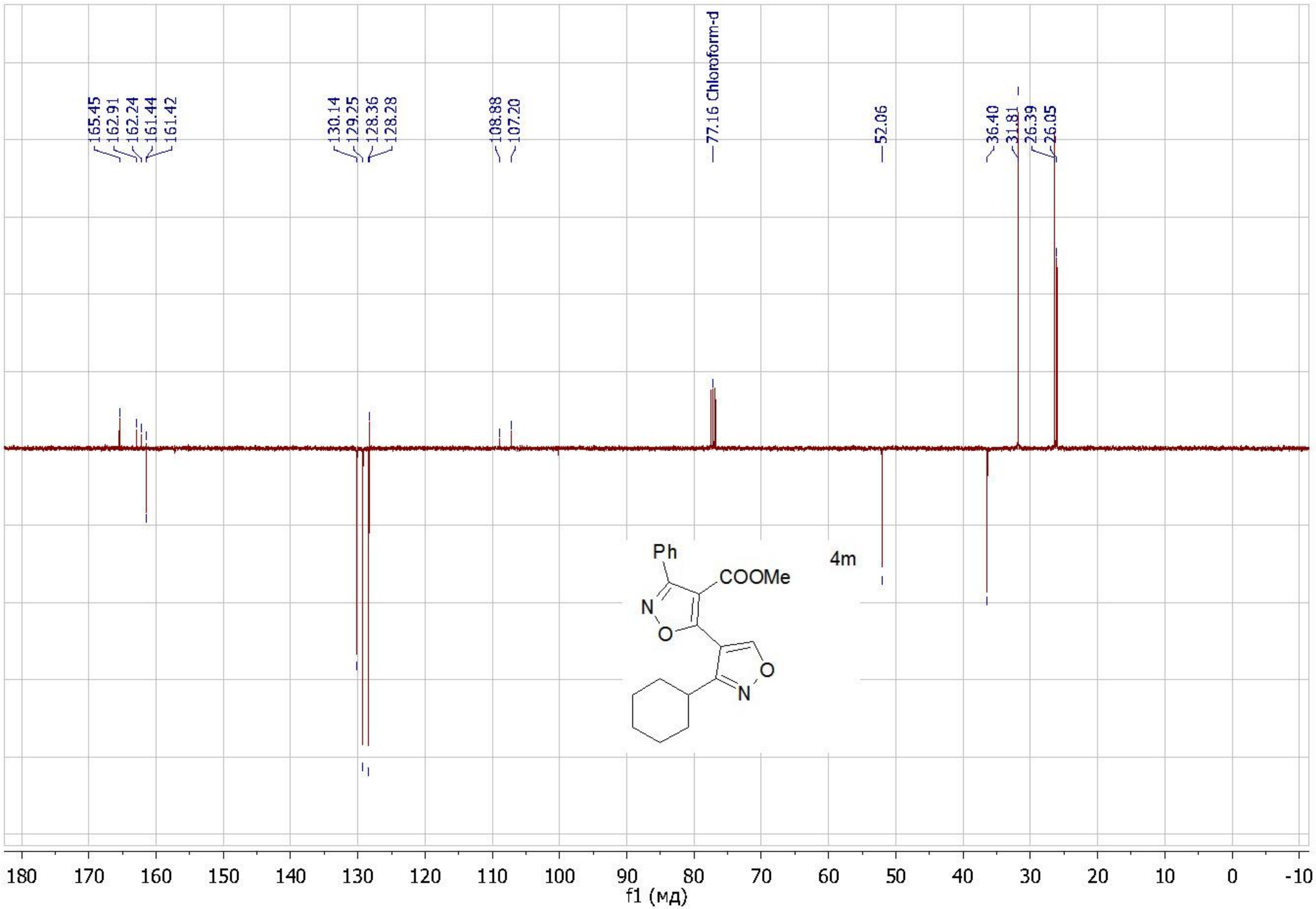
165.45
162.91
162.24
161.44
161.42
130.14
129.25
128.36
128.28
108.88
107.20
77.16 Chloroform-d
52.06
36.40
31.81
26.39
26.05
Ph
COOMe
N
O
O
N
4m
180 170 160 150 140 130 120 110 100 90 80 70 60 50 40 30 20 10 0 -10
f1 (мд)

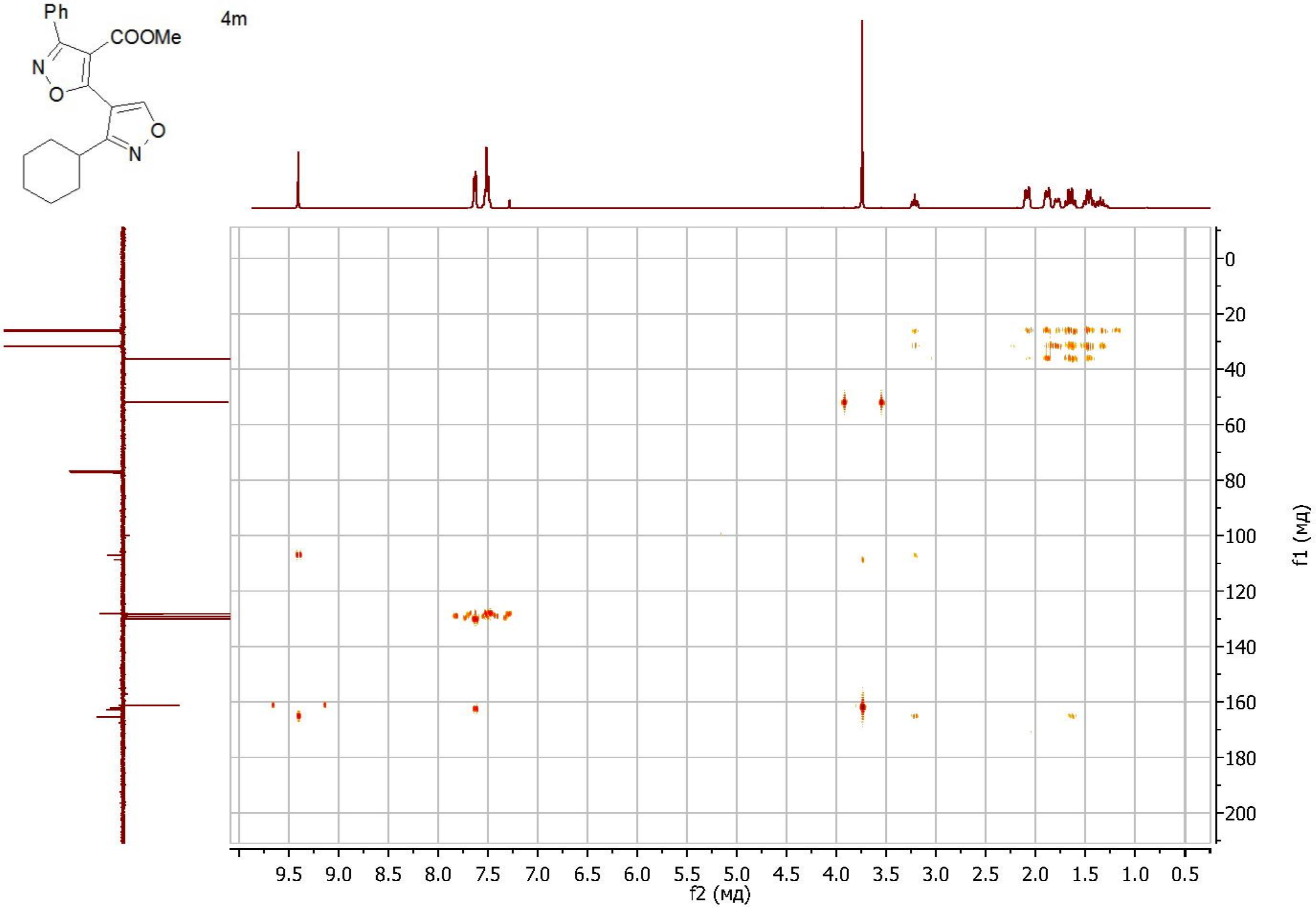
Ph
COOMe
N
O
O
N
4m
f2 (мд)
f1 (мд)

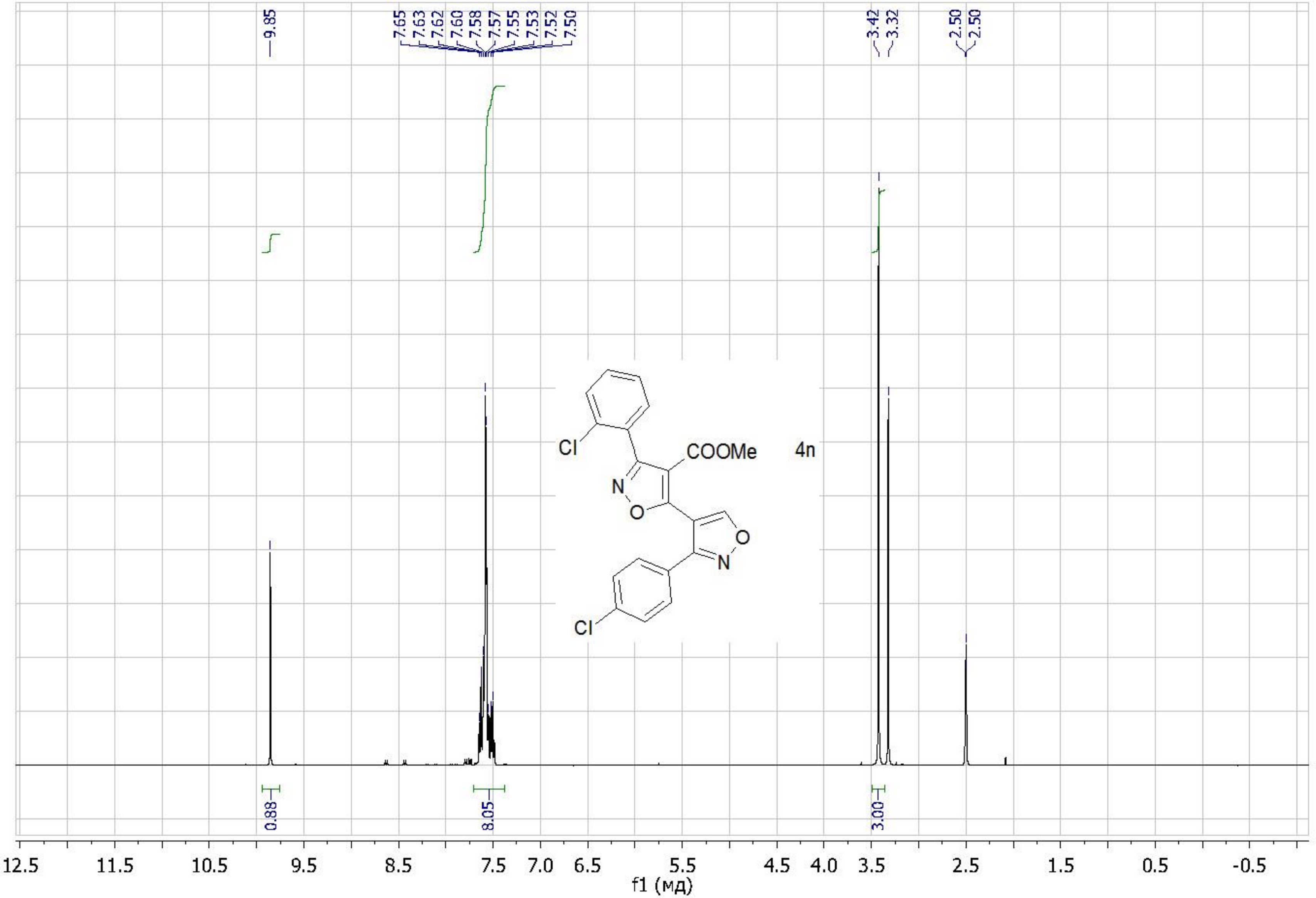
9.85
7.65 7.63 7.62 7.60 7.58 7.57 7.55 7.53 7.52 7.50
3.42 3.32
2.50 2.50
Cl
COOMe
4n
N
O
O
N
Cl
0.88
8.05
3.00
12.5 11.5 10.5 9.5 8.5 7.5 7.0 6.5 5.5 4.5 4.0 3.5 2.5 1.5 0.5 -0.5
f1 (мд)

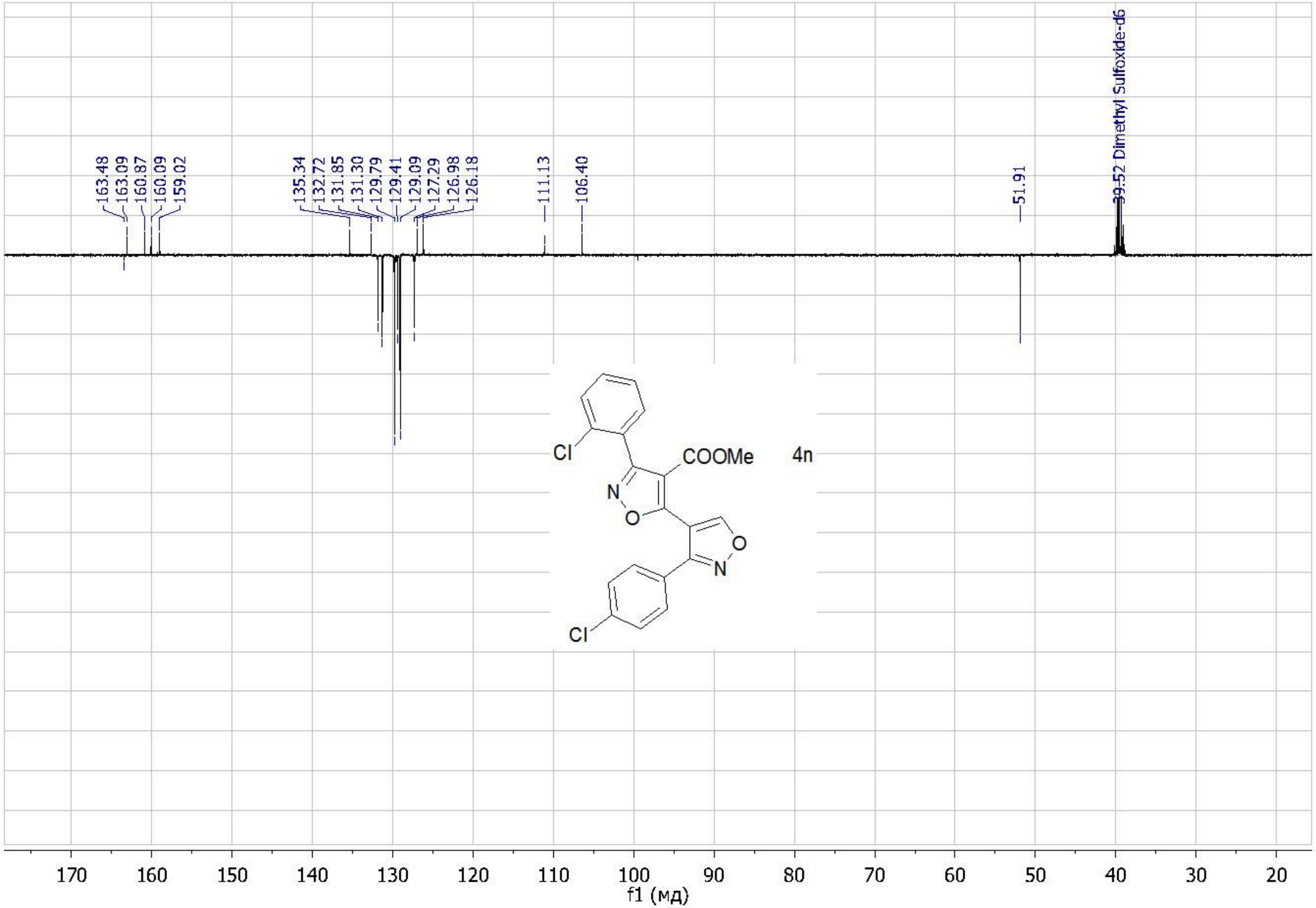
163.48
163.09
160.87
160.09
159.02
135.34
132.72
131.85
131.30
129.79
129.41
129.09
127.29
126.98
126.18
111.13
106.40
51.91
39.52 Dimethyl Sulfoxide-d6
Cl
COOMe
4n
N
O
O
N
Cl
170
160
150
140
130
120
110
100
90
80
70
60
50
40
30
20
f1 (мд)

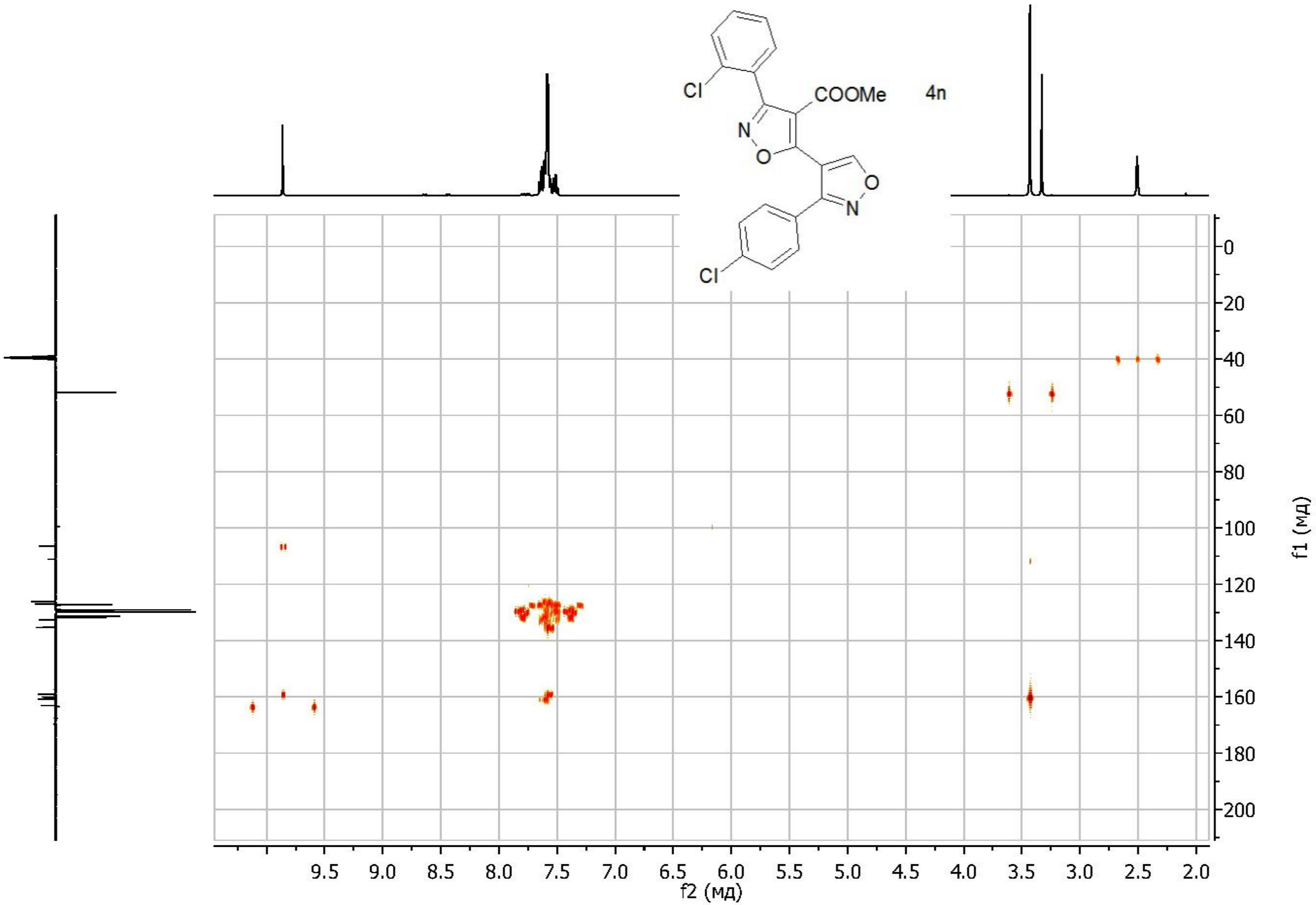

Cl
COOMe
4n
N
O
O
N
Cl
f1 (мд)
f2 (мд)

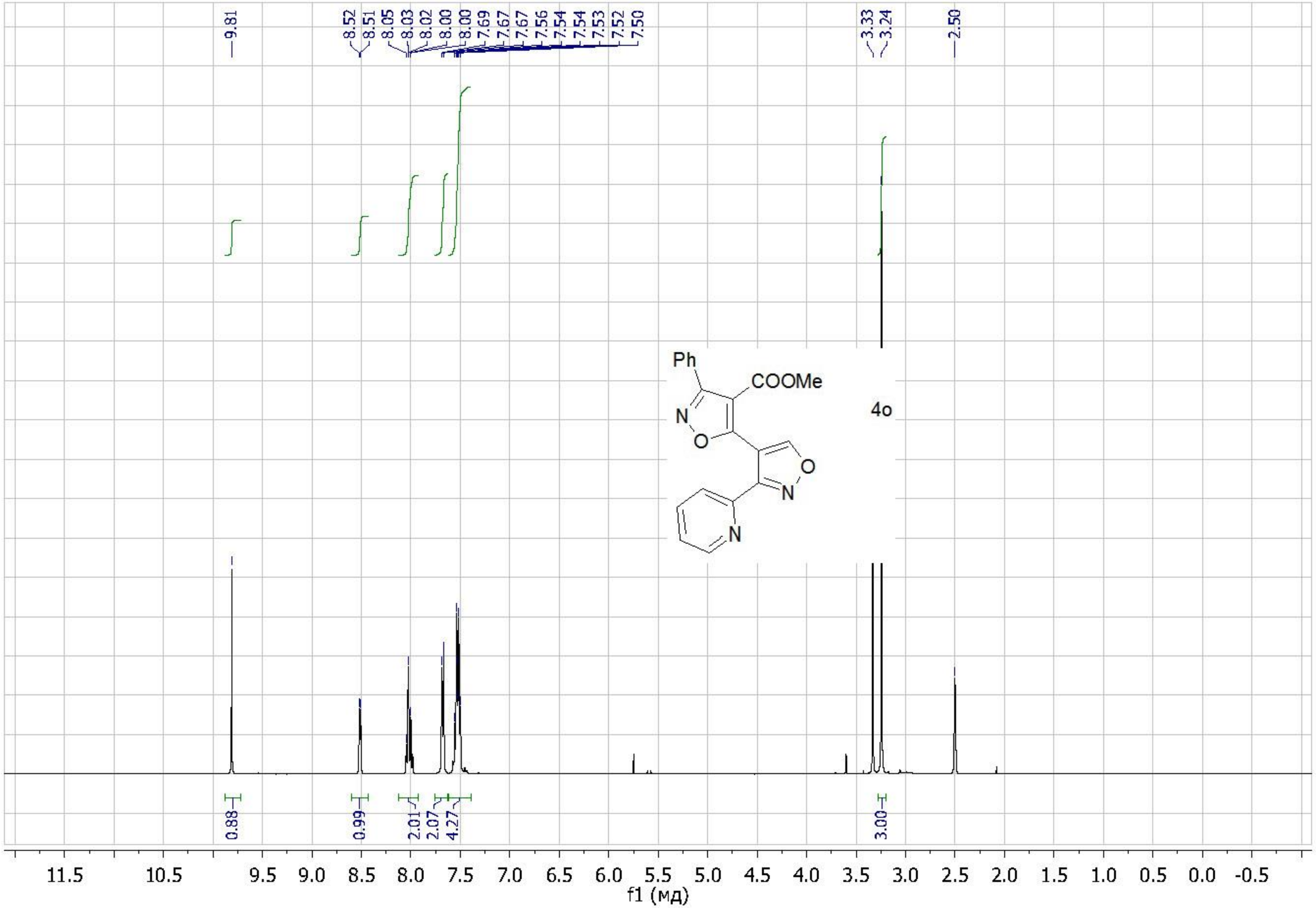
9.81
8.52
8.51
8.05
8.03
8.02
8.00
8.00
7.69
7.67
7.67
7.56
7.54
7.54
7.53
7.52
7.50
3.33
3.24
2.50
0.88
0.99
2.01
2.07
4.27
3.00
11.5
10.5
9.5
9.0
8.5
8.0
7.5
7.0
6.5
6.0
5.5
5.0
4.5
4.0
3.5
3.0
2.5
2.0
1.5
1.0
0.5
0.0
-0.5
f1 (мд)
Ph
COOMe
N
O
O
N
N
4o

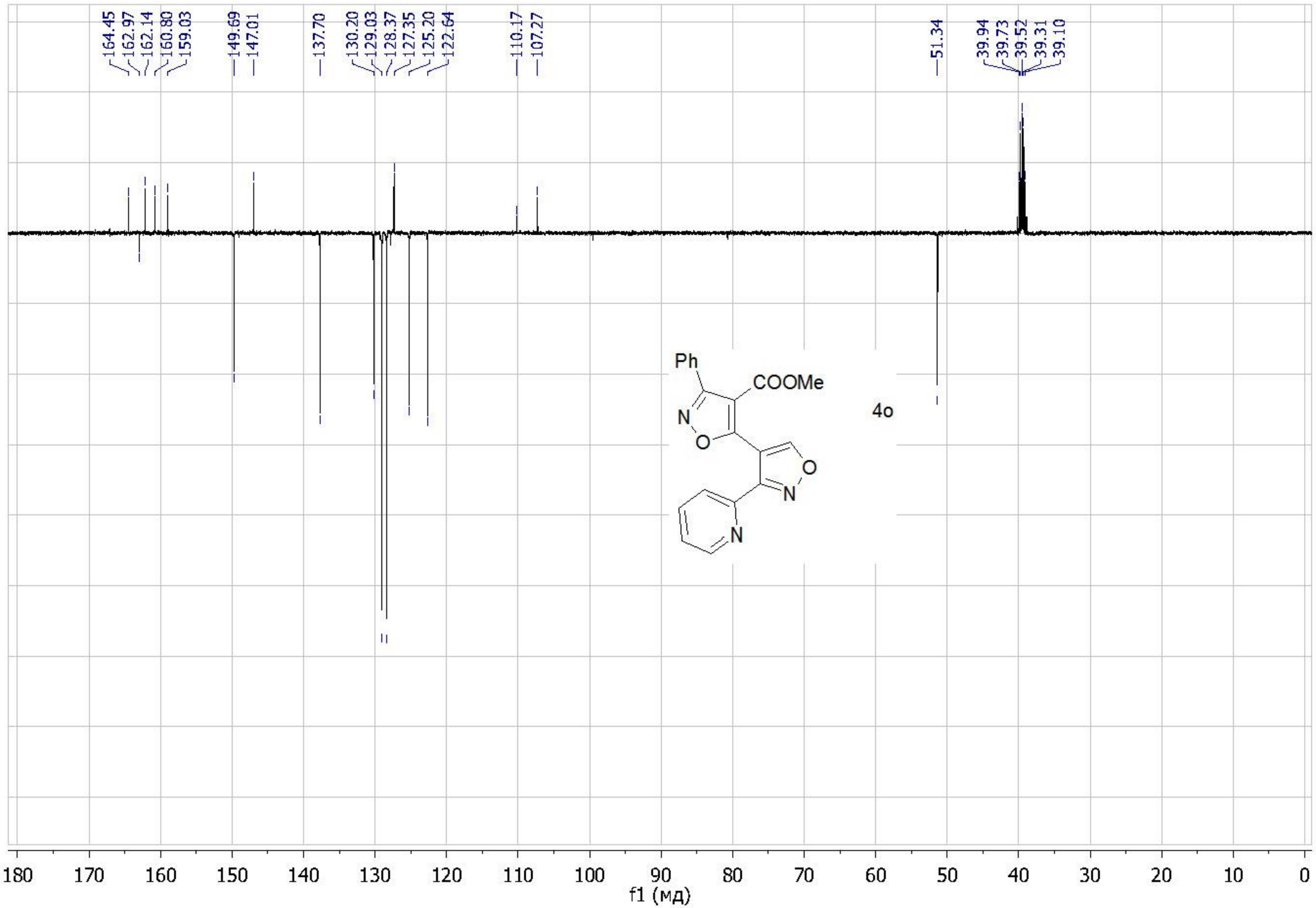
164.45
162.97
162.14
160.80
159.03
149.69
147.01
137.70
130.20
129.03
128.37
127.35
125.20
122.64
110.17
107.27
51.34
39.94
39.73
39.52
39.31
39.10
Ph
COOMe
N
O
O
N
N
4o
180 170 160 150 140 130 120 110 100 90 80 70 60 50 40 30 20 10 0
f1 (мд)

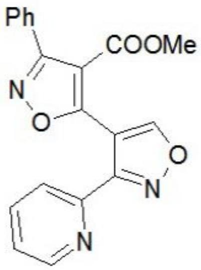
Ph
COOMe
N
O
O
N
N

4o

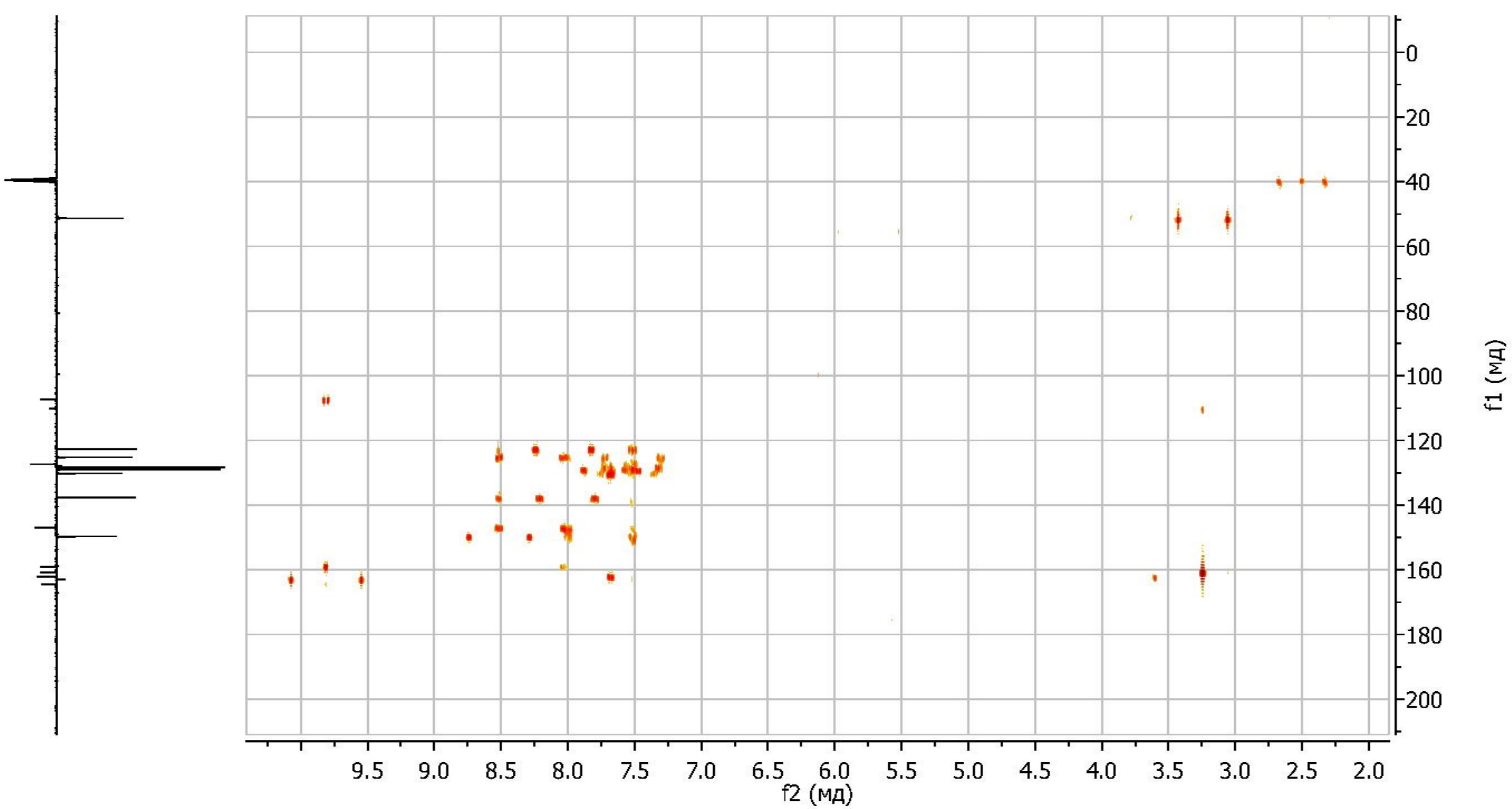
f2 (мд)
f1 (мд)
9.5 9.0 8.5 8.0 7.5 7.0 6.5 6.0 5.5 5.0 4.5 4.0 3.5 3.0 2.5 2.0
0 20 40 60 80 100 120 140 160 180 200

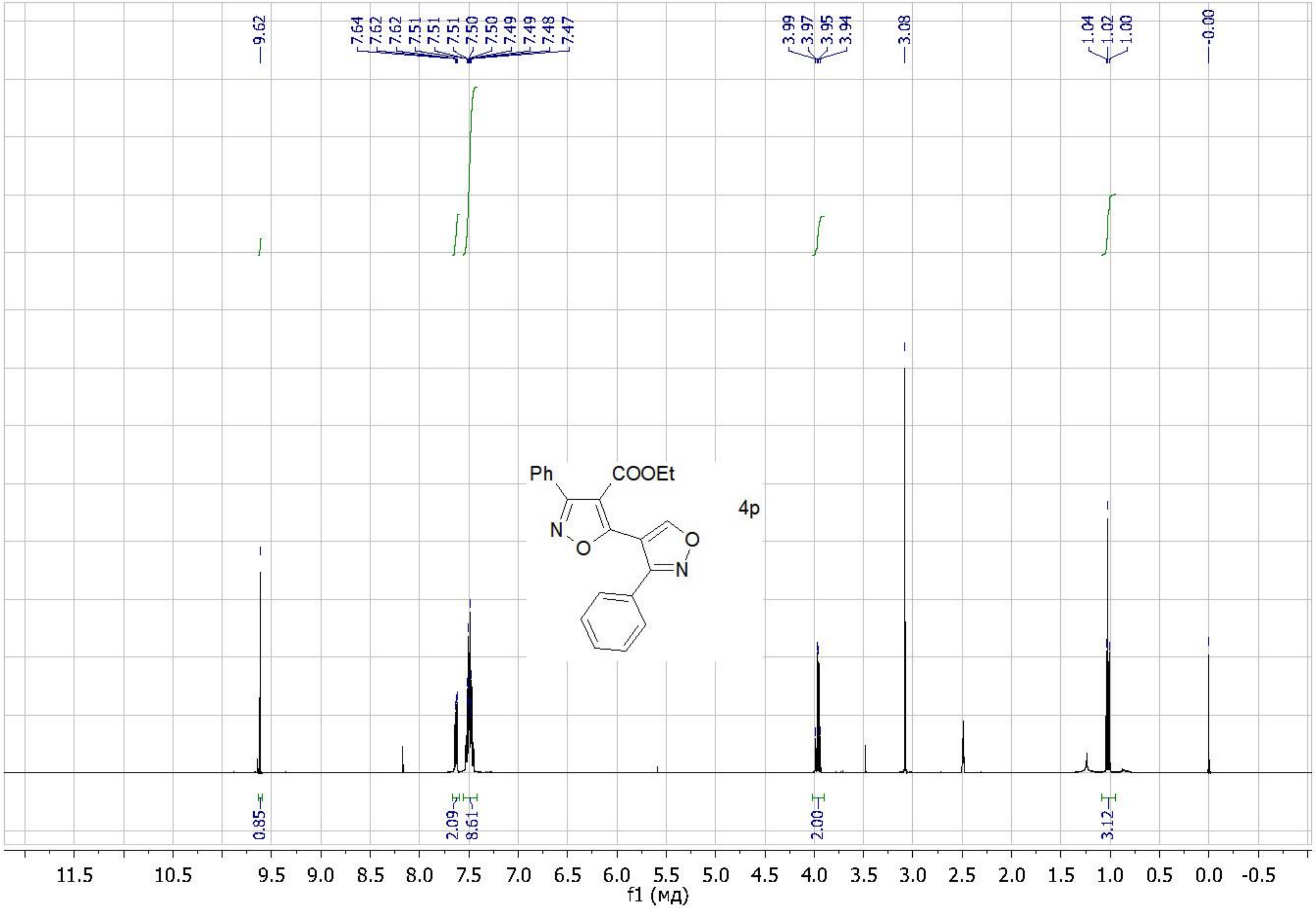
9.62
7.64
7.62
7.62
7.51
7.51
7.51
7.50
7.50
7.49
7.49
7.48
7.47
3.99
3.97
3.95
3.94
3.08
1.04
1.02
1.00
-0.00
Ph
COOEt
N
O
4p
0.85
2.09
8.61
2.00
3.12
11.5
10.5
9.5
9.0
8.5
8.0
7.5
7.0
6.5
6.0
5.5
5.0
4.5
4.0
3.5
3.0
2.5
2.0
1.5
1.0
0.5
0.0
-0.5
f1 (мд)

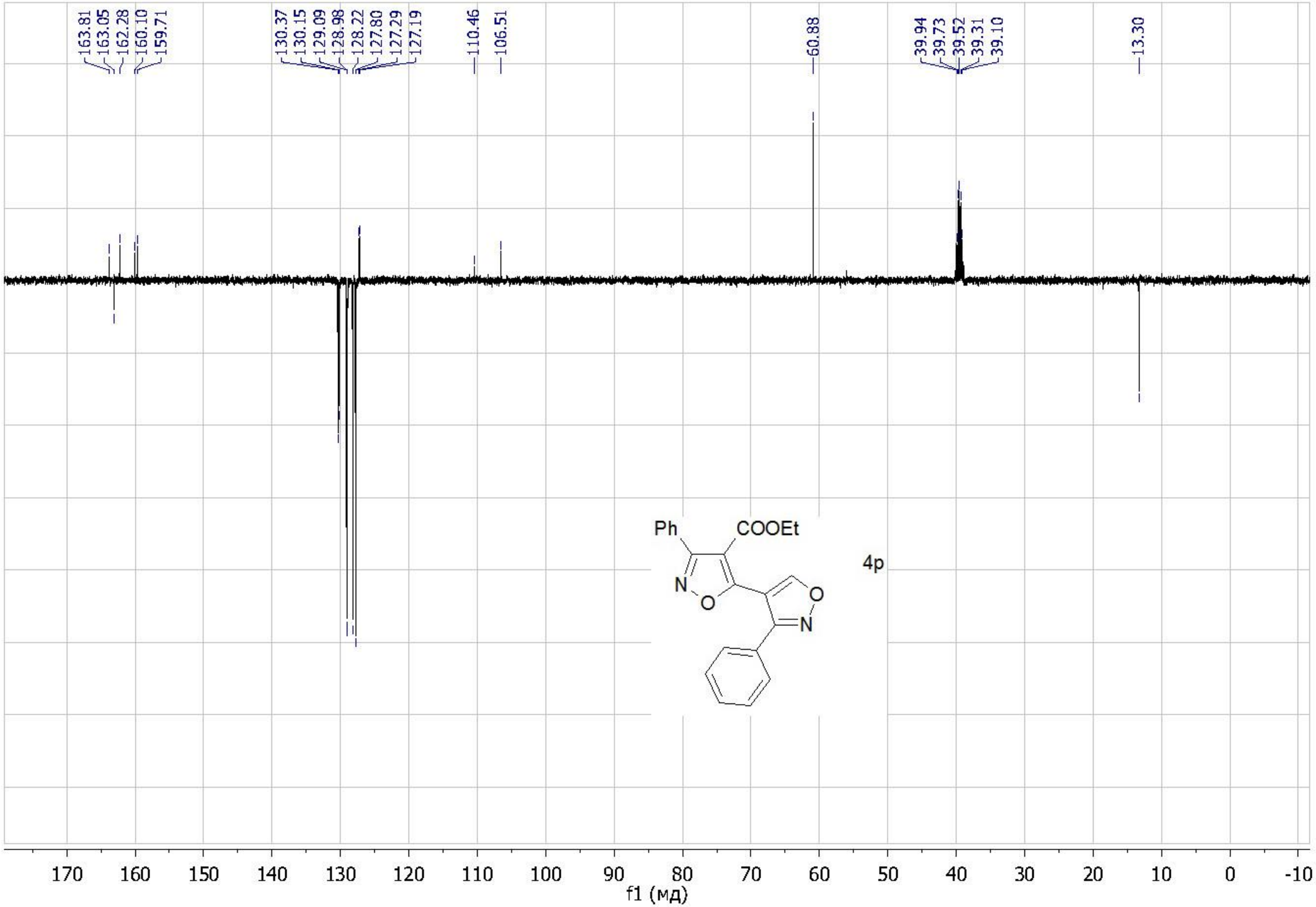

163.81
163.05
162.28
160.10
159.71
130.37
130.15
129.09
128.98
128.22
127.80
127.29
127.19
110.46
106.51
60.88
39.94
39.73
39.52
39.31
39.10
13.30
Ph
COOEt
N
O
O
N
170
160
150
140
130
120
110
100
90
80
70
60
50
40
30
20
10
0
-10
f1 (мд)

4p
